# Supplementary material for: Composition and paleoecology of the mid-Jurassic bony fish assemblage of the Vaches Noires cliffs (Normandy, France)
Source: PLoS One. 2026 May 27;21(5):e0344519. doi: 10.1371/journal.pone.0344519 (PMC13215526; doi:10.1371/journal.pone.0344519)

PLOS ONE

Composition and paleoecology of the mid-Jurassic bony fish assemblage of the Vaches Noires cliffs (Normandy, France).

--Manuscript Draft--

**Manuscript Number:**	PONE-D-25-45591
**Article Type:**	Research Article
**Full Title:**	Composition and paleoecology of the mid-Jurassic bony fish assemblage of the Vaches Noires cliffs (Normandy, France).
**Short Title:**	Composition and paleoecology of the mid-Jurassic bony fish assemblage of the Vaches Noires cliffs, France.
**Corresponding Author:**	Simon Beaufils<br>Université de Rennes: Universite de Rennes<br>Rennes, FRANCE
**Keywords:**	Vaches Noires cliffs; Callovian; Bony fishes; Coelacanths; Trophic web; Jurassic fish diversity
**Abstract:**	The Callovian fossil assemblage of the Vaches Noires cliffs in Normandy, France has played an important role in French paleontology since the end of the 18th century. The bony fish component, which includes coelacanths and ray-finned fishes, is represented mainly by fragmentary material. In this study, we provide the first overview of the bony fish assemblage by examining isolated remains collected mostly by amateur paleontologists over the years and housed in public collections. The assemblage comprises remains of coelacanths (Trachymetopon), pachycormids represented by the filter feeder Leesdichthys problematicus, and by the toothed predator cf. Hypsocormus. Ginglymodians are represented by cranial elements of cf. Scheenstia and halecomorphs by 'Eurypoma' grande. Pycnodontiforms (Mesturus) are mainly represented by isolated vomers and prearticulars. The presence of teleosteans is attested by small amphicoelous vertebrae. For each taxon, estimated body size and trophic level are inferred through comparisons with extant ecological analogs. Finally, we compare the Vaches Noires assemblage with other Jurassic fish assemblages to assess broader evolutionary and ecological trends in bony fish diversity during the Jurassic.
**Order of Authors:**	Simon Beaufils
	Romain Vullo
	Damien Gendry
	Laurent Picot
	Lionel Cavin
**Additional Information:**	
**Question**	**Response**
**Financial Disclosure**<br><br>Enter a financial disclosure statement that describes the sources of funding for the work included in this submission. Review the submission guidelines for detailed requirements. View published research articles from *PLOS ONE* for specific examples.<br><br>This statement is required for submission	Yes

Powered by Editorial Manager® and ProduXion Manager® from Aries Systems Corporation

and **will appear in the published article** if the submission is accepted. Please make sure it is accurate.<br><br>**Funded studies**<br>Enter a statement with the following details:<br>• Initials of the authors who received each award<br>• Grant numbers awarded to each author<br>• The full name of each funder<br>• URL of each funder website<br>• Did the sponsors or funders play any role in the study design, data collection and analysis, decision to publish, or preparation of the manuscript?<br><br>Did you receive funding for this work?	
Please add funding details.<br>as follow-up to "**Financial Disclosure**<br><br>Enter a financial disclosure statement that describes the sources of funding for the work included in this submission. Review the submission guidelines for detailed requirements. View published research articles from *PLOS ONE* for specific examples.<br><br>This statement is required for submission and **will appear in the published article** if the submission is accepted. Please make sure it is accurate.<br><br>**Funded studies**<br>Enter a statement with the following details:<br>• Initials of the authors who received each award<br>• Grant numbers awarded to each author<br>• The full name of each funder<br>• URL of each funder website<br>• Did the sponsors or funders play any role in the study design, data collection and analysis, decision to publish, or preparation of the manuscript?<br><br>Did you receive funding for this work?"	This work is a contribution to the project 'Burst and Stasis in morphological evolution of Mesozoic coelacanths' funded by the Swiss National Science Foundation (https://data.snf.ch/grants/grant/207903 to L.C.). The funders had no role in study design, data collection and analysis, decision to publish, or preparation of the manuscript.

Powered by Editorial Manager® and ProduXion Manager® from Aries Systems Corporation

Please select the country of your main research funder (please select carefully as in some cases this is used in fee calculation).<br>as follow-up to "**Financial Disclosure**<br><br>Enter a financial disclosure statement that describes the sources of funding for the work included in this submission. Review the submission guidelines for detailed requirements. View published research articles from *PLOS ONE* for specific examples.<br><br>This statement is required for submission and **will appear in the published article** if the submission is accepted. Please make sure it is accurate.<br><br>**Funded studies**<br>Enter a statement with the following details:<br>• Initials of the authors who received each award<br>• Grant numbers awarded to each author<br>• The full name of each funder<br>• URL of each funder website<br>• Did the sponsors or funders play any role in the study design, data collection and analysis, decision to publish, or preparation of the manuscript?<br><br>Did you receive funding for this work?"	SWITZERLAND - CH
**Competing Interests**<br><br>Use the instructions below to enter a competing interest statement for this submission. On behalf of all authors, disclose any competing interests that could be perceived to bias this work—acknowledging all financial support and any other relevant financial or non-financial competing interests.<br><br>This statement is required for submission and **will appear in the published article** if the submission is accepted. Please make sure it is accurate and that any funding	The authors declare that they have no competing interests.

sources listed in your Funding Information later in the submission form are also declared in your Financial Disclosure statement.<br><br>View published research articles from *PLOS ONE* for specific examples.<br><br>**NO authors have competing interests**<br><br>Enter: *The authors have declared that no competing interests exist.*<br><br>**Authors with competing interests**<br><br>Enter competing interest details beginning with this statement:<br><br>*I have read the journal's policy and the authors of this manuscript have the following competing interests: [insert competing interests here]*<br><br>* typeset	
**Ethics Statement**<br><br>Enter an ethics statement for this submission. This statement is required if the study involved:<br><br>• Human participants<br>• Human specimens or tissue<br>• Vertebrate animals or cephalopods<br>• Vertebrate embryos or tissues<br>• Field research<br><br>Write "N/A" if the submission does not require an ethics statement.<br><br>General guidance is provided below. Consult the submission guidelines for detailed instructions. **Make sure that all information entered here is included in the Methods section of the manuscript.**	No permits were required for the described study, which complied with all relevant regulations.

Powered by Editorial Manager® and ProduXion Manager® from Aries Systems Corporation

**Format for specific study types**<br><br>**Human Subject Research (involving human participants and/or tissue)**<br>• Give the name of the institutional review board or ethics committee that approved the study<br>• Include the approval number and/or a statement indicating approval of this research<br>• Indicate the form of consent obtained (written/oral) or the reason that consent was not obtained (e.g. the data were analyzed anonymously)<br><br>**Animal Research (involving vertebrate animals, embryos or tissues)**<br>• Provide the name of the Institutional Animal Care and Use Committee (IACUC) or other relevant ethics board that reviewed the study protocol, and indicate whether they approved this research or granted a formal waiver of ethical approval<br>• Include an approval number if one was obtained<br>• If the study involved *non-human primates*, add *additional details* about animal welfare and steps taken to ameliorate suffering<br>• If anesthesia, euthanasia, or any kind of animal sacrifice is part of the study, include briefly which substances and/or methods were applied<br><br>**Field Research**<br><br>Include the following details if this study involves the collection of plant, animal, or other materials from a natural setting:<br>• Field permit number<br>• Name of the institution or relevant body that granted permission	
**Data Availability**<br><br>Authors are required to make all data underlying the findings described fully available, without restriction, and from the time of publication. PLOS allows rare exceptions to address legal and ethical concerns. See the PLOS Data Policy and FAQ for detailed information.	Yes - all data are fully available without restriction

Powered by Editorial Manager® and ProduXion Manager® from Aries Systems Corporation

A Data Availability Statement describing where the data can be found is required at submission. Your answers to this question constitute the Data Availability Statement and **will be published in the article**, if accepted.<br><br>**Important:** Stating 'data available on request from the author' is not sufficient. If your data are only available upon request, select 'No' for the first question and explain your exceptional situation in the text box.<br><br>Do the authors confirm that all data underlying the findings described in their manuscript are fully available without restriction?	
**Describe where the data may be found in full sentences. If you are copying our sample text, replace any instances of XXX with the appropriate details.**<br><br>• If the data are **held or will be held in a public repository**, include URLs, accession numbers or DOIs. If this information will only be available after acceptance, indicate this by ticking the box below. For example: *All XXX files are available from the XXX database (accession number(s) XXX, XXX.*).<br>• If the data are all contained **within the manuscript and/or Supporting Information files**, enter the following: *All relevant data are within the manuscript and its Supporting Information files.*<br>• If neither of these applies but you are able to provide **details of access elsewhere**, with or without limitations, please do so. For example:<br><br>*Data cannot be shared publicly because of [XXX]. Data are available from the XXX Institutional Data Access / Ethics Committee (contact via XXX) for researchers who meet the criteria for access to confidential data.*<br><br>*The data underlying the results presented in the study are available from (include the name of the third party*	All relevant data are within the manuscript and its Supporting Information files.

and contact information or URL).<br>• This text is appropriate if the data are owned by a third party and authors do not have permission to share the data.<br><br>* typeset	
Additional data availability information:	

Manuscript
Click here to access/download;Manuscript;Beaufils_et_al_(2025).docx

Composition and paleoecology of the mid-Jurassic bony fish assemblage of the Vaches Noires cliffs (Normandy, France).

Simon Beaufils[1,2¶]*, Romain Vullo[1&], Damien Gendry[1&], Laurent Picot[2&], Lionel Cavin[3¶]

1 Univ Rennes, CNRS, Géosciences Rennes, UMR 6118, Rennes, France,

2 Paléospace Museum, Villers-sur-Mer, France,

3 Department of Geology and Palaeontology, Natural History Museum, Geneva, Switzerland.

*Corresponding author

E-mail : beaufils.simon42@gmail.com

[¶] These authors contributed equally to this work.

[&] These authors also contributed equally to this work.

Abstract: The Callovian fossil assemblage of the Vaches Noires cliffs in Normandy, France has played an important role in French paleontology since the end of the 18th century. The bony fish component, which includes coelacanths and ray-finned fishes, is represented mainly by fragmentary material. In this study, we provide the first overview of the bony fish assemblage by examining isolated remains collected mostly by amateur paleontologists over the years and housed in public collections. The assemblage comprises remains of coelacanths (*Trachymetopon*), pachycormids represented by the filter feeder *Leesdichthys problematicus*, and by the toothed predator cf. *Hypsocormus*. Ginglymodians are represented by cranial elements of cf. *Scheenstia* and halecomorphs by *'Eurypoma' grande*. Pycnodontiforms (*Mesturus*) are mainly represented by isolated vomers and prearticulars. The presence of teleosteans is attested by small amphicoelous vertebrae. For each taxon, estimated body size and trophic level are inferred through comparisons with extant ecological analogs. Finally, we compare the Vaches Noires assemblage with other Jurassic fish assemblages to assess broader evolutionary and ecological trends in bony fish diversity during the Jurassic.

I - Introduction

The fossil site of the Vaches Noires cliffs and surrounding regions (France, Normandy, Calvados) is important in the history of paleontology and has been studied for over 250 years (1,2). Public and private collections testify to the intense research carried out in the region since the 18th century. The first references to fossils date back from 18th century, with Abbé Jacques-Francois Dicquemare (1733-1789) who published an article entirely devoted to the «osteoliths» from the Kimmeridgian of the Cap de la Hève and the Callovian-Oxfordian series of the Vaches Noires (1,3). In the 19th century, local collectors gave Georges Cuvier access to fossil material, which allowed him to prove the existence of previously unknown extinct species (1–6). In more recent times, private collectors have continued to amass extensive fossil assemblages, further

enriching the paleontological record through their dedication to the discovery and preservation of significant specimens. Among those who made their collections available to researchers and public institutions are Elisabeth and Gérard Pennetier, Agathe and Jean Charles, Damien Gendry and Coraline Bara (7–9, this paper). Most of the published fossils from the Vaches Noires cliffs and particularly from the Marnes de Dives Formation are precisely located in the local stratigraphy, corresponding to the Upper Callovian, and the Lamberti zone. Few fossils not found in situ were removed from the study, to avoid uncertainties in the succession of the ichthyofauna assemblages. Contrary to common belief, fish remains in the Vaches Noires cliffs are abundant, but their fragmentary nature sometimes makes their systematic identification difficult.

During the Upper Callovian, central England and northern France were covered by an epicontinental sea, as evidenced today by the thick marl series (7,10–13). This gigantic mudflat open to the offshore waters received terrigenous sediments from the Armorican massif as evidenced by the Marnes de Dives Formation (7,10,11,14). The driftwood, seeds, and cones found on the rocky platform just above the vertebrates are indicators of terrigenous inputs via storms (oyster-rich limestone deposits and settling of clays with wood and seeds). The Marnes de Dives assemblage includes gastropods, bivalves, cephalopods, particularly ammonites, used for biochronological studies (10,11,14,15). Vertebrates are abundant, including cartilaginous fish (mainly hybodontiforms (16)), marine reptiles (plesiosaurs, ichthyosaurs (17) and and crocodilian (18)). Some remains of terrestrial animals such as dinosaurs have been rarely found (19–22).

Bony fish are well represented in the Vaches Noires cliffs since they include ginglymodians (Lepidotidae), pycnodonts (23,24), coelacanths (mostly isolated bones) (8,24–26), representatives of thunniform and suspension feeder pachycormids (9,27–31) and

halecomorphs (24,32). Abundant well ossified vertebrae are attributed for the first time in this deposit to basal teleosts.

The aim of this paper is on the one hand to depict the taxonomic composition and the food chain of bony fishes based on previously described material and new specimens, and on the other hand to integrate it within the whole vertebrate assemblage of the Upper Callovian of the Vaches Noires cliffs. Finally, we place and compare the trophic web within the general Mesozoic context of pelagic fish fauna in order to understand the beginnings of the Late Jurassic actinopterygians radiations (33).

II - Geological setting

The fossil site of the Vaches Noires cliffs is located in the northwest of Calvados department in France. The cliffs extend over 4.5 km of coastline between the towns of Villers-sur-Mer and Houlgate (Fig 1). The visible sedimentary series outcrops continuously from the Upper Callovian (≈163 Ma) to the Middle Oxfordian (≈ 160 Ma), the upper part of which is formed by the "coral – rag" (Tenuiserratum Zone) (10,11,34), i.e. an oyster-rich limestone facies deposited in marine subtidal proximal environments. The Albian-Cenomanian flint chalk rests in unconformity above the Jurassic deposits. The Marnes de Dives Formation is well-known for their beds rich in vertebrates dating from the Upper Callovian (Lamberti zone, Praelamberti Subzone). Approximately 15 metres of the 20-metre-thick formation are exposed (15, pers. obs. DG-LP). These marls are the French equivalent of the British Oxford Clay and outcrop in some areas, such as a rocky flat (7,8,25). On the Villers-sur-Mer side, the Upper Callovian is covered with a recent layer of sand 50 cm to 1 m thick, sometimes uncovered during high tides. Only a few *Leedsichthys* bones recovered from the Henrici Subzone complement the material described from the studied level. All other known specimens originate from the Praelamberti Subzone.The fossiliferous bed is more often exposed on the Houlgate side where several

lithostratigraphic markers allow to delimit the different beds between the soft clay-silty layers and the more indurated marl layers (10,11). Benthic bioclasts are rarely pyritized and contain brachiopods, oysters, gastropods, trigonids all associated with pieces of pyritized wood often decametric in size (10,11). An assemblage of foraminifera found associated with a vertebrate fossil from the Marnes de Dive provided an Late Jurassic age, without further precision (25). Sequential stratigraphy indicates a dynamics of High Stand System Tracts (HSST) at the end of the Callovian (11), with essentially marine influences but also significant terrigenous sedimentary components, including illite, kaolinites and smectites clays (10,11).

Fig 1 : A, geological map of northwestern France showing the location of the Vaches Noires cliffs; **B**, simplified geological map of the site of the Vaches Noires cliffs; **C**, stratigraphic profile of the Vaches Noires cliffs from Marnes de Dives Formation up to Marnes de Villers Formation, black fishes indicate vertebrate level.

III - Material and method

The list of the material studied (Table 1) includes bony fish remains available for study in the collections of the Paleospace (MPV: Museum Paléospace Villers) and the geological museum of the University of Rennes (IGR: Institut de Géologie de Rennes). I addition, we figured bony fish remains from the National History Museum of Paris (MNHN : Muséum National d'Histoire Naturelle) and a stomachal content FBS 2012.4.67.30 associated to a *Metriorhynchus* specimen FBS 2012.4.67.80, housed at la Fabrique des Savoirs (FBS) (Elbeuf, Normandy, France) (35). All the material likely comes from the Marnes de Dives Formation and was found either in situ or washed from rocks by the tides. Specimens were prepared by needle when required and MPV 2019.1.1 was prepared by dissolving the matrix with an unknown acid. Specimens were photographed with a camera Olympus TG-6 and drawings based on photographs were made

with Inkscape Vector Graphics. Little direct evidence of predator/prey relationship, such as gut contents or traces of predation evidence were available, and consequently the trophic network was reconstructed on indirect evidence such as functional morphology. Trophic levels (TL) are represented by a scale ranging from 1 (primary producers or plants) to 5 (top predators). Pauly and Palomares (36) defined the trophic level of a consumer species i, TL_i as:

$$TL_i = 1 + \sum_j (TL_j \times DC_{ij})$$

Where TL_j is the fractional trophic level of the prey j, and DC_{ij} represents the fraction of j in the diet of i. We cannot directly calculate this value for extinct species. Accordingly, we estimated the TL value of taxa from the Vaches Noires by comparison with TL provided by Fishbase (37) for extant taxa that are phylogenetically and/or ecologically closely related to the extinct taxa. Figures of the fish assemblage network is based on the model proposed by Cavin et al. (2015), Cooper et Martill (2020) et Veiga et al. (2023) (38–40). The vertebrate trophic chain composition follows the model proposed by Martill et al. (1994)(12). No permits were required for the described study, which complied with all relevant regulations.

Table 1 : Summary table of the material available for the study of the Callovian ichthyofauna from the Vaches Noires cliffs.

Taxa	Bone	Collection	Collection Number
Trachymetopon sp.	Pterygoid	Bara	**IGR 153441**
Trachymetopon sp.	Pterygoid	Bara	**IGR 153442**
Trachymetopon sp.	Pterygoid	Le Mort	**MPV 2023.2.3**
Trachymetopon sp.	Palatoquadrate	Gendry	**IGR 153443**
Trachymetopon sp.	Basisphenoid	Bara - Gendry	**IGR 153444**
Trachymetopon sp.	Basisphenoid	Bara - Gendry	**IGR 153445**
Trachymetopon sp.	Basisphenoid	Daumont	**MPV 2024.1.1**
Leedsichthys problematicus	Nodule + diverse bones	Pennetier	**Batch MPV 2021.1.3.1**
Leedsichthys problematicus	Ceratobranchial (Halved)	Pennetier	**Batch MPV 2021.1.3.2**
Leedsichthys problematicus	Ceratobranchial	Pennetier	**Batch MPV 2021.1.3.3**

Leedsichthys problematicus	Ceratobranchial (two parts)	Pennetier	**Batch MPV 2021.1.3.4**
Leedsichthys problematicus	Ceratobranchial	Pennetier	**Batch MPV 2021.1.3.5**
Leedsichthys problematicus	Hypobranchial	Pennetier	**Batch MPV 2021.1.3.6**
Leedsichthys problematicus	Hyomandibular?	Pennetier	**Batch MPV 2021.1.3.7**
Leedsichthys problematicus	Dorsal spine	Gendry	**IGR 153446**
Leedsichthys problematicus	Cranial element ?	Papazian	**MPV 2024.3.2**
Leedsichthys problematicus	Hypohyal	Daumont	**MPV 2024.1.2**
Leedsichthys problematicus	Hypohyal	Daumont	**MPV 2024.1.3**
Leedsichthys problematicus	Hypohyal	Daumont	**MPV 2024.1.4**
Leedsichthys problematicus	Ceratohyal	Daumont	**MPV 2024.1.5**
Leedsichthys problematicus	Dorsal spine	Rivette	**MPV 2024.2.1**
Leedsichthys problematicus	*Metriorhynchus* stomachal content	Fabrique des Savoirs	**FBS 2012.4.67.30**
Hyspocormus sp.	Rostrodermethmoid	Le Mort	**MPV 2019.1.1**
Hyspocormus sp.	Rostrodermethmoid	Le Mort	**MPV 2023.2.1**
Hyspocormus sp.	Dentary	Pennetier	**MPV 2022.1.23**
Hyspocormus sp.	Five coronoid fragments	Pennetier	**MPV 2022.1.23**
Hyspocormus sp.	Hypural	Le Mort	**MPV 2023.2.2**
Lepidotidae	Basioccipital	Gendry	**IGR 153447**
Lepidotidae	Ceratohyal	Pennetier	**MPV 2022.1.14**
Lepidotidae	6 ganoid scales	Pennetier	**MPV 2022.1.16**
Lepidotidae	Jaw fragment	Pennetier	**MPV 2022.1.15**
cf. *Scheenstia*	Jaw fragment	Pennetier	**MPV 2022.1.22**
Eurypoma grande	Antero-inferior skull part mold	Nicolet	**MNHN.F.JRE45 (Mold)**
Athrodon sp.	Vomer	?	**Displayed in Paleospace museum**
Athrodon sp.	Vomer	Charles	**MPV 2014.2.183**
Mesturus sp.	Neurocranium mold	Nicolet	**MNHN.F.JRE46 (Mold)**
Mesturus sp.	Dermal tessserae	Pennetier	**MPV 2022.1.17**
Teleostei indet.	31 vertebrae	Pennetier	**MPV 2022.1.13**
Teleostei indet.	33 vertebrae	Charles	**MPV 2014.2.183**
Teleostei indet.	47 vertebrae	Charles	**V176R**

IV - Systematic paleontology

Sarcopterygii Romer, 1955

Actinistia Cope, 1871

Mawsoniidae Schultze, 1993

Trachymetopon Hennig, 1951

Trachymetopon sp.

Material: Three pterygoids: IGR 153441-153442 (Bara collection) and MPV 2023.2.3 (Paleospace collections); One palatoquadrate IGR 153443 (Gendry collection); Three basisphenoids IGR 153444, IGR 153445 (Bara – Gendry collection); Basisphenoid attached to a parasphenoid remain MPV 2024.1.1

Remarks. Based on Cavin et al. (25), all small coelacanths bones are attributed to young specimens of *Trachymetopon* for the following reasons: the basisphenoids present a notochord pit ventrally visible between the two processus connectens and an inner surface carved by ridges or cavities. Remains tend to have very coarse ridges, probably trabecular bone, visible due to weathering.

Pterygoids. The three pterygoids are from the left side, and only the posterior part corresponding to the strongest ossified part of theses bones is preserved (Figs 2, 3, 4). All pterygoids present an acute angle between the posteroventral and the anteroventral edge (average: 57°) compared to larger specimens of *Trachymetopon*, such as MPV 2012.1.1, SMNS.95889 (8) and MJML K785 (41), in which this angle is much more opened (≈ 86°).

Fig 2: *Trachymetopon* sp. Photographs (left) and drawings (right) of coelacanth pterygoid IGR 153441 in left lateral view (A1, A2), in labial view (B1, B2), and pterygoid IGR 153442 in left lateral view (C1, C2), in labial view (D1, D2). **ant.rdg**, anterior ridge; **f.post.part.Ptq**, fossa for the insertion of the posterior cartilage plate of the palatoquadrate; **post.rdg**, posterior ridge ; **s.s.Pt/Q**, suture surface of the pterygoid with the quadrate; **s.s.Mpt**, suture surface of the metapterygoid. Scale bar: 5 mm.

Fig 3: *Trachymetopon* sp. Palatoquadrate IGR 153443; photograph (A1) and drawing (A2) of the palatoquadrate in posterior view. **art.s.Q**, articular surface of the quadrate; **Pt**, pterygoid; **Q**, Quadrate; **s.Pt/Q**, suture of the pterygoid with the quadrate. Scale bar: 5 mm.

Fig 4: *Trachymetopon* sp. Pterygoid MPV 2023.2.3 in left lateral view (A1, A2) and labial view (B1, B2) **ant.rdg**, anterior ridge ; **f.post.part.Ptq**, fossa for the insertion of the posterior cartilage plate of the palatoquadrate; **post.rdg**, posterior ridge ; **s.s.Pt/Q**, suture surface of the pterygoid with the quadrate; **s.s.Mpt**, suture surface of the metapterygoid; **t.p,** tooth plate. Scale bar: 5 mm.

On IGR 153441-153442 (Fig 2) and MPV 2023.2.3 (Fig 4) a posterior ridge originates just dorsally to the quadrate insertion surface. This ridge delimits dorsally the posterior margin of the metapterygoid. Another ridge located anteriorly from the first delimits a pit. The latter is the insertion point for the jaw adductor muscles in coelacanths (42). The bone surface of IGR 153441-153442 presents ridges. On IGR 153442 (Fig 2C and D) and MPV 2023.2.3 (Fig 4) a small surface bearing infra-millimetrical teeth is present on the lingual side.

Basisphenoids. The basisphenoid is a strongly ossified bone that is therefore easily preserved. In coelacanths, it forms the anterior element of the intracranial joint between the ethmosphenoid (anterior) and the otoccipital (posterior) parts. Specimens IGR 153444 and IGR 153445 (Figs 5 and 6) have well-developed sphenoidal condyles. They are triangular in IGR 153444 (Fig 5) and rather rounded in IGR 153445 (Fig 6). The condyles are separated from each other by a wide notch comparable to the one present in the large specimen MNH GEPI V5778 (25). The antotic processes expand dorsolaterally with part of the sutural surface with the posterior parietal preserved. The left antotic process is preserved in IGR 153444 while the right one is preserved in IGR 153445.

The dorsum sellae forms a low wall that ventrally limits the opening of the cranial cavity. The processus connectens are elongated with parallel margins, and curve slightly antero-ventrally in their central part. They reach the ventral side of the bone where the parasphenoid was sutured, visible in specimen IGR 153444 (Fig 5). MPV 2024.1.1 (Fig 7) is the most complete of all specimens in which all previously described characters are visible, plus a part of the parasphenoid. A groove connected to the pituitary fossa runs along the dorsal part of the parasphenoid. On its ventral part, small teeth are present on the preserved part of the bone. The left lateral ascending wing is partially preserved but the part that connected to the lateral ethmoid is destroyed.

Fig 5: *Trachymetopon* sp. Basisphenoid IGR 153444: photograph (left) and drawing (right) of basisphenoïd in left lateral view (A1, A2), dorsal view (B1, B2), postero-ventral view (C1, C2), antero-dorsal view (D1) and ventral view (E1, E2). **ant.pr**, antotic process; **d.s**, dorsum sellae; **f.p**, foramen for the profundus; **not.pit**, notochord pit; **pit.fos**, pituitary fossa; **pr.con**, processus connectens; **sph.c**, sphenoid condyles; **s.s.p.Pa**, suture surface with post parietal, **spt.foss**, suprapterygoid fossa. Scale bar: 5 mm.

Fig 6: *Trachymetopon* sp. Basisphenoid IGR 153445: photograph (A) and drawing (B) of basisphenoid in posterior view. **ant.pr**, antotic process; **d.s**, dorsum sellae; **sph.c**, sphenoid condyles. Scale bar: 5 mm

Fig 7: *Trachymetopon* sp. Basisphenoid MPV 2024.1.1: photograph (left) and drawing (right) of basisphenoid in antero-dorsal view (A1, A2), postero-ventral (B1, B2). **ant.pr**, antotic process; **d.s**, dorsum sellae; **not.pit**, notochord pit; **Pas:** Parasphenoid**; Pas.t:** Parasphenoidal

teeth; **pit.fos**, pituitary fossa; **s.l.B/P**, suture line between Basisphenoid ans Parasphenoid; **pr.con**, processus connectens; **sph.c**, sphenoid condyles. Scale bar: 5 mm.

Actinopterygii Cope, 1887

Pachycormiformes Berg, 1937

Pachycormidae Woodward, 1895

Remarks. The Pachycormidae are divided into two subfamilies: Hypsocorminae and Asthenocorminae (43), the latter comprising 'Suspension Feeder Pachycormidae' (SFPs). Recent phylogenetic analyses indicate that pachycormids are closely related to teleosts, grouping them together within Teleosteomorpha. (44–47).

Asthenocorminae Cooper, Giles, Young, Maxwell, 2022

***Leedsichthys* Woodward, 1889**

***Leedsichthys problematicus* Woodward, 1889**

Material: Nine pieces referred to *L. problematicus* MPV 2021.1.3 (Paleospace collections) (One nodule MPV 2021.1.3.1, Four ceratobranchial parts MPV 2021.1.3.2 to 5 (with 2021.1.3.3 identified by Liston (27)**), one hyomandibular? MPV 2021.1.3.6, one hypobranchial MPV 2021.1.3.7); One part of a dorsal fin IGR 153446 (Gendry collection); An undetermined opercular or cranial bone MPV 2024.3.2; Ceratohyal MPV 2024.1.5; Three hypohyals MPV 2024.1.2, MPV 2024.1.3 and MPV 2024.1.4; Stomachal content from a *Metriorhynchus* FBS 2012.4.67.30 (La Fabrique des Savoirs collections).**

Remarks: For over 100 years, remains of *L. problematicus* have been found in the British Oxford Clay (3,27,48), the Vaches Noires cliffs (9), and also in Chile (Atacama Desert) (9,49).

Leedsichthys problematicus, Woodward, 1889 (50) belongs to the SFPs, with other genera such as the Toarcian *Pachycormus* Agassiz, 1833 (51), *Martillichthys* Liston, 2008 (Upper Callovian), *Asthenocormus* Woodward, 1895 (Tithonian), *Bonnerichthys* Friedman, Shimada, Martin, Everhart, Liston, Maltese, and Triebold, 2010 and *Rhinconichthys* Friedman, Shimada, Martin, Everhart, Liston, Maltese, and Triebold, 2010 (Cretaceous). Bones from this species have long been confused with parts of thyreophoran dinosaurs, including *Lexovisaurus* (parietals confused with parts of armor plates, and ceratobranchial confused with caudal spines) (27,53). There is currently only one species of *Leedsichthys*, *L. problematicus* (27). In Normandy, the genus *Leedsichthys* is reported from the Bathonian to the Kimmeridgian (54).

Gill rackers. MPV 2021.1.3 is a nodule that measures 123 mm by 105 mm containing gill rackers of *L. problematicus* (Fig 8) (MPV 2021.1.3), as well as a tooth of the shark *Notidanoides* and on the back of the nodule, one ammonite *Kosmoceras spinosum*. The latter defines the Praelamberti Subzone in the Lamberti Zone. The nodule contains 4 fragments of gill rakers. Gill rackers 1 and 2 are the best preserved, measuring 62 mm and 60 mm maximum respectively. They consist of a base, a branch and fimbriations, fringes are inserted between the dental needles. FBS 2012.4.67.30 (Fig 9), corresponds to a stomachal content from a *Metriorhynchus* (Thalattosuchia) with two gill rakers, 37 mm and 58 mm in length. Gill rakers 3 and 4 are poorly preserved. As observed in FBS 2012.4.67.30 and MPV 2021.1.3, there is interfanuncular mesh fragments *sensu* Liston 2013 and interfanuncular *sensu* Liston (9,55,56). Structures like this linked gill rakers together to form a functional sieve (55). This piece has been recently described in a study on the diet of *Metriorhynchus* (35).

Fig 8: ***L. problematicus*. MPV 2021.1.3.1**. photograph (A) and drawing (B) of the gill rakers nodule. **b**, base; **fim.**, fimbriation; **g.r.**, gill raker; **i.f.m**, interfanuncular mesh fragment; **s**, shell fragment?, undetermined element. Scale bar: 20 mm.

Fig 9: Metriorhynchidae stomachal content FBS 2012.4.67.30 (Fabrique des Savoirs): Photograph (A) and drawing (B) of stomachal content nodule of a Thalatthosuchian (Metriorhynchidae) containing gills rakers of *L. problematicus*. **b**, base; **fim**, fimbriations ; **g.r.**, gill rakers ; **i.f.m**, interfanuncular mesh fragment ; **s**, shell fragment. Scale bar 2 cm.

Ceratobranchials. Ceratobranchial specimens MPV 2021.1.3.2 to 2021.1.3.5 are illustrated in Fig 10 (A - D), including a fragment cut in half used for making thin sections figured by Liston 2009 (27) (Fig 10A1 and A2). A second halved ceratobranchial fragment was analyzed to assess its uncrushed triangular shape. Several additional fragments are attributed to *L. problematicus* ceratobranchials based on their characteristic triangular cross-section. These bones served as attachment sites for gill rakers.

Hypobranchial. A fragment of a right hypobranchial MPV 2021.1.3.6 (Fig 10E) from the anterior part of the gill basket (identified by comparison with BMNH P. 10156 (27)). This 246 mm long bone is well ossified. The anterior part, more massive, is relatively well preserved.

Figure 10 : ***L. problematicus*** **MPV 2021.1.3 batch and dorsal fin ray fragment IGR 153446 (H):** A, MPV 2021.1.3.2. Ceratobranchial in transverse (A1) & lateral (A2) views (identified by Liston (28)); B. MPV 2021.1.3.3. Ceratobranchial in lateral view (identified by Liston (28)); C, MPV 2021.1.3.4. Ceratobranchial in lateral view; D, MPV 2021.1.3.5. Ceratobranchial in lateral view (two parts); E, MPV 2021.1.3.8. Hypobranchial in lateral view; F, MPV 2021.1.3.7. Hyomandibular fragment?; G, IGR 153446 dorsal spine.
Scale bar: 5 cm.

Cranial element? Probable cranial elements of *L. problematicus*. MPV 2021.1.3.7 (Fig 10F) and MPV 2024.3.2 (Fig 11). MPV 2021.1.3.7 is 145 mm in length, but more fragmentary than MPV 2024.3.2 which is 365 mm in length. The two bones have a central hump often observed in dermal bones of *L. problematicus* (27,57). In each bone, striations radiate from the ossification center to the external peripheral shelf that is blade-like. This feature is more clearly visible in MPV 2024.3.2, which is significantly more complete, whereas in MPV 2021.1.3.7, the borders are fragmented.

Fig 11: *L. problematicus* Cranial element? MPV 2024.3.2. A, in lateral view; B, in medial view. Scale bar: 10 cm.

Hypohyals: Three hypohyals (Fig 12), one right MPV 2024.1.2 (Fig A1, B1), two left MPV 2024.1.3 (Fig A2, B2) and MPV 2024.1.4 (Fig A3, B3). They were identified by comparison with hypohyals of *Leedsichthys* described by Liston (57). Theses bones are well-ossified. MPV 2024.1.2 is 190 mm in length, MPV 2024.1.3 is 182 mm in length, MPV 2024.1.4 is 113 mm in length. The ossification state of MPV 2024.1.2 is stronger than MPV 2024.1.3. MPV 2024.1.4 is broken but preserves the anterior part. The dorsal surface of all three specimens is highly ridged anteriorly, with a smooth ventral surface. Three characteristic medial indentations give rise to a shelf that extends antero-ventrally along part of the medial edge.

Fig 12: *L. problematicus* right hypohyal MPV 2024.1.2, left hypohyal MPV 2024.1.3 and left hypohyal MPV 2024.1.4 MPV 2024.1.2 A1, in dorsal view; B1, in ventral view. MPV 2024.1.3 A2, in dorsal view; B2, in ventral view. MPV 2024.1.4 A3, in dorsal view; B3, in ventral view. **I**, Indentations. Scale bar: 5 cm.

Ceratohyal: A ceratohyal (Fig 13) MPV 2024.1.5 identified based on its typical centrally narrowed shape and in comparison with material described by Liston (27,29,57). MPV 2024.1.5 is 330 mm in maximum length but broken at both anterior and posterior ends. It has a minimum width of 110 mm. The thickest border is the superior part of the bone, the inferior blade-like part runs longitudinally. The bone is rugose on both surfaces, due to the mesh-like ossification-type typical of *Leedsichthys.* A pattern of striations radiates from the center of the convexly curved outer surface. The internal surface is flat. The bone is teardrop-shaped in transverse view.

Dorsal fin spines: Two dorsal fin spines fragments, MPV 2024.2.1 and IGR 153446, have been identified as belonging to *Leedsichthys problematicus* based on comparative morphology with previously described material, particularly specimens SMC J46873 for MPV 2024.2.1 (29) and BMNH P.6925, BMNH P.6928 (27,28,57) for IGR 153446. The spine MPV 2024.2.1, which measures 440 mm in length, is conical, anteroposteriorly compressed, and exhibits a slight curvature. In contrast, IGR 153446 is notably shorter, 114 mm, and has a more circular cross-section. Both spines display a rugose surface texture and a distinctive mesh-like ossification-type typical of *Leedsichthys.* The position of the spine in the animal body can't be inferred with information's at our disposal.

Fig 13: ***L. problematicus*** **right ceratohyal MPV 2024.1.5.** A, in lateral view; B, in medial view. Scale bar: 10 cm.

Fig 14: ***L. problematicus*** **fin spine MPV 2024.2.1.** A, in lateral view; B, in medial view. Scale bar: 10 cm.

Hypsocorminae Cooper, Giles, Young, Maxwell, 2022

Hypsocormus Wagner, 1863

Remarks. The second pachycormid clade present in the assemblage is the one composed exclusively of macrocarnivorous fishes. This clade contains thunniform species, such as the Middle/Late Jurassic *Hypsocormus* or the Late Cretaceous swordfish-like *Protosphyraena* Leidy, 1857 (58). We attribute the rostrodermethmoid to cf. *Hypsocormus* sp. because of the typical triangular shape of the bone complex, the peculiar dentition composed of very robust teeth and the stratigraphical position that fits with that of *Hypsocormus* (late Middle Jurassic (59)). *Martilichthys* (Liston, 2008) and *L. problematicus* are dismissed because of their edentulous nature. The rostrodermethmoid is a specific bone resulting either from the fusion of ethmoid and vomer (60–63), or from the fusion of rostral and dermethmoid (46).

cf. *Hypsocormus* sp.

Material: Rostrodermethmoids MPV 2019.1.1 and 2023.2.1; Batch MPV 2022.1.23 comprising dentary part (MPV 2022.1.23.1), 5 coronoids fragments (MPV 2022.1.23.2 to 2022.1.23.5) (and one tooth not figured here MPV 2022.1.23.6) and one coronoid fragment (MPV 2022.1.23.7); One hypural bone MPV 2023.2.2.

Rostrodermethmoids. Specimen MPV 2019.1.1 (Fig 15) is identified as a rostrodermethmoid by comparison with PV P 6916 (3) and PV P 6913. The specimen measures 102 mm long and 71 mm wide maximum.

A series of large teeth, of which only the tooth base or the corresponding empty sockets are preserved, extends on the lateral edge of the ossification. Traces of eight of these large teeth are visible on the right side and six on the left. They are arranged irregularly, with some teeth located offset mesially than the main alignment. The space left labially is occupied by smaller teeth, of variable size and irregularly arranged.

A bony fragment located in the matrix that fills the specimen may be part of the left antorbital, as seen in a more complete three-dimensional specimen of *Pachycormus* from the Toarcian (51).

Fig 15: cf. *Hypsocormus* sp. rostrodermethmoid MPV 2019.1.1: photograph (A) and drawing (B) of the cf. *Hypsocormus* rostrodermethmoid in ventral view. **ant**, antorbital; **t**, teeth; **t.s.**, teeth sockets. Scale bar: 10 mm.

Fig 16: cf. *Hypsocormus* sp. rostrodermethmoid MPV 2023.2.1: photographs (A1 and B) and drawing (A2) of the cf. *Hypsocormus* rostrodermethmoid in ventral view (A1, A2) and dorsal view (B). Scale bar: 5 mm

Specimen MPV 2023.2.1 is identified as a rostrodermethmoid by comparison with PV P 6916 (3) and PV P 6913. This smaller rostrodermethmoid measures a maximum of 40 mm in length and 38 mm in width (Fig 16). Two proportionally large paramedial teeth profoundly anchored in bone are visible on both sides of the symmetry axis (Fig 16 A1 and A2). There is a well-developed symphyseal tooth at the front, anteriorly oriented. Numerous small, staggered lateral teeth are present, primarily along the right labial margin. The dorsal side bears an ornamentation made of small wrinkles (Fig 16B).

Jaw fragments. Six fragments are available from MPV 2022.1.23.1 to 5 and MPV 2022.1.23.7 (Figs 17, 18, 19). We attribute them to cf. *Hypsocormus* sp. by comparison with BMNH PV P 6916 (50). Discovered close to each other, they probably belong to the same individual. MPV 2022.1.23.1 (Fig 17), identified as a piece of dentary (62), is 73 mm long and

9 mm wide at its widest level. It is probably a left dentary, given the curvature of the bone and the way it refines in dorsal view.

Fig 17: *cf. Hypsocormus sp.* Dentary MPV 2022.1.23.1: photograph (up) and drawing (down) of a cf. *Hypsocormus* dentary in dorsal view. **t**, teeth sockets. Scale bar: 10 mm.

Fig 18: *cf. Hypsocormus sp.* Coronoids fragments MPV 2022.1.23.2 (A) to 2022.1.23.5 (D): photograph in lingual view (1) and labial view (2). Scale bar: 5 mm.

Fig19: *cf. Hypsocormus sp.* Coronoid fragment MPV 2022.1.23.7: photograph (left) and drawing (right) in (probably left ?) labial view (A), ventral view (B) and lateral view (C). Scale bar: 20 mm.

Twelve teeth and seven empty sockets are visible. Teeth seems to be almost fused with the bone. They are well spaced, more in the posterior part than in the anterior part of the ossification (62). Five other fragments MPV 2022.1.23.2 to 5 (Fig 18) and MPV 2022.1.23.7 (Fig 19) are identified as pieces of coronoids that still bear teeth. Small, 2 to 4 mm high marginal teeth are visible on the labial edge of some of the coronoid fragments (Fig 18B, C and D and Fig 19). The main teeth are large, thick, and their crown is 10 mm to 20 mm high. In apical view, they have a sub-oval shape, and their base is funnel like. The surface of all teeth is striated longitudinally. The most distal part of teeth seems truncated, a consequence of the acrodine cap departure. An enamel layer seems to have peel off on MPV 2022.1.23.7, on the two bigger teeth.

Hypural bone. An almost complete compound hypural plate MPV 2023.2.2, 18 mm long and 19 mm height at maximum, is identified by comparison with hypural plate from, and attributed to *Hypsocormus posterodorsalis* in maxwell 2020 and 2025 (64,65). This plate-like triangular

bone formed the central part of a caudal fin from an indeterminate Pachycormidae. Figure 20 shows the groove for what we supposed to be the *arteria pinnalis* (Fig 20B) (66) which runs on the upper front part of the hypural bone. The parhypural foramen opens at the level of fusion of the parhypural centrum with the compound hypural plate.

Fig 20: ***cf. Hypsocormus sp.*** **Hypural plate MPV 2023.2.2.** In red, path of the *arteria pinnalis*. Scale bar: 5 mm

Ginglymodi Cope, 1872

Lepisosteiformes Hay, 1929

Lepidotidae Owen 1860 (*sensu* López-Arbarello, 2016 (67)**)**

Genus and species indeterminate

Material: A basioccipital IGR 153447; A ceratohyal MPV 2022.1.14; A batch of 6 ganoid scales MPV 2022.1.16; A jaw fragment MPV 2022.1.15; A jaw fragment MPV 2022.1.22

Cranial elements. Basioccipital IGR 153447 is 24 mm long (Fig 21A). It was identified by comparison with *Lepidotes toombsi* (A species considered by Schröder et al. (2012) as a semionotiform and included in the genus *Macrosemimimus* (68)) described in detail by Patterson (69). The occipital condyle incorporates at least one vertebra, as indicated by the pair of facets for ribs present posteroventrally to the condyle. The ventral part shows the sutural surface to the postero-dorsal part of the parasphenoid. The left-wing extension that was sutured to the exoccipital is preserved. The latter forms a 70° angle to the vertical axis of the bone.

The quadrate MPV 2022.1.14 (Figs 21B and C) is identified by comparison with the quadrate PV P 6841 (3) of *Lepidotes latifrons* Woodward, 1893 from BMNH collections and USNM 279856 of « *L.* ». *gloriae* in Thies 1989 (70). MPV 2022.1.14 measures 53 mm in length

and 55 mm in maximum height. The anterior lower part is developed in a massive condyle that articulated with the lower jaw. The bone is ornamented with fine striations radiating from the condyle to the rounded upper margin. The lateral surface bears a wing like extension that form a shelf and a semi-cylindrical channel. The medial surface bears a shallow crest with a semi-cylindrical channel.

Fig 21: Lepidotidae indet. Cranial elements: basioccipital (A) IGR 153447 & Quadrate (B & C) MPV 2022.1.14: photograph (A1) and drawing (A2) of the basioccipital IGR 153447 in posterior view. Photograph (C1, B1) and drawing (C2, B2) of quadrate MPV 2022.1.14 in labial view (B) and left lateral view (C). **C.a.Art**, Condyle for articulation with Articular; **s.s.Qj**, suture surface with the Quadratojugal; **f.r**, facet for ribs **s.pro.exo**, suture process with exoccipital. Scales bars: 10 mm

Fig 22: Lepidotidae indet. Ganoid scales MPV 2022.1.14: Photograph (A1, B, C, D1, E, F) and drawings (A2 and D2) to show the peg and socket articulation. Scale bar: 10 mm.

Ganoid Scales. Six scales that have a typical "peg and socket" joint (Fig. 22). The anterior portion of the scales, which was covered by the posterior part of the scale anterior to it, has no ganoin and is composed of a double (lower and upper) anterior articular process, as well as a small dorsal process. The posterior margin of ganoin cover of the scales has a finely fringed edge.

Scheenstia **López-Arbarello & Sferco, 2011**

cf. *Scheenstia* sp.

Material: a dentary MPV 2022.1.15; an undetermined jaw fragment MPV 2022.1.22.

Jaw fragments. The dentary (Fig. 23A) is 150 mm long and 40 mm high. Identified by comparison with material described in (71) and (72). It has an arched morphology in lateral view. A series of pores of the mandibular sensory canal runs in the mid-depth of the dentary along on the anterior half of the bone. There are four complete teeth left, three broken and two alveoli. Teeth are styliform and relatively high with a maximum height of 6.1 mm and a maximum diameter of 1.5 mm at the base. The attachment of teeth to the jaw is Type 1 (*sensu* Fink, 1891 (73)) characterized by complete ankylosis of the tooth to the jaw-bone, indicating a crushing mode of feeding.

The undetermined jaw fragment is 27 mm long and 11 mm high (Fig 23B). Eight teeth are visible, with two complete ones. They have a very typical acrodine cap at the top, resembling to those of *Scheenstia*. The rest of the teeth consist of dentine and a pulp cavity in the centre.

Fig 23: cf. *Scheenstia* sp. MPV 2022.1.15 and MPV 2022.1.22: Photographs (A1, B1) and drawings (A2, B2) of MPV 2022.1.15 in left lateral view and MPV 2022.1.22 dentary fragment in labial view. **t,** teeth. Scales bars: 1 cm.

Halecomorphi Cope, 1872

Order Amiiformes Hay, 1929

Superfamily Caturoidea Owen, 1860

Material: An Antero-inferior skull part MNHN.F.JRE45 (Mold), Collection Nicollet

Remarks. There is only one mention of halecomorphs at the Vaches Noires Cliffs, that was referred to *Eurycormus grandis* by Wenz (24). Arratia and Schultze (32) revised the genera *Eurycormus* and *Eurypoma*, and referred the first one to the Teleosteomorpha, more specifically the *Siemensichthys*-group, and the second one to the Amiiformes. Based on this study, the specimen described by Wenz (Fig 24), consisting of a lower part of a head, was consecutively

attributed to *Eurypoma grande* (32). Unfortunately, the original specimen seems to have been lost or destroyed. Two molds remains : One in the Houlgate town hall and one is housed at the Muséum d'Histoire Naturelle de Paris (MNHN). Fig 24 shows the replica and a drawing made after (24,32). A study of the caturoids of the Vaches Noires cliffs, based on new material, is in progress.

Fig 24: ***Eurypoma grande* Antero-inferior skull part MNHN.F.JRE45, Collection Nicollet** (Mold from original specimen, the latter being lost), Callovian, Villers-sur-Mer, Calvados, right side (Redrawn and slightly simplified from Wenz 1968, pl. 38, fig. D and Arratia and Schultze, 2007. No major changes). **ang**, angular; **de**, dentary; **io**, infraorbital bone (= lacrimal bone); **met**, mesethmoid; **mx**, maxilla; **na,** nasal bone; **pmx**, premaxilla; **q**, quadrate; **smx**, supramaxilla; **vo.t**, vomerine teeth.

Pycnodontiformes Berg, 1937

Remarks. At the Vaches Noires Cliffs, no complete articulated pycnodont skeleton has ever been found. Two families have been recognized on the basis of isolated elements, the pycnodont incertae sedis *Athrodon* sp. (23), and the Mesturidae with *Mesturus* (52; 24). Fragments of vomers, prearticular, premaxillary and isolated teeth have been regularly found in the Upper Callovian. From this level an almost complete vomer (MPV 2014.2.183), two fragments of vomers, a prearticular (Paleospace display cases) and an endocranium (identification from (24)) from the Collection Nicollet and a dermal tesserae from the Collection Pennetier in Paleospace are studied.

Family incertae sedis

Athrodon sp.

Material: One vomer from Paleospace display cases; Vomer MPV 2014.2.183; One prearticular from Paleospace display cases.

Vomer. The first vomer (Fig 25 A1 and A2) is maximum 19 mm long and is the most complete vomers. There are five rows with six teeth preserved on the Vomer Medial Row (V.M.R.). There are two rows per sides with one V.P.L.R (Vomer Primary Lateral Row) and one V.S.L.R (Vomer Second Lateral Row). The second vomer MPV 2014.2.183 (Fig 25 B1 and B2) is maximum 17 mm long and maximum 10 mm wide. There are five rows and with four teeth preserved on the Vomer Medial Row (V.M.R.). There are three rows on the left side with one V.P.L.R and one V.S.L.R and two rows on the right side with one V.P.L.R and one V.S.L.R.

A fragment of prearticular bearing four large teeth and three small ones (Fig 20 C1 and C2), which are rather similar but less symmetrical than teeth from the vomers is present. We attribute with caution these fragments to *Athrodon* sp. because the teeth are smooth, and uncrenulated unlike *Mesturus*. However, rows are less numerous than in other species of *Athrodon* like *A. wittei* with six lateral rows on each side of the V.M.R. described by Kriwet (23).

Fig 25: *Athrodon* sp. Paleospace display cases: Photographs (up) and drawings (down). Vomer from display cases at Paleospace (A1, A2); MPV 2014.2.183 (B1, B2); prearticular fragment from display cases at Paleospace museum (C1, C2)

Scale bar: 0.5 cm

Mesturidae

***Mesturus* sp.**

Material: Endocranium from Nicollet's collection MNHN.F.JRE46 (Mold) Collection Nicollet; Dermal Tesserae MPV 2022.1.17

Endocranium. A part of an endocranium referred as a large *Mesturus* skull described by Wenz (24) is redrawn here (Fig 26) and a fragment of dermal tesserae figured in Fig 27. The endocranium is a mold from the original specimen described by Wenz (24) which now appear to be lost. The attribution to *Mesturus* is supported by the comparison between the ornamentation of the dermal bones and the dermal tesserae with the squamation pattern observed in *Mesturus cordillera*, which shows a good match (74). Moreover, the specimen falls within the appropriate temporal range for this genus (Callovian to Upper Jurassic) (74).

Fig 26: *Mesturus* sp. MNHN.F.JRE46 endocranium in ventral view. Replica of the original specimen described by Wenz (1967). **Eot**, Epiotic; **Fhm**, Facet for articulation of the Hyomandibular; **Fr**, Frontal; **prpo**, Postorbital process; **PSph**, Parasphenoid; **Ptsph**, Pterosphenoid; **V.n.f**, Vagus nerv foramen; **VII**, Facial nerve foramen. Scale bar: 7 cm.

Fig 27: Mesturus *sp.* MPV 2022.1.17 Fragment of dermal Tesserae. Scale bar: 1 cm.

Teleostei indet.

Material: A batch of 31 vertebrae MPV 2022.1.13 (Pennetier collection); A batch of 33 vertebrae MPV 2014.2.183; A batch of V176R (Charles collection)

Vertebrae. Small amphicoelous vertebrae (concave anteriorly and posteriorly) are regularly found in Vaches Noires Cliffs. Three batches are housed in the Palaeospace collection V176R, MPV 2014.2.183 and MPV 2022.1.13. The first batch V176R contain 47 vertebrae, the second MPV 2014.2.183 contain 33 vertebrae and the third MPV 2022.1.13 contain 31

vertebrae. The centra are well ossified, lack any mid-length constriction, and range in maximum diameter from 2 to 20 mm. Their overall morphology is smooth, with no prominent grooves.

We illustrate the general morphology of the centra using vertebrae from batch 2022.1.13 (Fig. 28). The first centrum is shown in axial view (Fig. 28A1), lateral view (Fig. 28A2), and in either ventral or dorsal view (Fig. 28A3). This type of morphology is typical for centra measuring 5 mm or less, characterized by a very smooth surface.

The other centra (Fig. 28B–E) exhibit variations in shape and coloration and are generally less smooth, although the overall morphology remains consistent. The figured centrum (Fig. 28F), shown in lateral view, is partially broken in the presumed ventral region (lower part of the specimen). However, the upper part clearly displays the general shape seen in most of the specimens.

We acknowledge the possibility that some vertebrae in these batches may belong to chondrichthyans. However, as this paper focuses on bony fishes, we will not discuss them further. One vertebra of particular interest is described in this section. It originates from the V176R batch and measures a maximum of 15 mm in height and 8.5 mm in width. (Fig 29). The bases of the neural arches and parapophyses are preserved, with the latter serving as rib attachment sites. The centrum is marked by deep grooves on both sides.

Only few Middle Jurassic teleosts have strongly ossified vertebral centra as those described here. Among basal teleosts, ichthyodectiforms exhibit such well-ossified centra, which appear squarish in lateral view, resembling the specimens in this study.

Fig 28: Telostei, **representative vertebra centra from batch 2022.1.13 (Collection Pennetier).** Scale bar: 5 mm for A1–A3; 10 mm for B–F.

Fig 29: Teleostei indet. from Bacth V176R: Photograph (A1, B1) and drawing (A2, B2) of the cf. Ichthyodectiformes indet. vertebra from batch V176R. **n.a.b**, neural arch basis; **p**, parapophysis. Scale bar: 5 mm.

V - Diets and trophic web

In this section, we separately analyze each taxon of bony fish recorded in the Callovian assemblage from the Vaches Noires cliffs to provide them with an estimate of their trophic level. This analysis aims to understand the ecological interactions between fish and the diverse fauna of the Callovian Sea. The summary of fishes found in the Vaches Noires cliffs is provided in Table 2, with trophic values and occupied niches presented in Fig 30, and the trophic web reconstruction in Fig 31.

Table 2 : Summary table of fish found in the Vaches Noires cliffs. F: Frequent; LF: Locally frequent; O: Occasional; VR: Very rare.

	Trophic levels	Life habits	Diet	Abundance
Trachymetopon	4,4 (Based on ***Latimeria chalumnae***)	Pelagic?	Piscivorous	LF
Leedsichthys	3,6 (Based on ***Rhincodon typus***)	Pelagic	Filter-feeder	O
Hypsocormus	4,4 (Based on ***Thunnus albacares***)	Pelagic	Piscivorous	O
**Lepidotidae indet.**	4,1 (Based on ***Balistes capristus***)	Nektobenthic	Durophagous	F
Eurypoma	3,8 (Based on ***Amia calva***)	Pelagic	Piscivorous	VR
Mesturus	3,8 (Based on ***Cheilinus trilobatus***)	Nektobenthic	Durophagous	F
**Teleosteans**	3,1 (Based on ***Sardina pilchardus***)	Pelagic	Filter-feeder	F

Coelacanths. All the coelacanths remains from the Vaches Noires have been identified as *Trachymetopon* sp. (8,25). Body size estimation of specimens from the Vaches Noires have been previously made based on the reconstruction of *Axelrodichthys araripensis*, with an estimate length between 4 and 5 meters for large adults (75). All the new material described in this study is small in size, suggesting that these specimens likely represent young individuals.

Extant *Latimeria* is ovoviviparous, meaning the young develop within the mother after hatching. The body size of extant *Latimeria* juveniles born from a 1.8-meter female is approximately 30 to 32 cm (76). By size ratio, juveniles from females 4 to 5 meters long should measure between 60 cm and 75 cm respectively. The estimated sizes of the described individuals correspond to juveniles (25) if not neonates of large *Trachymetopon* individuals reported in the Vaches Noires (8,25,26).

There is little direct evidence of the diet of the extant *Latimeria*, supposed to feed on prey such as fish, small sharks, skates and cephalopods for example (77). We classify *Trachymetopon* as a piscivorous mesopredator.

This animal has a high trophic level, with individuals up to 4-5 meters long likely feeding on small to medium size fishes (sharks, teleosteomorphs, lepidotids, pycnodonts) and cephalopods (ammonites, belemnites) (77). We assign a trophic level of 4.4 identified for *L. chalumnae*, but this value is here considered with caution due to the differences between the living environments and body size of the compared species (although their general morphologies are quite similar).

***Leedsichthys problematicus*.** This giant planktivorous ray-finned fish probably fed on zooplankton and small fishes. It could reach considerable lengths (16.5 metres maximum estimated) (28,56). *L. problematicus* exploited an important resource located at the base of the food chain, the primary consumers. It could probably also feed on small gregarious fish, as fin whales (*Balaenoptera physalus*), humpback whales (*Megaptera novaeangliae*) and whale sharks (*Rhincodon typus*) do today (78–80). We assign for *L. problematicus* a trophic level of 3.6 identified for the living whale shark, *Rhincodon typus* (Orectolobiformes).

Leedsichthys as food source: The Callovian Sea was rich in mesopredators from medium to large-sized (sauropterygians, toothed pachycormids, thalattosuchians). Evidence of attacks

on *L. problematicus* was recorded on dorsal fin rays (NHMUK PV P 6924) and on a caudal fin (NHMUK PV P10000A) (56). Injured bones have formed callus, probably following an attack by the large pliosaur *Liopleurodon ferox* (28). The old or sick individuals of *L. problematicus* were probably to serve as food source to many taxa, such as thalattosuchians and sauropterygians. Scavenging evidence are known from specimen FBS 2012.4.67.30 with stomachal content of a thalattosuchian containing *L. problematicus* gill rakers (35). Evidence of scavenging by *Metriorhynchus* on plesiosaur carcass have been documented, showing that they were opportunistic (81). Moreover, the specimen MPV 2013.1.3 is preserved in association with a *Notidanoides muensteri* tooth, indicating scavenging behaviour from many taxa on *L. problematicus* carcasses on the bottom of the sea. When the conditions were met, carrions ending falling on the bottom of the sea were probably not immediately covered with sediment and were then used as a rich food resource. After cleaning the flesh, the skeleton became an attachment support for various animals, for instance on a piece of a large braincase of *Trachymetopon* found in the Vaches Noires (25) and a food reserve for scavengers (vertebrates or invertebrates) as observed on skeletons of marine reptiles from the Oxford Clay (*Ophtalmosaurus*) (13). These taphonomic observations associated with *L. problematicus* fossils are reminiscent of what we observe today on whales falls (82,83).

cf. *Hypsocormus*. Based on their general morphology and buccal apparatus, these pachycormids likely fed on fishes, cephalopods, and crustaceans (12). Sizes can be estimated by comparison with *Orthocormus* (holotype SenkM 1863) and *Hypsocormus* (holotype SNSB-BSPG AS VI 4a) known by articulated skeletons from the Solnhofen Archipelago (60,65). For MPV 2019.1.1, a standard size ranging between 100 cm and 150 cm is a reasonable estimate. However, since this estimation is based on isolated fragments, it should be considered only

indicative. They are medium-sized predatory fish that consumed small to medium-sized teleosteans, sharks, teuthoids and ammonites. They were probably preyed upon by sharks, marine reptiles and other large predators. We assign a trophic level of 4.4 identified for the living yellowfin tuna, *Thunnus albacares* (Scombriformes).

Lepidotidae. Lepidotidae are widespread in the Callovian of the British Oxford Clays. Martill (84) associated the crushing teeth structure of « *Lepidotes* » *macrocheirus* to five arched incisions present on a damaged peristome of the ammonite *Kosmoceras* cf. *obductum*. This crescent bite-mark also corresponds to the arrangement of the teeth of the specimen MPV 2022.1.15. of Lepidotidae indet. The body size of MPV 2022.1.15. was estimated using ganoid scales MPV 2022.1.16 (Fig 22) combined with the scale/body size ratio used by Cavin et al. (2015) (38) on *Adrianaichthys pankowskii*. The proportionality rule indicates a total length of about 75 cm, a body size in accordance with the size of the quadrate (MPV 2022.1.14, Fig 21).

The jaw fragment (MPV 2022.1.22, Fig 23) bears teeth capped by acrodine caps typical of the genus *Scheenstia*. These fish likely fed on ammonites, benthic crustaceans, and small fish (teleosteans). We suggest that they were essentially consumed by mesopredators and potentially by durophagous hybodontiform sharks, able to crush massive scales and/or thick bones. We do not know where they lived in the water column, but their robust body and massive skull probably indicate a more demersal than pelagic living environment. We assign a trophic level of 4.1 based on the modern gray triggerfish, *Balistes capriscus* (Tetraodontiformes).

Halecomorphs. Halecomorphs are very scarce among the Vaches Noires cliffs. One specimen of *Eurypoma grande* is known from the Collection Charles. Based on the morphology of the jaws (Fig 24), we hypothesize that *Eurypoma* was a generalist predator, feeding on small fishes (teleosts), crustaceans, molluscs, carrions, and other food opportunities. It was likely

preyed upon by mesopredators such as *Cryptoclidus*, thalattosuchians and pliosaurs. We suggest a trophic level of 3.8 based on the extant bowfin, *Amia calva* (Amiiformes).

Pycnodontiformes. Two distinct pycnodonts have been recognized in the Vaches Noires cliffs, the Pycnodontiformes *incertae sedis Athrodon* (23) and the Mesturidae *Mesturus* (24). Kriwet (23) figured an *Athrodon* sp. prearticular, but other occurrences are regularly found in the Callovian of Vaches Noires by fossil collectors. We follow Wenz (1967) (24) who referred a partial braincase to the genus *Mesturus* (Fig 26) and a dermal bone fragment (Fig 27). This genus is restricted to the Callovian and Late Jurassic of Central and Northern Europe (74) and is regularly found in the Oxford Clay. Similar to Lepidotidae, pycnodonts are believed to have lived relatively close to the seafloor, exhibiting a demersal lifestyle. More complete specimens of *Athrodon* and *Mesturus* have demonstrated a generalist way of swimming (85,86), not so closely related to manoeuvring swimming (87,88). As stated by Kriwet (89), we suggest a monotypy of prey, likely gastropods and other molluscs. We assign a trophic level of 3.8 based on the living tripletail wrasse, *Cheilinus trilobatus* (Labriformes).

Teleosteans. Leptolepidae and Pholidophoridae are present in the Peterborough member (13) but there is no conclusive evidence of their occurrence in the Vaches Noires cliffs. However, the abundance of small, smoothly ossified vertebrae in the French assemblage suggests that small fishes, inhabited the Callovian sea of the Vaches Noires. These remains may represent Pholidophoridae or basal Ichthyodectiforms, but current evidence is insufficient to assign them definitively to a specific group. These fishes likely occupied lower trophic levels in the vertebrate food web. They would then have been microcarnivores (suction feeding), or planktonic/zooplanctivores hunting by sight (90,91) and living in schools.

Teleosts and potential basal ichthyodectiforms appear to have been streamlined swimmers, well adapted for prolonged locomotion in open waters (92,93). By a size ratio of vertebrae from V176R (Fig. 29) with the specimen of *Thrissops* cf. *formosus* JME-ETT74 (90) we roughly estimate the total body length of the individual at approximately 60 cm. This size suggests relatively large predator, capable of feeding on many of the inhabitants of the Vaches Noires cliffs' sea. All vertebral centra in the assemblage are relatively large. This may reflect a taphonomic bias favoring the preservation of larger, well-ossified elements, while smaller, more fragile centra are more likely to be destroyed by post-mortem processes. Alternatively, it could result from a collecting bias. We also suspect that finer recovery methods, such as sediment sieving, may reveal the presence of smaller vertebrae that are currently underrepresented. Teleosteans from the Callovian Vaches Noires vertebrate assemblage thus formed the base of the trophic chain but were also already occupying higher trophic levels with predators located in the middle level of the food chain. We assign a trophic level of 3.1 identified for the living sardine, *Sardina pilchardus* (Clupeiformes).

Fig 30 : **Schematic representation of the estimated trophic values (red numbers) and feeding habits (green, blue and orange) of the taxa studied**. 1, *Trachymetopon* sp. ; 2, *Leedsichthys problematicus*; 3, cf. *Hypsocormus* sp. ; 4, Lepidotidae; 5, Amiiformes; 6, cf. *Mesturus*; 7, Teleosteans; 8, Teleostei indet. Scale bar : 1 m.

Fig 31 : **Trophic web reconstruction of the Callovian sea including bony fishes and principals taxa related in the food web (not at scale**). Fishes are in black, other taxa are in grey. Trophic relationships are reconstructed and inferred from bibliography, directs evidence and by actualism. This figure is a simplified model to understand the global ecological implication of the bony fishes fauna among the Callovian sea. 1, Teleosteans ; 2,

Pycnodontiformes ; 3, Lepidotidae ; 4, Chimaeras ; 5, *Leedsichthys* ; 6, Ammonoidea ; 7, Teuthoids ; 8, Teleostei indet. ; 9, Halecomorphs ; 10, *Hypsocormus* ; 11, Hybodonts ; 12, *Trachymetopon* ; 13, *Notidanoides* ; 14, *Cryptoclidus* ; 15, *Ophthalmosaurus* ; 16, *Metriorhynchus* ; 17, *Liopleurodon.*

Fig 32: Ecological « Continuum » of Pelagic Ichthyofauna in Northwestern Europe During the Jurassic (England, Germany France).

VI - Ecological continuity and comparison of the Vaches Noires cliffs during the Middle to Late Jurassic

Here, we compare different localities from the early (Sinemurian and Toarcian), middle (Callovian), and late (Kimmeridgian) Jurassic to illustrate the ecological continuum in the succession of bony fish assemblages (Fig 32). We exclude the sites of Cerin (late Kimmeridgian) and Solnhofen (Tithonian) because they correspond to more lagoonal settings with a deposit much less rich in organic matter. Instead, our objective is to examine Jurassic ecological trends by comparing similar open-pelagic environments characterized by pelletal clays (clays containing small, rounded aggregates known as pellets) and rich shales (fine, laminated structures with 1-5% organic matter) found across Jurassic seas of Western Europe. Toarcian is represented by the assemblage from Holzmaden, the Callovian is represented by the assemblage from the Vaches Noires cliffs and by the assemblage from the English Peterborough Member. Kimmeridgian assemblages are represented by the Kimmeridge Clay. Fig 31 is intended to show the ecological variations between Jurassic fish communities from different periods.

VI.1 - Comparison between the Vaches Noires cliffs and Holzmaden

Holzmaden is a Toarcian Konservat-Lagerstätte located in the Stuttgart district of Germany, within the Posidonia Shale Formation. Unlike the Vaches Noires Cliffs, which corresponds to an environment close to the coast, Holzmaden represents a more open-sea environment. As a result, pycnodonts are almost completely absent from Holzmaden (94). The Holzmaden Toarcian assemblage contains diverse halecomorphs, especially amiiforms (94), and pachycormids, with the macrocarnivorous *Euthynotus*, *Pachycormus*, *Sauropsis*, *Saurostomus*, *Haasichthys* and *Germanostomus* (43,95), and *Ohmdenia* as a basal representant of the filter-feeder lineage (59). In contrast, the Vaches Noires Cliffs exhibit more derived pachycormids, such as *Hypsocormus* sp. and *L. problematicus*. Both deposits have yielded the coelacanth *Trachymetopon* (8,96). The durophagous fish in Holzmaden are represented only by *Dapedium* (97). The palaeonisciform *Coccolepis* is represented in Holzmaden but absent in the Vaches Noires Cliffs (98).

VI.2 – Comparison between the Vaches Noires cliffs and the Peterborough Member (Oxford Clay) (after (12,13))

Although the Peterborough Member and the Vaches Noires Cliffs are both Callovian in age and share similar paleoenvironmental conditions, their fish faunas display notable differences. The Peterborough Member hosts at least one genus of pycnodonts (*Mesturus* and possibly *Gyrodus*), halecomorphs such as *Caturus* and *Osteorachis*, four genera of pachycormids (*Leedsichthys*, *Asthenocormus*, *Hypsocormus*, and *Sauropsis*), the aspidorhynchid *Aspidorhynchus*, and two genera of teleosteans (*Pholidophorus and Leptolepis*). The British locality contains several species that are absent from the Vaches Noires assemblage, including the palaeonisciform *Coccolepis*, the ginglymodian *Lepidotes*, and the dapediid *Heterostrophus*. Interestingly, no coelacanths are mentioned in the Peterborough Member, despite their presence in other localities. In contrast, the Vaches Noires Cliffs has yielded a single halecomorph,

Eurypoma grande (Private coll. Nicolet) (24,32), but *Caturus* is absent, whereas the latter is common in the Peterborough Member (12). Furthermore, the Vaches Noires assemblage includes only two genera of pachycormids (*Leedsichthys* and *Hypsocormus*). Despite these differences, the two localities likely belonged to the same Callovian Sea, and the differences in their fish assemblages are likely due to collecting biases.

VI.3 - Comparison between the Vaches Noires cliffs and the Kimmeridge Clay

The Kimmeridge Clay fish assemblage comprises Holosteans (Lepidotidae, Semionotiformes), Pycnodontiforms with three family: Mesturidae, Gyrodontidae and Pycnodontidae. The pycnodont assemblage illustrates the diversification event among pycnodonts after the Triassic-Jurassic mass extinction (99). Caturidae are represented by *Caturus* and *Strobilodus* (100). We lack sufficient evidence to confirm the presence of halecomorph. Actinistians present in the Kimmeridge Clay Formation are represented by the family Mawsoniidae (*Mawsonia* and/or *Trachymetopon*) (41) and by *Coccoderma*, the latter not being present in the Vaches Noires cliffs.

VII - Discussion The reconstruction of the bony fish assemblage of the Vaches Noires cliffs is reminiscent with the one of the Peterborough Member whose food web was analysed by Martill et al. (1994) [12]. We consider both palaeoecosystems to be very similar. The comparison of the Vaches Noires cliffs with three major Jurassic localities from Western Europe (Holzmaden, Peterborough Member and the Kimmeridge Clays) highlights a diversification after the Triassic-Jurassic mass extinction and a stabilization trend from the Lower (Toarcian) to the Middle Jurassic (Callovian), before the massive Upper Jurassic radiation. While there are some notable species-level differences, the broader ecological landscape remained relatively stable over time from Toarcian (Holzmaden) to Callovian (the

Vaches Noires Cliffs, Peterborough Member). However, the variations in the abundance and diversity of certain genera across the localities (e.g., the absence of coelacanths in the Peterborough Member or the limited representation of halecomorphs in the Vaches Noires Cliffs) may suggest localized adaptive radiation, driven by the availability of ecological niches or variations in environmental conditions. Differences can also be attributed to collecting bias. The comparison between the Vaches Noires Cliffs and the Kimmeridge Clay underscores a higher diversity for the latter, particularly of pycnodonts, and teleosts that represent the incipient Upper Jurassic radiation. Diversification is noticeable in pachycormids, illustrated by the split between the fanged pachycormids and the filter-feeder pachycormids at Toarcian (with *Ohmdenia* from Holzmaden). Their total range starts from the middle of the lower Jurassic (Hettangian with *Euthynotus*) and ends at the terminal Cretaceous (Maastrichtian with *Protosphyraena*) (101,102). This makes them a very successful clade spaning almost 135 Ma. Durophagous (essentially Pycnodontiformes) are present in the Kimmeridge Clay, the Peterborough member and the Vaches Noires cliffs and we notice a diversification among this group of fishes after the upper Lower Jurassic and Middle Jurassic, particularly by the Callovian times (99), with encountered family Mesturidae, Gyrodontidae and Pycnodontidae (33). However, Pycnodontiformes remain extremely rare throughout the Early and Middle Jurassic, particularly before Callovian (103)

There are three, maybe four levels of piscivores in the Vaches Noires Cliffs assemblages: small teleosteans?/*Eurypoma*/*Hypsocormus*/*Trachymetopon* (*Leedsichthys* is not incorporated since its size may prevent any predatory behaviour from other fishes) and three in the Peterborough member : *Caturus*/*Hypsocormus*/*Sauropsis*.

An ecological continuum was established, with only replacement of genera over time (such as for instance the replacement of *Lepidotes* by *Scheenstia*-like fishes after the Toarcian).

Scavenging behaviour on *L. problematicus* highlights the ecological similarities with actual whales falls, that are particularly crucial processes in ocean ecology.

The Vaches Noires cliffs assemblage contributes to our understanding of fish diversification during the Jurassic. The abundance and completeness of fossils are major factors in reconstructing past biodiversity. In this respect the Vaches Noires cliffs provide valuable insights through the quality and quantity of material they have yielded. Some old and recent discoveries by amateurs suggest that fish remains can be exceptionally well-preserved in these marl deposits (an almost complete head of a '*Furo*'-like fish, along with parts of its body, was discovered in 2023 on the rocky platform of the Vaches Noires cliffs. Additionally, a *Leedsichthys* head has been known from a private collection for several years.). One of the most striking features of the Vaches Noires cliffs assemblage is the relative abundance of small coelacanth remains. Recently, a private collector (P. Branger) deposited what we believe to be a juvenile mawsoniid neurocranium from the Callovian, discovered 45 years ago at the Vaches Noires Cliffs. The specimen has been added to the public collections of the University of Rennes and will undergo CT scanning to analyze bone topology and, hopefully, reveal new features to estimate its age, size, and life habits. In parallel, a new specimen tentatively assigned to « *Eurypoma* » *grande* is currently under study. Its remarkable state of preservation make it a key opportunity to refine our understanding of this taxon. Ongoing preparation and analysis may provide crucial data on its anatomy, phylogenetic position, and the role of *Eurypoma grande* within the Callovian marine ecosystem. Additionally, a quantitative analysis of Callovian clay sediments could provide insights into the frequency and diversity of smaller fish remains, such as teeth and vertebrae, allowing for statistical analyses of species representation and paleoecological dynamics.

Acknowledgements:

We thank all the collectors who donated their specimens to French public institutions and shared valuable moments with us during fieldwork: Coraline Bara, Marcelle and Jean Charles, Florian Daumont, Jonas Le Mort, Elisabeth and Gérard Pennetier, and Patrice Rivette. We are also grateful to Jeff Liston for insightful discussions on pachycormid remains, and to Jonas Le Mort for his helpful comments that improved this manuscript.

References

1. Brignon A. Les « ossements pétrifiés » du Jurassique des Vaches Noires dans les cabinets d'histoire naturelle du XVIIIe et du début du XIXe siècles. L'Echo des falaises 2017;21:7-35.

2. Cuvier G. Sur les ossemens fossiles de crocodiles, et particulièrement sur ceux des environs du Havre et de Honfleur, avec des remarques sur les squelettes des sauriens de la Thuringe. Ann Mus Hist Nat. 1808;12:73-110.

3. Brignon A. La collection de vertébrés jurassiques du Calvados de Pierre Tesson. Brignon A (ed.), Bourg-la-Reine. 2018. 82 p.

4. Brignon A. Les premières découvertes de vertébrés jurassiques aux Vaches Noires (Calvados, France). Actes du premier colloque de l'APVSM, « Paléontologie et Archéologie en Normandie », 5-6 octobre 2019. Bulletin de l'Association paléontologique de Villers-sur-Mer. 2019;1:7-40.

5. Eudes-Deslongchamps, E. Les fossiles de la collection de M. Jarry, horloger à Trouville. Bulletin de la Société linnéenne de Normandie 4è série. 1890; 3: 75-81.

6. Eudes-Deslongchamps E. Notes paléontologiques. I-Rapport sur les fossiles oxfordiens de la collection Jarry. Le Blanc-Hardel, Caen. 188980 p.

7. Bardet N, Pennetier E, Pennetier G, Charles A, Charles J. Des os énigmatiques à section triangulaire dans le Jurassique moyen (Callovien) de Normandie. Bulletin Trimestriel de la Société Géologique de Normandie et des Amis du Muséum du Havre. 1993;80(3-4):7-10.

8. Dutel H, Pennetier E, Pennetier G. A giant marine coelacanth from the Jurassic of Normandy, France. Journal of Vertebrate Paleontology. 2014 Jul 29;34(5):1239-1242.

9. Liston JJ. Leedsichthys des Vaches noires...au peigne fin (Normandie, France). L'Echo des falaises. 2008;12:40-50.

10. Dugué O. Géodynamique d'une bordure de massifs anciens la bordure occidentale du bassin anglo-parisien au Callovo-Oxfordien pulsations épigénétiques et cycles eustatiques. Thèse, Caen; 1989.

11. Dugué O, Benabdellouanhed M, Gérard J. Le Jurassique de Normandie : falaises du Bessin et du pays d'Auge. Université de Caen. 2016; 120 p.

12. Martill DM, Taylor MA, Duff KL, Riding JB, Bown PR. The trophic structure of the biota of the Peterborough Member, Oxford Clay Formation (Jurassic), UK. Journal of the Geological Society. 1994 jan;151(1):173-194.

13. Martill DM, Hudson JD, éditeurs. Fossils of the Oxford Clay (Field guides to fossils). London: Palaeontological Association; 1991. 286 p.

14. Dugué O, Fily G, Rioult M. Le Jurassique des cotes Calvados. 1998.

15. Lebrun P, Courville P. Les fossiles des Vaches Noires. Un gisement emblématique du Jurassique à Villers-sur-Mer, Normandie. Fossiles Hors-série. 2013;(HS 4): 16-33.

16. Vullo R. Les poissons du Jurassique des Vaches Noires. 2013;5.

17. Bardet N. Les ichthyosaures et plésiosaures. Fossiles, revue française de paléontologie. 2013;(HS 4): 98-104

18. De Blainville HD. Letter by H.D. de Blainville. In: Eudes-Deslongchamps JA, ed. Lettres sur les crocodiles vivants et fossiles. Bulletin de la Société Linnéenne de Normandie (Années 1849–1853). 1853;9:109-120.

19. Bigot, A. Catalogue des sélaciens jurassiques du Calvados et de l'Orne. Bull. Trim. Soc. Géol. Normandie Amis Mus. Havre. 1896;4(10):7-13.

20. Buffetaut E, Tabouelle J. Thyreophoran vertebrae from the Callovian (Middle Jurassic) of the Vaches Noires cliffs (Normandy, France), with remarks on the dinosaur assemblage from the Vaches Noires. Comptes Rendus Palevol. 2019 Oct;18(7):891-896.

21. Knoll F, Buffetaut É, Bülow M. A theropod braincase from the Jurassic of the Vaches Noires cliffs (Normandy, France) : osteology and palaeoneurology. Bulletin de la société géologique de France. 1999;170(1): 103-109.

22. Monvoisin E, Allain R, Buffetaut E, Picot L. New data on the theropod diversity from the Middle to Late Jurassic of the Vaches Noires cliffs (Normandy, France). Geodiversitas. 2022 March 24;44(12):385-415.

23. Kriwet J. The dentition of the enigmatic pycnodont fish, *Athrodon wittei* (Fricke, 1876) (Neopterygii, Pycnodontiformes; Late Jurassic; NW Germany). Fossil Record. 2008 Aug;11(2):61-66.

24. Wenz S. Compléments à l'étude des poissons actinoptérygiens du jurassique français. Editions du CNRS, Cahiers de Paléontologie. 1967;276 p.

25. Cavin L, Piuz A, Ferrante C, Guinot G. Giant Mesozoic coelacanths (Osteichthyes, Actinistia) reveal high body size disparity decoupled from taxic diversity. Sci Rep. 2021 Dec;11(1): 1-13.

26. Wenz S. Découverte d'un cœlacanthe dans le Jurassique supérieur de Normandie. Bull. Trim. Soc. Géol. Normandie Amis Mus. Havre. 1979;66(4): 91-92.

27. Liston J. The occurrence of the Middle Jurassic pachycormid fish Leedsichthys. 2010;9:36.

28. Liston JJ. *Leedsichthys problematicus* : Arthur Smith Woodward's 'most embarrassing enigma'. Geological Society, London, Special Publications. 2016;430(1):235-59.

29. Liston J. Leedsichthys problematicus : Arthur Smith Woodward's 'most embarrassing enigma'. Geological Society, London, Special Publications. 2016;430(1): 235-259. doi: 10.1144/SP430.8

30. Liston J. A review of the characters of the edentulous pachycormiforms *Leedsichthys*, *Asthenocormus* and *Martillichthys* nov. gen. In: Mesozoic fishes 4. Arratia G, Schultze HP, Wilson MVH (eds.). Verlag Dr. Friedrich Pfeil. 2008. p. 181-198.

31. Liston J, Maltese AE. Hips, tips and sweet sweptback rays: Looking beyond traditional cranial characters in Pachycormiformes [Internet]. PeerJ Preprints; 2017 Aug [2022 Apr 12]. Availaible from: https://peerj.com/preprints/3170v1

32. Arratia G, Schultze HP. *Eurycormus* - *Eurypoma*, two Jurassic actinopterygian genera with mixed identity. Foss Rec. 2007 Feb 1;10(1):17-37. doi: 10.5194/fr-10-17-2007

33. Guinot G, Cavin L. 'Fish' (Actinopterygii and Elasmobranchii) diversification patterns through deep time. Biological Reviews. 2016 Nov;91(4):950-981. doi: 10.1111/brv.12203

34. Jane M. Foraminiferal distribution and sequence stratigraphy of oxfordian successions in the Wessex/Anglo-Paris basin. M. Sc. Thesis, University of Plymouth 2004: 445 p. Available from : http://hdl.handle.net/10026.1/2104

35. Hua S, Liston J, Tabouelle J. The Diet of *Metriorhynchus* (Thalattosuchia, Metriorhynchidae): Additional Discoveries and Paleoecological Implications. Fossil Studies. 2024 Feb 24;2(1):66-76.

36. Pauly D, Palomares ML. Fishing down marine food web: it is far more pervasive than we thought. Bulletin of marine science. 2005;76(2): 197-211.

37. Froese R, Pauly D. Fishbase. 2024. Froese, R. and D. Pauly. Editors. 2024. FishBase. Available on: www.fishbase.org

38. Cavin L, Boudad L, Tong H, Läng E, Tabouelle J, Vullo R. Taxonomic Composition and Trophic Structure of the Continental Bony Fish Assemblage from the Early Late Cretaceous of Southeastern Morocco. Editor Friedman M. PlosOne. 2015;10(5) doi: 10.1371/journal.pone.0125786

39. Cooper SLA, Martill DM. Pycnodont fishes (Actinopterygii, Pycnodontiformes) from the Upper Cretaceous (lower Turonian) Akrabou Formation of Asfla, Morocco. Cretaceous Research. 2020 Dec;116: 1-20. 104607. doi: 10.1016/j.cretres.2020.104607

40. Veiga IMG, Bergqvist LP, Brito PM. Paleoecological inferences on the fish assemblage of the Açu Formation (?Aptian–Cenomanian), Potiguar Basin, NE Brazil: a multiproxy approach. Cretaceous Research. 2023;142: 1-11 105411. doi: 10.1016/j.cretres.2022.105411

41. Toriño P, Gausden SF, Etches S, Rankin K, Marshall JEA, Gostling NJ. An enigmatic large mawsoniid coelacanth (Sarcopterygii, Actinistia) from the Upper Jurassic Kimmeridge Clay Formation of England. Journal of Vertebrate Paleontology. 2022 Jun 30;42(1): 1-11.

42. Dutel H, Herrel A, Clément G, Herbin M. A reevaluation of the anatomy of the jaw-closing system in the extant coelacanth *Latimeria chalumnae*. Naturwissenschaften. 2013 Nov;100(11): 1007-1022. doi: 10.1007/s00114-013-1104-8

43. Cooper SLA, Giles S, Young H, Maxwell EE. A New Large †Pachycormiform (Teleosteomorpha: †Pachycormiformes) from the Lower Jurassic of Germany, with Affinities to the Suspension-Feeding Clade, and Comments on the Gastrointestinal Anatomy of Pachycormid Fishes. Diversity. 24 nov 2022;14(12):1026.

44. Arratia G. The monophyly of Teleostei and stem-group teleosts. Consensus and disagreements*. In: Mesozoic fishes 2. Arratia G, Schultze HP (eds.). Verlag Dr. Friedrich Pfeil. 1999. p. 265-334.

45. Arratia G, Schultze HP, Gouiric-Cavalli S, Quezada-Romegialli claudio. The intriguing †Atacamichthys fish from the Middle Jurassic of Chile an amiiform or a teleosteomorph? In: Ancient Fishes and their Living Relatives: a Tribute to John G. Maisey. Pradel A, Denton SS, Janvier P (eds.). Verlag Dr Friedrich Pfeil. 2021;19-36.

46. Gouiric-Cavalli S, Arratia G. A new †Pachycormiformes (Actinopterygii) from the Upper Jurassic of Gondwana sheds light on the evolutionary history of the group. Journal of Systematic Palaeontology. 29 avr 2022;1-34.

47. Patterson C. Interrelationships of holosteans. In: Interrelationships of fishes. P. H. Greenwood et al. (eds.). 1973. p. 233-305.

48. Hulke JW. Note on some Dinosaurian Remains in the collection of A. Leeds, Esq., of Eyebury, Northamptonshire. By J. W. Hulke. Quarterly Journal of the Geological Society. 1887;43(1-4): 695–702.

49. Martill DM, Frey E, Caceras RP, Chong Diaz G. The giant pachycormid *Leedsichthys* (Actinopterygii) in the Southern Hemisphere: further evidence for a Jurassic Atlanto-Pacific marine faunal province. N. Jb. Geol. Paläont. Mh. 1999 Apr 16;1999(4):243-256.

50. Woodward AS. Preliminary notes on some new little-known British Jurassic fishes. Geol Mag, decade. 1889;3(6):448-455.

51. Cawley JJ, Kriwet J, Klug S, Benton MJ. The stem group teleost Pachycormus (Pachycormiformes: Pachycormidae) from the Upper Lias (Lower Jurassic) of Strawberry Bank, UK. PalZ. juin 2019;93(2):285-302.

52. Friedman M, Shimada K, Martin LD, Everhart MJ, Liston J, Maltese A, et al. 100-Million-Year Dynasty of Giant Planktivorous Bony Fishes in the Mesozoic Seas. Science. 2010 Feb 19;327(5968):990-993.

53. Hüne. Notizen aus dem Woodwardian-Museum in Cambridge. Centralblatt für Mineralogie, Geologie und Paläontologie. 1901: 715-719.

54. Liston J, Gendry D. Le Python de Caen, les algues géantes d'Amblie, et autres spécimens perdus de *Leedsichthys* d'Alexandre Bourienne, Jules Morière, Eugène Eudes-Deslongchamps et Alexandre Bigot. L'écho des falaises. 2015;19: 17-34.

55. Liston J. The plasticity of gill raker characteristics in suspension feeders: Implications for Pachycormiformes. In: Mesozoic Fishes 5 – Global Diversity and Evolution. Arratia G, Schultze HP, Wilson MVH. Verlag Dr. Friedrich Pfeil. 2013. pp 121-143.

56. Liston J, Newbrey MG, Challands TJ, Adams CE. Growth, age and size of the Jurassic pachycormid *Leedsichthys problematicus* (Osteichthyes: Actinopterygii). In: Mesozoic Fishes 5 – Global Diversity and Evolution. Arratia G, Schultze HP, Wilson MVH. Verlag Dr. Friedrich Pfeil. 2013. p. 145-175.

57. Liston JJ. A fish fit for Ozymandias? : The ecology, growth and osteology of Leedsichthys (Pachycormidae, Actinopterygii). [University of Glasgow]: University of Glasgow; 2006.

58. Kear BP. First record of a pachycormid fish (Actinopterygii: Pachycormiformes) from the Lower Cretaceous of Australia. Journal of Vertebrate Paleontology. 12 déc 2007;27(4):1033-1038.

59. Friedman M. Parallel evolutionary trajectories underlie the origin of giant suspension-feeding whales and bony fishes. Proc R Soc B. 2012 March 7;279(1730): pp 944-951.

60. Arratia G, Schultze HP. Outstanding features of a new Late Jurassic pachycormiform fish from the Kimmeridgian of Brunn, Germany and comments on current understanding of pachycormiforms. In: Mesozoic Fishes 5 – Global Diversity and Evolution. Arratia G, Schultze HP, Wilson MVH. Verlag Dr. Friedrich Pfeil. 2013. p. 87-120.

61. Woodward AS. LVIII. — On the affinities of the Cretaceous fish *Protosphyræna*. Annals and Magazine of Natural History. 1894;13(78):510-512.

62. Woodward AS. Catalogue of the fossil fishes in the British museum (natural history). Vol. 3. British museum; 1895. 728 p.

63. Woodward AS. I. — On some remains of the Pycnodont fish, *Mesturus*, discovered by Alfred N. Leeds, Esq., in the Oxford Clay of Peterborough. Annals and Magazine of Natural History. 1896;17(97):1-15.

64. Maxwell EE, Cooper SLA, Serafini G, Schweigert G. A new hypsocormine pachycormid from the Upper Jurassic Nusplingen Plattenkalk (Kimmeridgian, Germany) provides new insights into body plan evolution and scale reduction in Pachycormidae (Actinopterygii). Journal of Vertebrate Paleontology [Internet]. 2025 may 15 [cited 2025 july 15];44(5). doi: 10.1080/02724634.2025.2490123

65. Maxwell E, Lambers P, Lopez-Arbarello A, Schweigert G. Re-evaluation of pachycormid fishes from the Late Jurassic of Southwestern Germany. APP [Internet]. 2020 [cited 2022 june 7];65. doi : https://doi.org/10.4202/app.00749.2020

66. Arratia G, Schultze HP, Wilson MVH. The caudal skeleton of basal teleosts, its conventions, and some of its major evolutionary novelties in a temporal dimension. In:

Mesozoic fishes 5. Arratia G, Schultze HP, Wilson MVH. Verlag Dr. Friedrich Pfeil. 2013. p. 187-246.

67. López-Arbarello A, Wencker LCM. New callipurbeckiid genus (Ginglymodi: Semionotiformes) from the Tithonian (Late Jurassic) of Canjuers, France. Palaöntologische Gesellschaft. 2016;18.

68. Schröder KM, López-Arbarello A, Ebert M. *Macrosemimimus*, gen. nov. (Actinopterygii, Semionotiformes), from the Late Jurassic of Germany, England, and France. Journal of Vertebrate Paleontology. 2012 may;32(3):512-529.

69. Patterson C. Interrelationships of holosteans. In: Interrelationships of fishes. P. H. Greenwood et al. (eds.). 1973. p. 233-305.

70. Thies D. *Lepidotes gloriae*, sp, nov. (Actinopterygii : Semionotiformes) from the Late Jurassic of Cuba. Journal of Vertebrate Paleontology. 1989 March 30;9(1):18-40.

71. López-Arbarello A, Sferco E. Neopterygian phylogeny: the merger assay. R Soc open sci. 2018 March;5(3):172337.

72. Leuzinger L, Cavin L, López-Arbarello A, Billon-Bruyat J. Peculiar tooth renewal in a Jurassic ray-finned fish (Lepisosteiformes, † *Scheenstia* sp.). Editor Smith A. Palaeontology. 2020;63(1): 117-129. doi: 10.1111/pala.12446.

73. Fink WL. Ontogeny and phylogeny of tooth attachment modes in actinopterygian fishes. J Morphol. 1981;167(2): 167-184.

74. Kriwet J. Revision of *Mesturus cordillera* Martill et al., 1998 (Actinopterygii, Pycnodontiformes) from the Oxfordian (Upper Jurassic) of Northern Chile. Journal of Vertebrate Paleontology. 2000;20(3): 450-455. Available from: http://about.jstor.org/terms

75. Maisey JG. Coelacanths from the Lower Cretaceous of Brazil. The American Museum of Natural History. 1986;2866: 32.

76. Itawa M, Yabumoto Y, Saruwatari T, Hukom F. Atmosphere and Ocean Research Institute, The University of Tokyo. NSUGAF. 2011;77(1): 134. doi: 10.2331/suisan.77.134.

77. Fricke, H, Hissmann, K. Feeding ecology and evolutionary survival of the living coelacanth *Latimeria chalumnae*. Marine Biology 2000;136: 379–386. doi: https://doi.org/10.1007/s002270050697

78. Motta PJ, Maslanka M, Hueter RE, Davis RL, de la Parra R, Mulvany SL, et al. Feeding anatomy, filter-feeding rate, and diet of whale sharks *Rhincodon typus* during surface ram filter feeding off the Yucatan Peninsula, Mexico. Zoology. 2010;113(4):199-212. doi: 10.1016/j.zool.2009.12.001.

79. Kawamura A. A review of food of Balaenopterid Whales. Sci. Rep. Whales Res. Inst. 1980;32:155-197.

80. Christensen I, Øien N, Haug T. A review of feeding and reproduction in large baleen whales (Mysticeti) and Sperm whales *Physeter macrocephalus* in Norwegian and adjacent waters. Fauna Norv. 1992 Dec;13: 39-48.

81. Forrest R. Evidence for scavenging by the marine crocodile *Metriorhynchus* on the carcass of a plesiosaur. Proceedings of the Geologists' Association. 2003 Jan;114:363-366. doi: 10.1016/S0016-7878(03)80037-8

82. Gallagher AJ, Papastamatiou YP, Barnett A. Apex predatory sharks and crocodiles simultaneously scavenge a whale carcass. J Ethol. 2018;36(2):205-209. doi: 10.1007/s10164-018-0543-2

83. Quaggiotto MM, Sánchez-Zapata JA, Bailey DM, Payo-Payo A, Navarro J, Brownlow A, et al. Past, present and future of the ecosystem services provided by cetacean carcasses. Ecosystem Services. 2022;54: 1-10. doi: 10.1016/j.ecoser.2022.101406

84. Martill DM. Predation on *Kosmoceras* by semionotid fish in the Middle Jurassic Lower Oxford Clay of England. The Palaeontological Association. 1989;33(3): 739-742.

85. Webb PW. The biology of fish swimming. In: The Mechanics and Physiology of Animal Swimming. Bone Q, M. V. Rayner J, Maddock L, éditeurs. Cambridge University Press; 1994. p. 45-62.

86. Webb PW. Form and Function in Fish Swimming. Scientific American. 1984;251(1): 72-83. Available from: https://www.jstor.org/stable/24969414.

87. Cronin TJ, Shimada K. New anatomical information on the Late Cretaceous bony fish, *Micropycnodon kansasensis* (Actinopterygii: Pycnodontiformes), from the Niobrara Chalk of western Kansas, U.S.A. Transactions of the Kansas Academy of Science (1903). 2019;122(1/2):19-28. doi: 10.1660/062.122.0102

88. Kriwet J. A comprehensive study of the skull and dentition of pycnodont fishes. Zitteliana. 2005;45: 135-188.

89. Kriwet J. Feeding mechanisms and ecology of pycnodont fishes (Neopterygii, Pycnodontiformes). Mitt. Mus. Nat.kd. Berl. Geowiss. Reihe 4. 2001;4: 139-165. doi: 10.1002/mmng.4860040110

90. Ebert M, Kölbl-Ebert M, Lane JA. Fauna and Predator-Prey Relationships of Ettling, an Actinopterygian Fish-Dominated Konservat-Lagerstätte from the Late Jurassic of Southern Germany. Friedman M, Editor. PLoS ONE. 2015;10(1): 1-33. doi: 10.1371/journal.pone.0116140

91. Garrido S, Ben-Hamadou R, Oliveira P, Cunha M, Chícharo M, van der Lingen C. Diet and feeding intensity of sardine *Sardina pilchardus*: correlation with satellite-derived chlorophyll data. Mar Ecol Prog Ser. 2008;354: 245-256. doi: 10.3354/meps07201

92. Cavin L, Forey PL, Giersch S. Osteology of *Eubiodectes libanicus* (Pictet & Humbert) and some other ichthyodectiformes (Teleostei): phylogenetic implications. Journal of Systematic Palaeontology. 2013;11(2):115-177.

93. Ebert M, Kölbl-Ebert M. Morphology of the leading edges of the caudal and other unpaired fins in Late Jurassic Aspidorhynchidae and some other actinopterygian fish in light of their function. 2010;Archaeopteryx(28):1-22.

94. Ebert M, Thies D, Hauff RB. First evidence of ganoin-scaled Halecomorphi (Neopterygii) in the Lower Jurassic of Holzmaden and Ohmden, Germany. njgpa. 2020 march 31 ;295(3):307-326.

95. Delsate D, Duffin C, Weis R (eds.). Les collections paleontologiques du Musee national d'histoire naturelle de Luxembourg. Fossiles du Trias et du Jurassique. Travaux scientifiques du musée national d'histoire naturelle de Luxembourg. 1999;32:1-249.

96. Dutel H, Herbin M, Clément G. First occurrence of a mawsoniid coelacanth in the Early Jurassic of Europe. Journal of Vertebrate Paleontology. 2015;35(3): 1-14. doi: 10.1080/02724634.2014.929581

97. Thies D, Hauff RB. A new species of *Dapedium* Leach, 1822 (Actinopterygii, Neopterygii, Semionotiformes) from the Early Jurassic of South Germany. Palaeodiversity. 2011;4: 185-221.

98. Cooper SLA, López-Arbarello A, Maxwell EE. First occurrence of a †coccolepidid fish (?Chondrostei: †Coccolepididae) from the Upper Lias (Toarcian, Early Jurassic) of southern Germany. Palaeontologia Electronica. 2024;27(1):1-25.

99. Stumpf S, Ansorge J, Pfaff C, Kriwet J. Early Jurassic diversification of pycnodontiform fishes (Actinopterygii, Neopterygii) after the end-Triassic extinction event: evidence from a new genus and species, *Grimmenodon aureum*. Journal of Vertebrate Paleontology. 4 juill 2017;37(4):e1344679.

100. López-Arbarello A, Ebert M. Taxonomic status of the caturid genera (Halecomorphi, Caturidae) and their Late Jurassic species. R Soc open sci. 2023 Jan;10(1):221318.

101. Wretman L, Blom H, Kear BP. Resolution of the Early Jurassic actinopterygian fish *Pachycormus* and a dispersal hypothesis for Pachycormiformes. Journal of Vertebrate Paleontology. 2016;36(5):1-9. doi: 10.1080/02724634.2016.1206022

102. Ferrón HG, Holgado B, Liston JJ, Martínez-Pérez C, Botella H. Assessing metabolic constraints on the maximum body size of actinopterygians : Locomotion energetics of *Leedsichthys problematicus* (Actinopterygii, Pachycormiformes). Palaeontology. sept 2018;61(5):775-783.

103. Cawley JJ, Marramà G, Carnevale G, Villafaña JA, López-Romero FA, Kriwet J. Rise and fall of †Pycnodontiformes: Diversity, competition and extinction of a successful fish clade. Ecology and Evolution. 2021 feb;11(4):1769-1796.

Figure

Click here to access/download;Figure;FIG2_Trachymetopon.tif

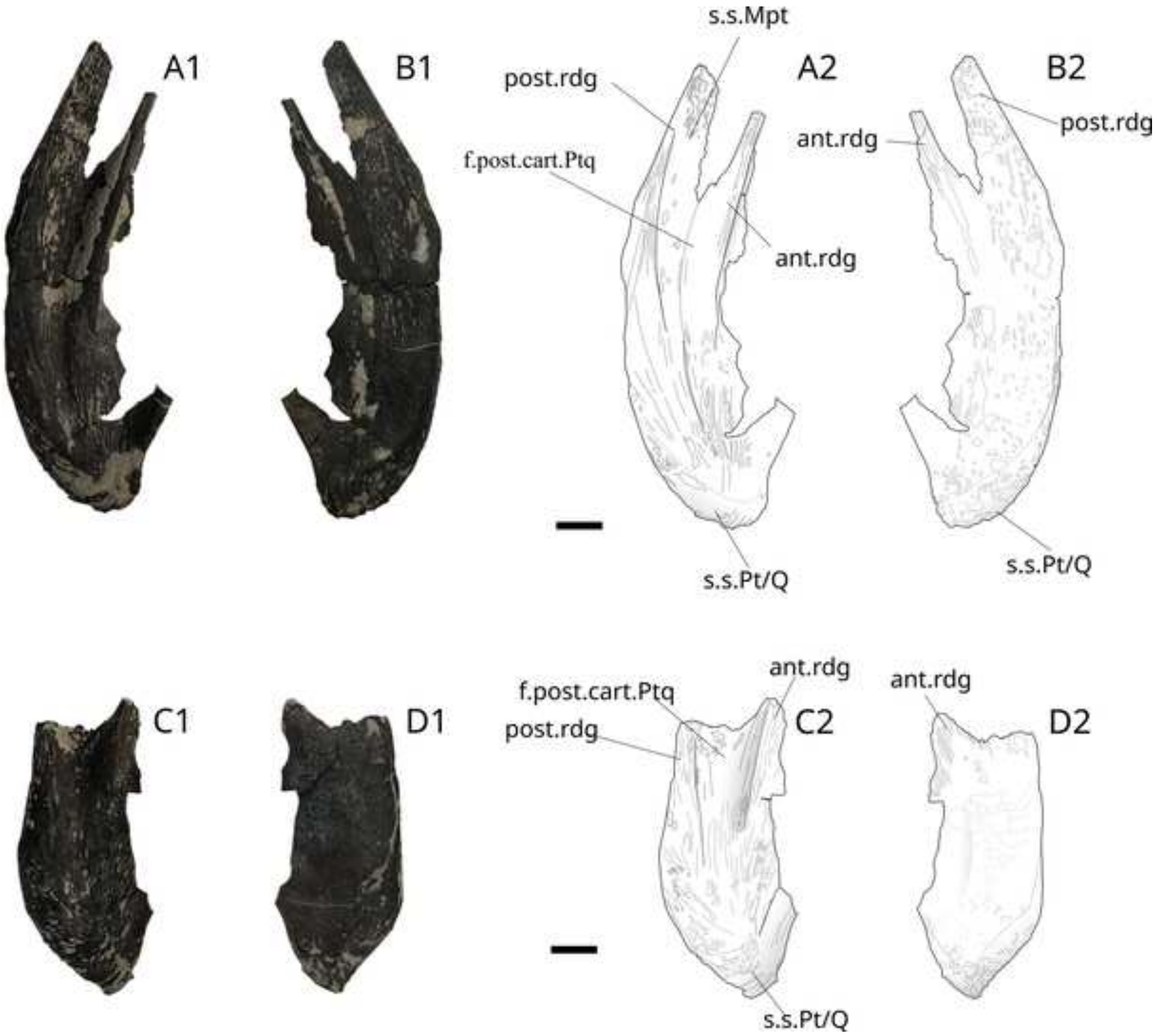

Figure Click here to access/download;Figure;FIG3_Trachymetopon.tif

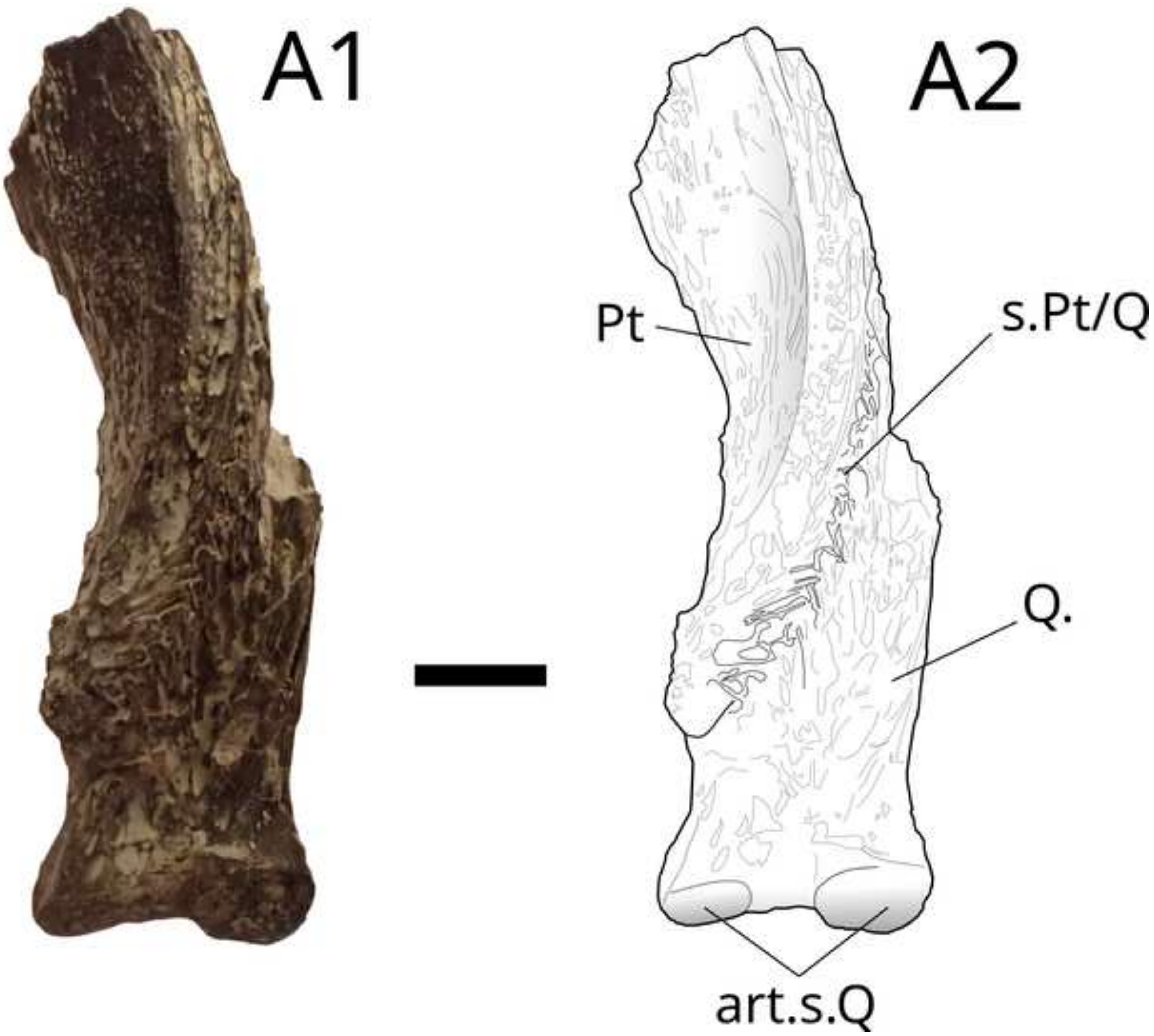

Figure

Click here to access/download;Figure;FIG4_Trachymetopon.tif

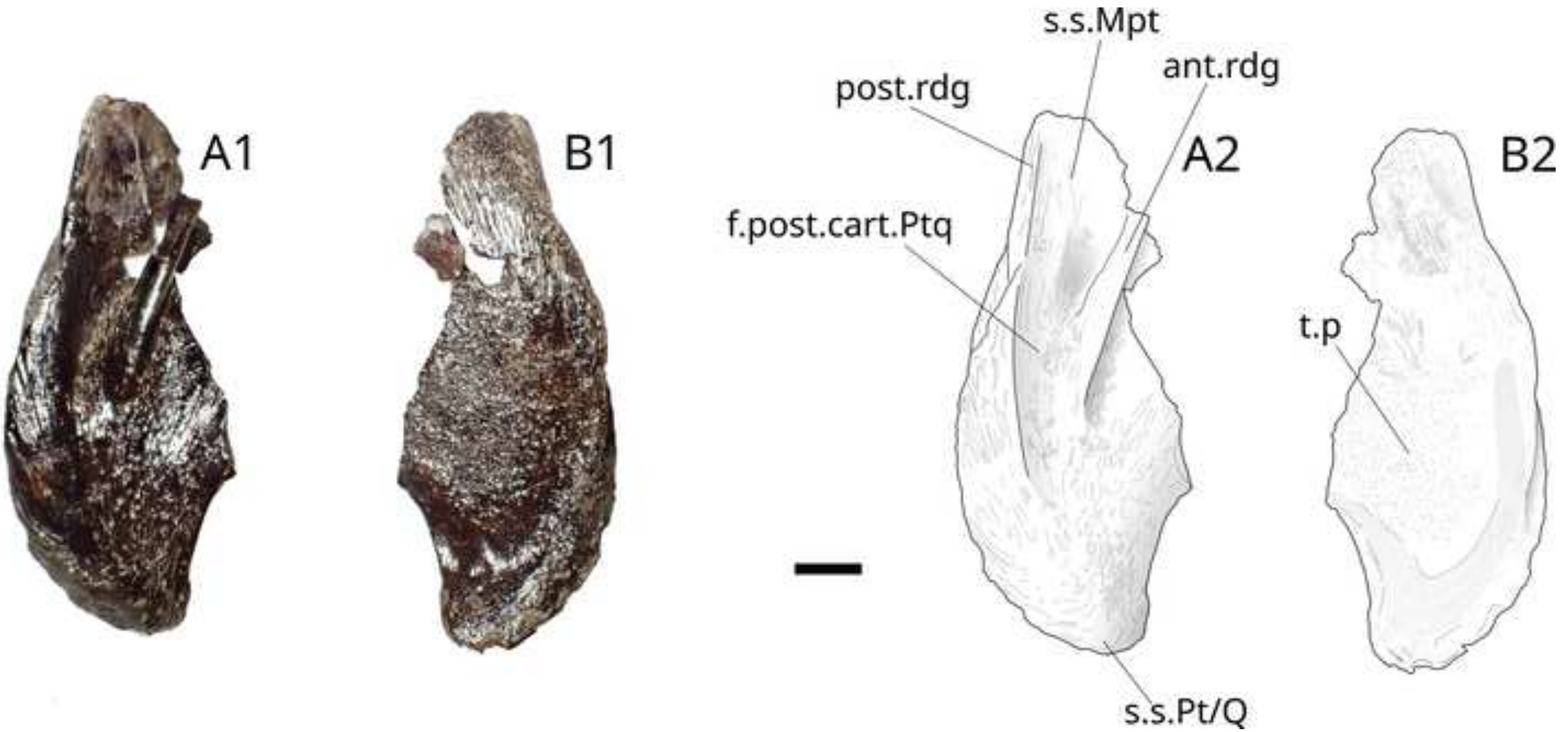

Click here to access/download;Figure;FIG5_Trachymetopon.tif

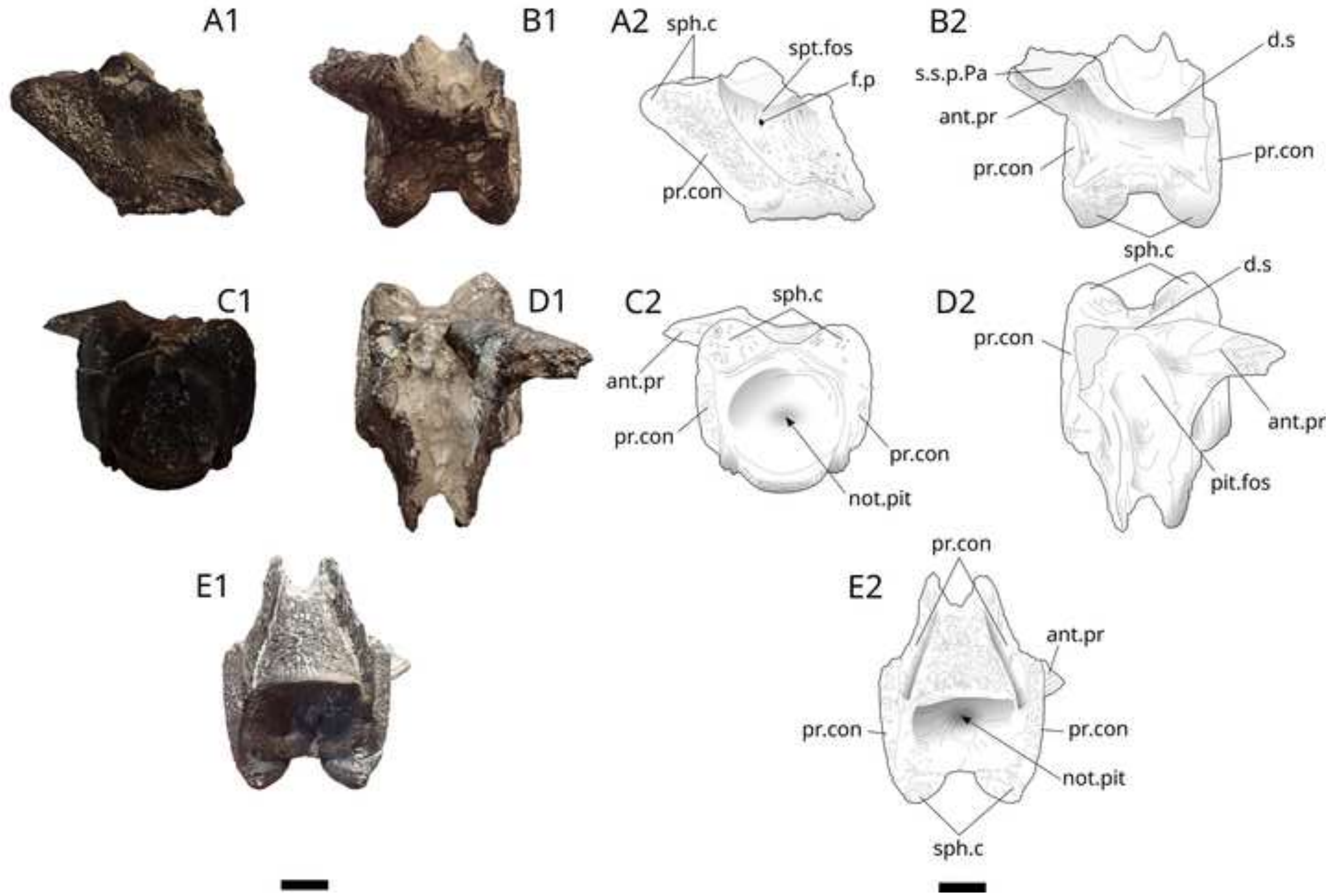

Figure

Click here to access/download;Figure;FIG6_Trachymetopon.tif

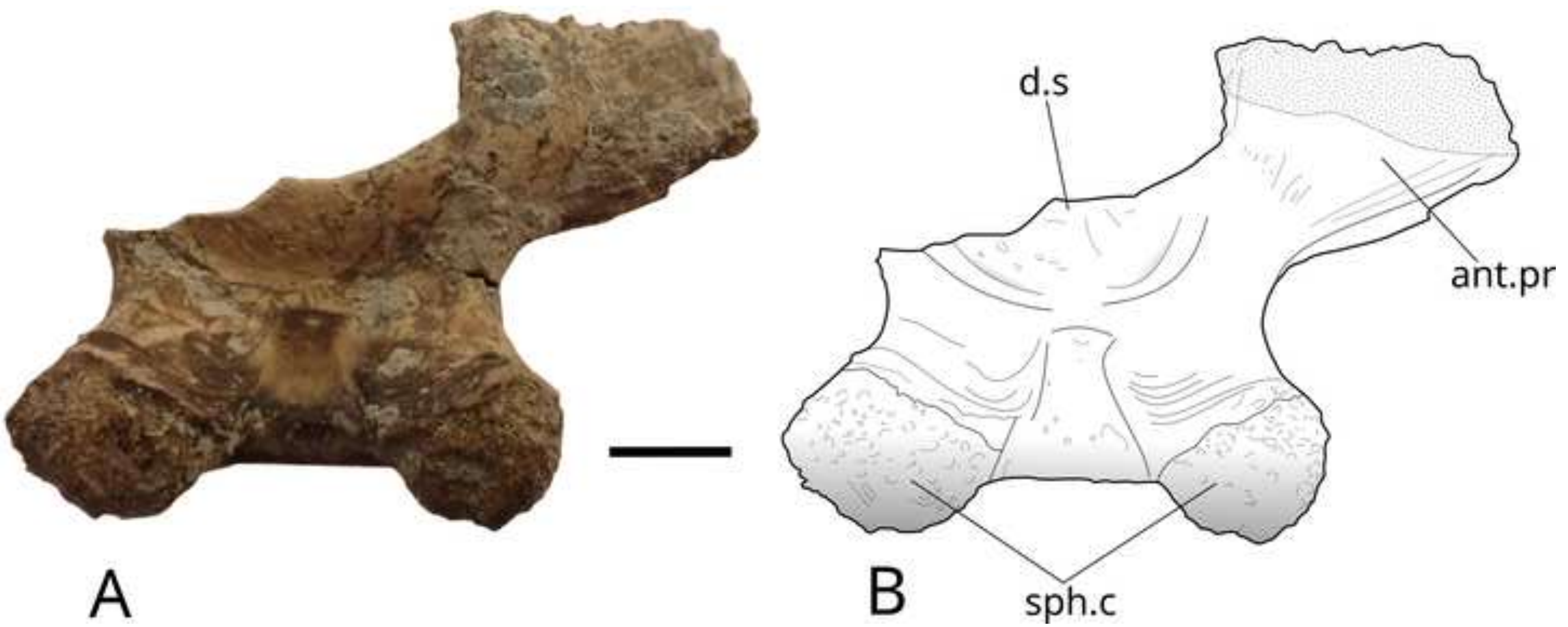

Figure
Click here to access/download;Figure;FIG7_Trachymetopon.tif

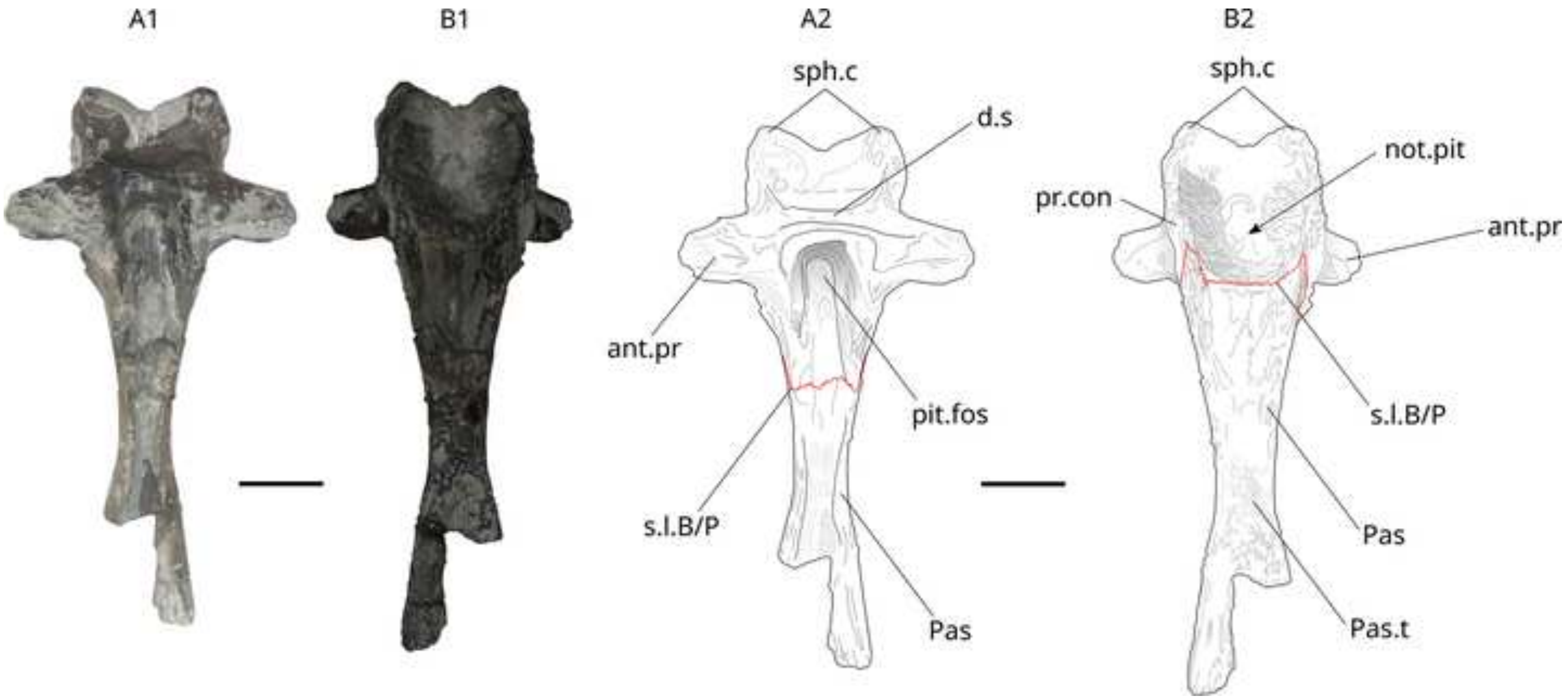

Click here to access/download;Figure;FIG24_Eurypoma.tif

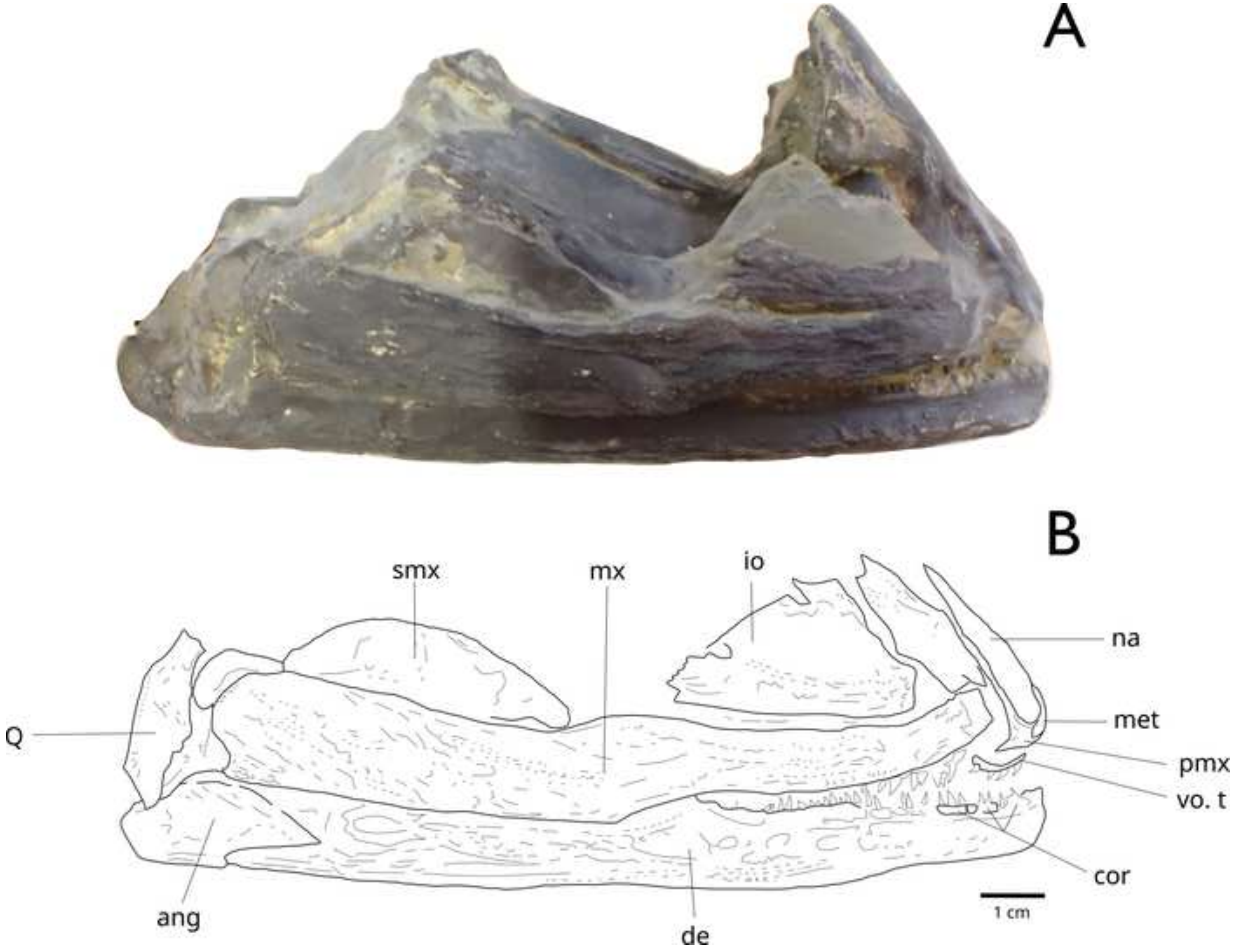

Figure

Click here to access/download;Figure;FIG25_Athrodon.tif

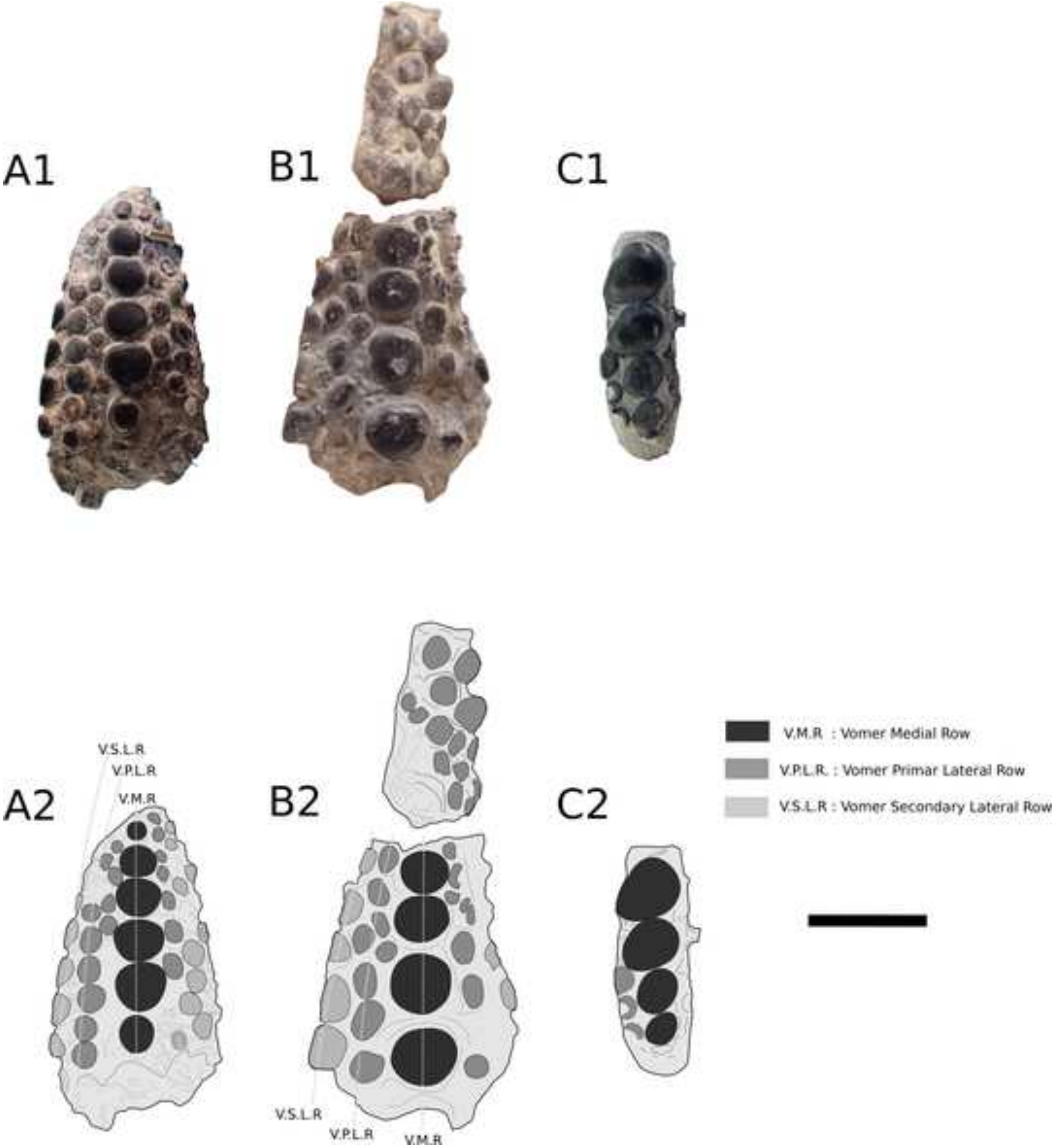

Figure

Click here to access/download;Figure;FIG26_Mesturus.tif

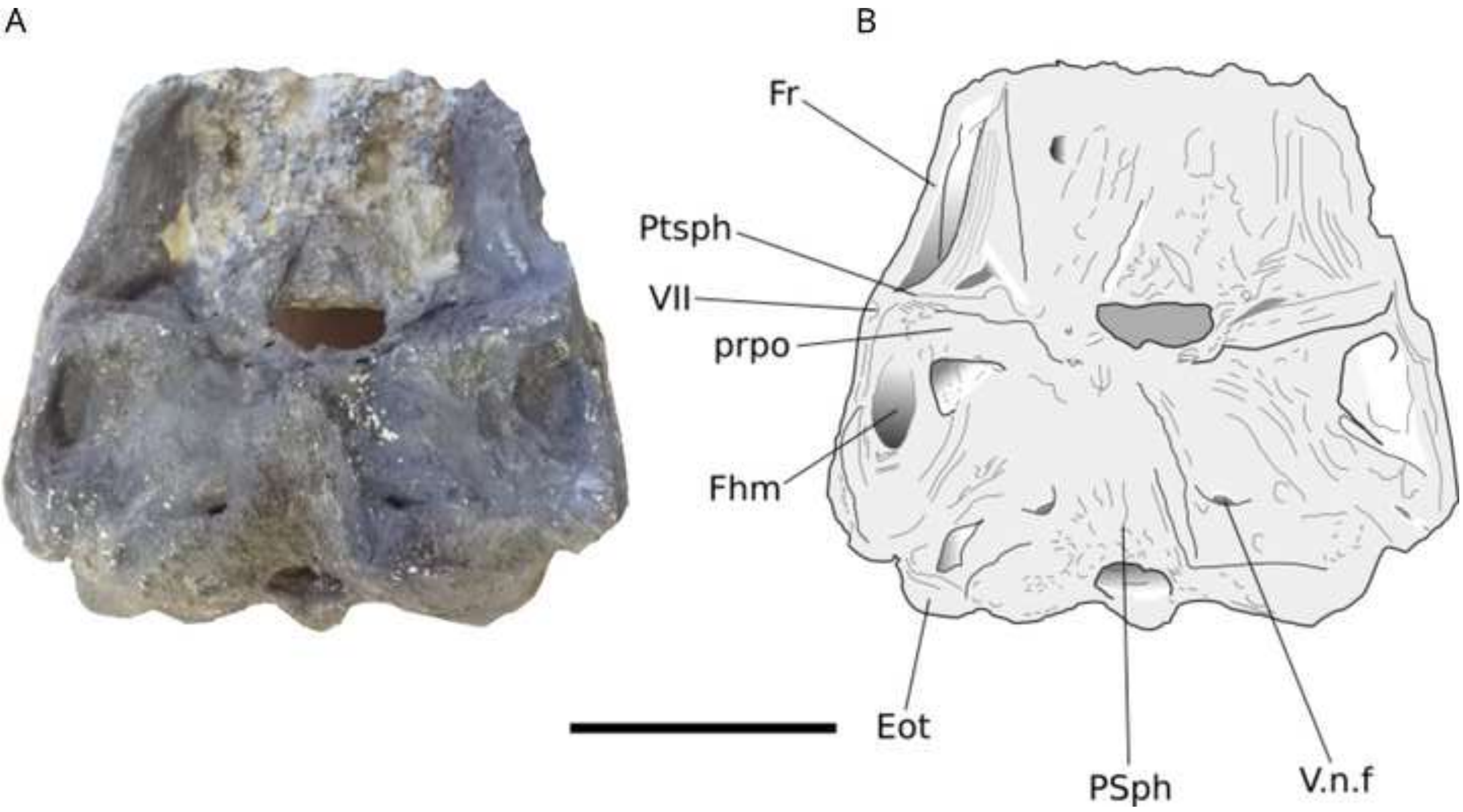

Figure

Click here to access/download;Figure;FIG27_Mesturus.tif

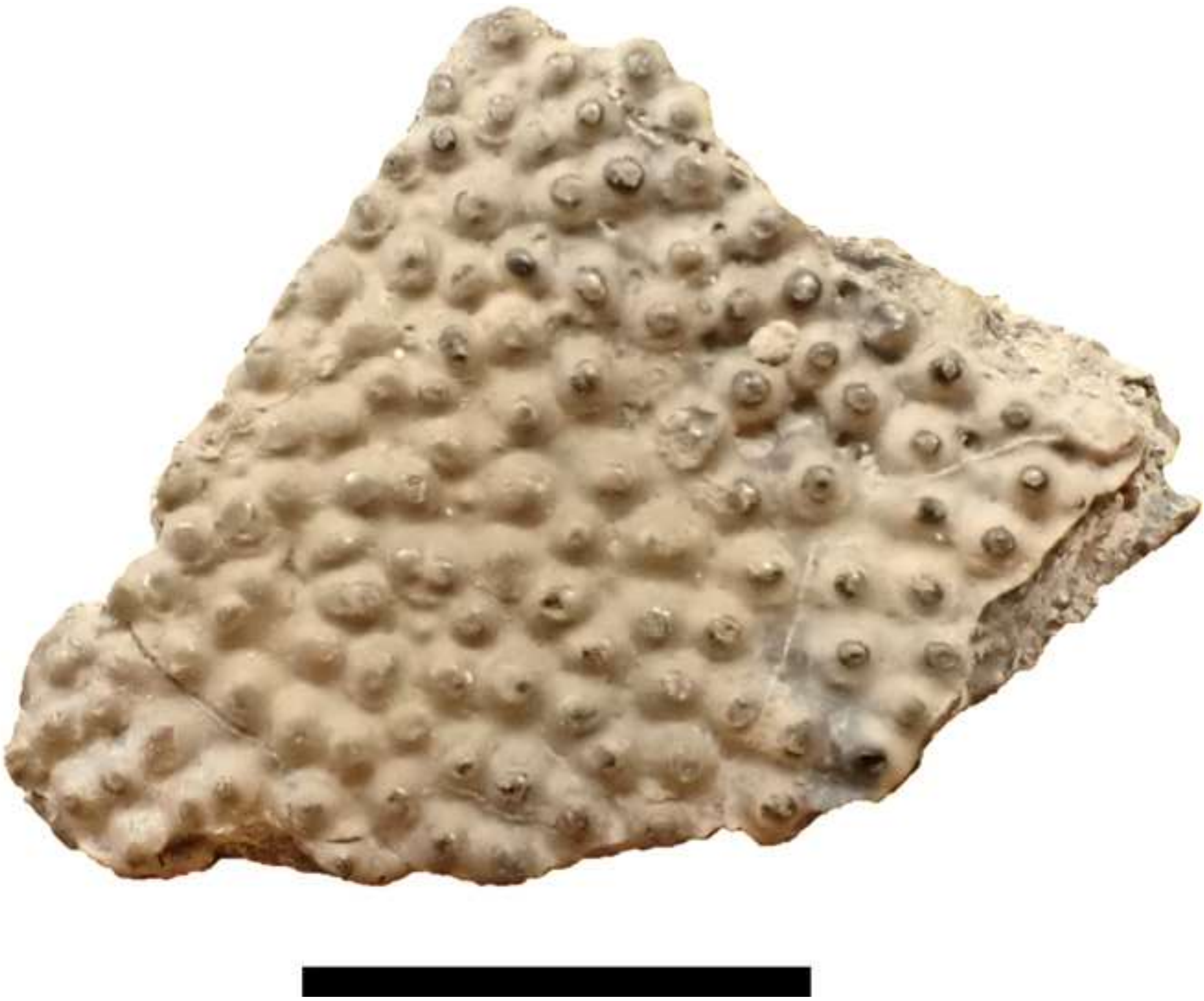

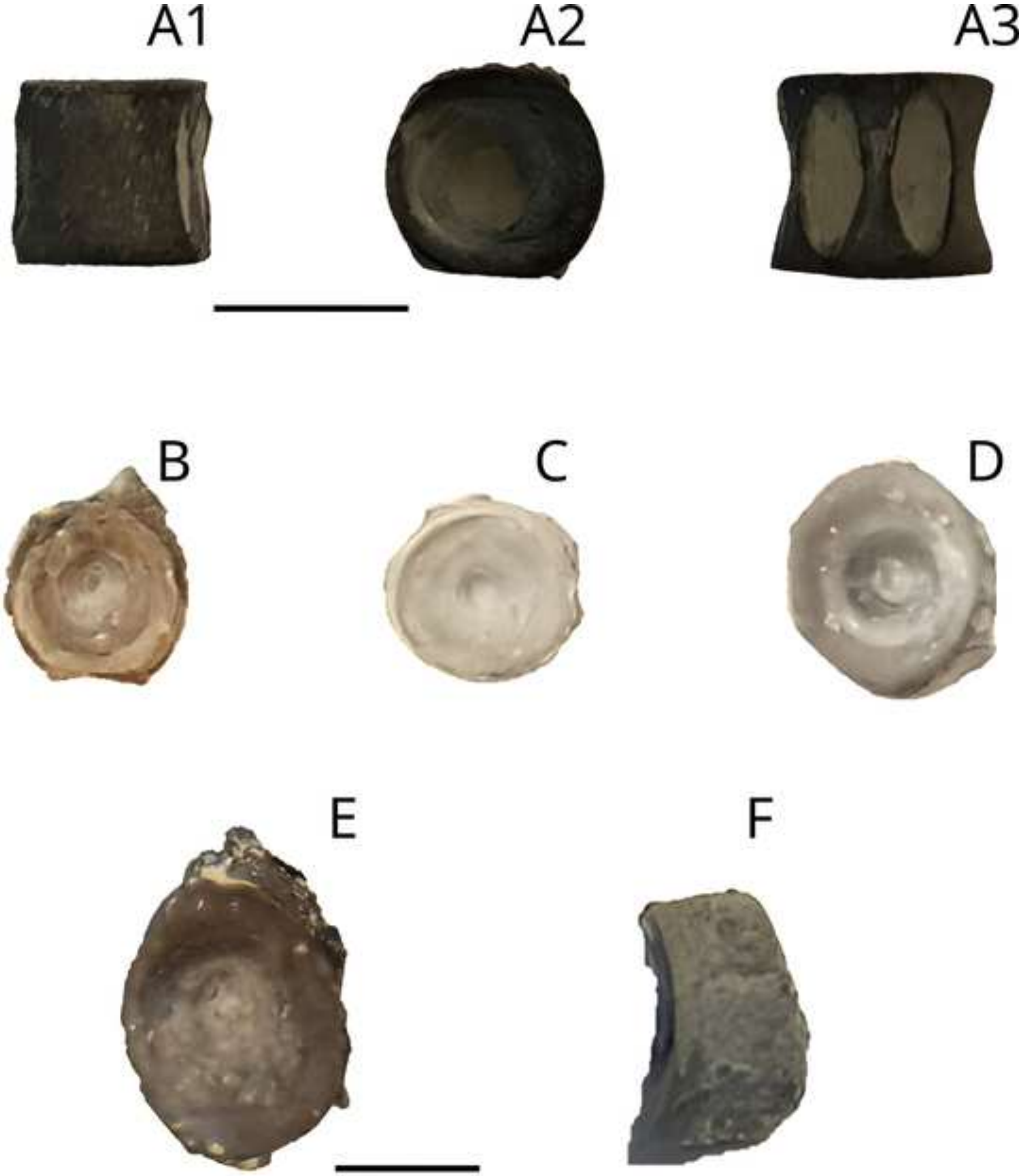
A1
A2
A3
B
C
D
E
F

Figure

Click here to access/download;Figure;FIG29_Teleost.tif

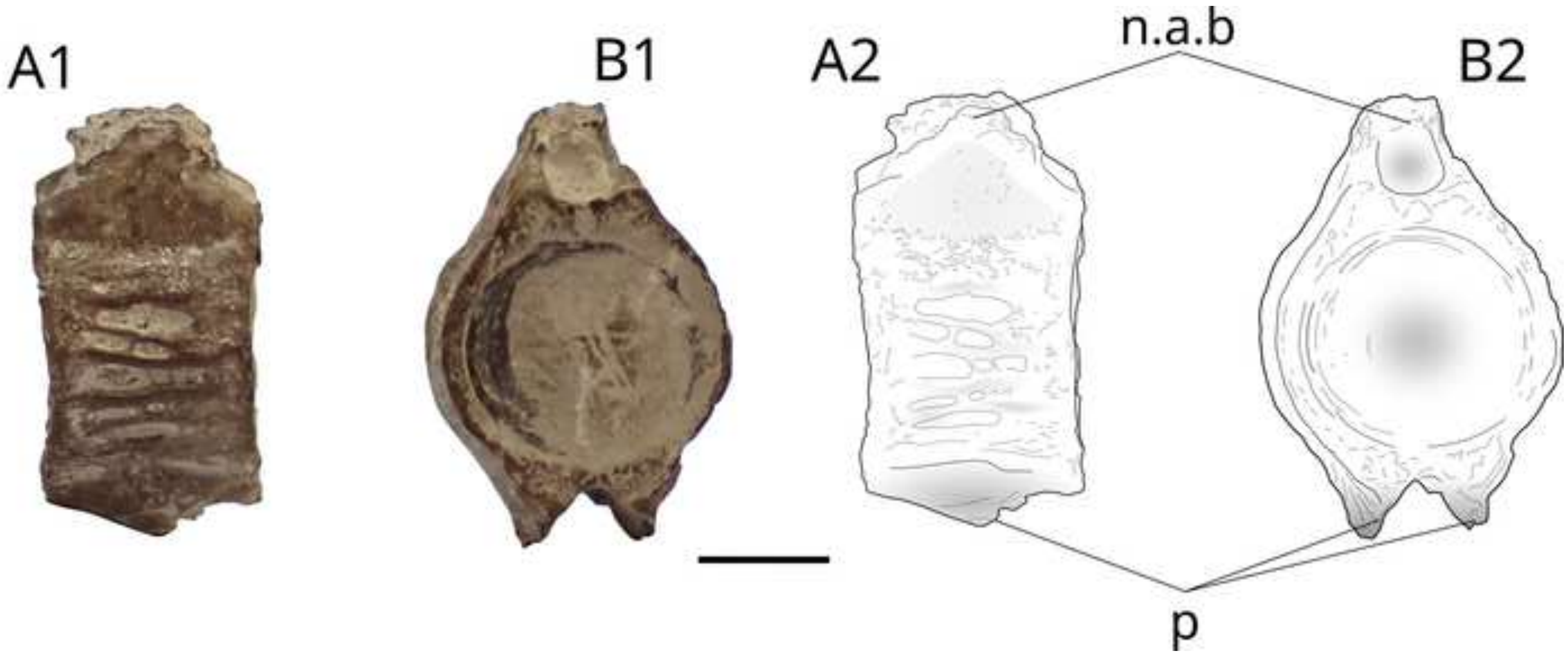

Figure

Click here to access/download;Figure;FIG30_Ichthyofauna.tif

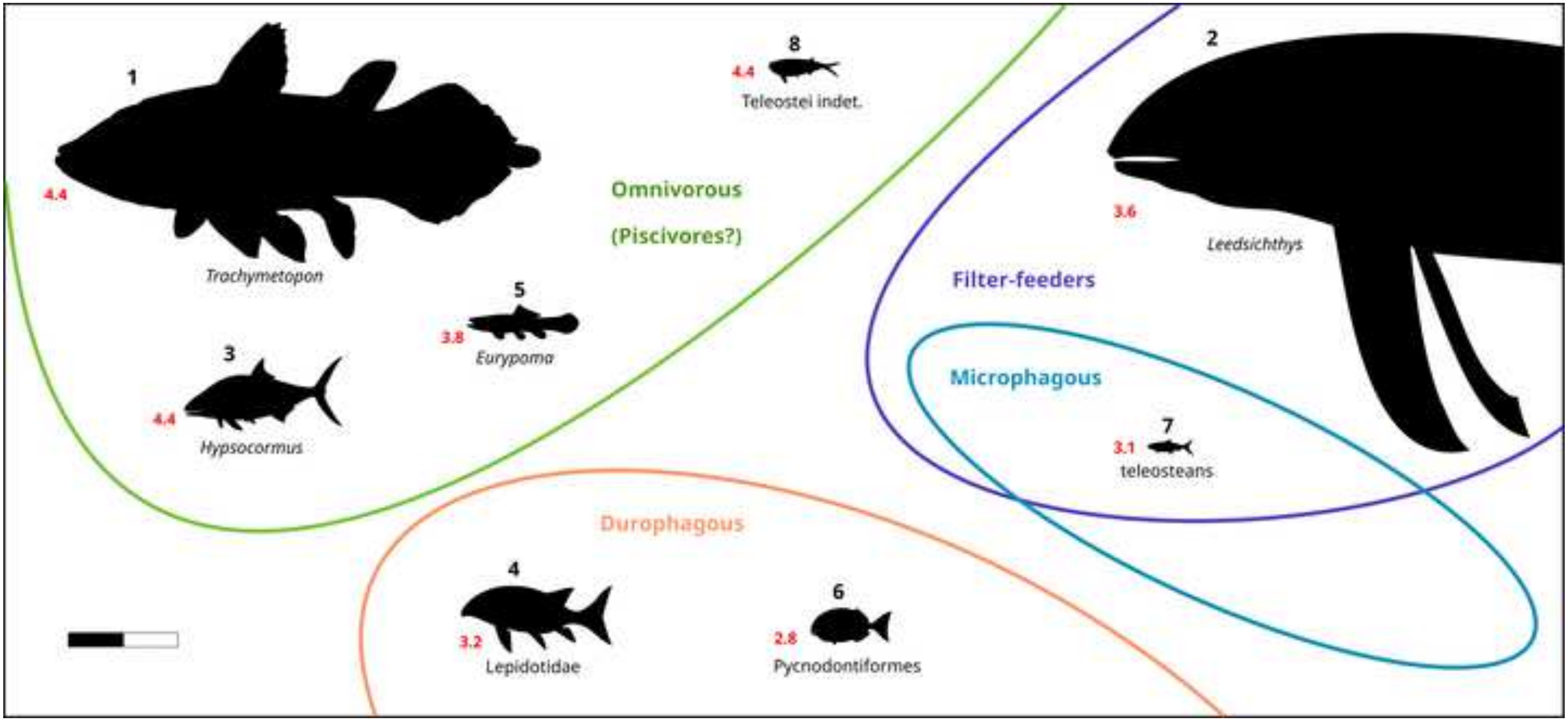

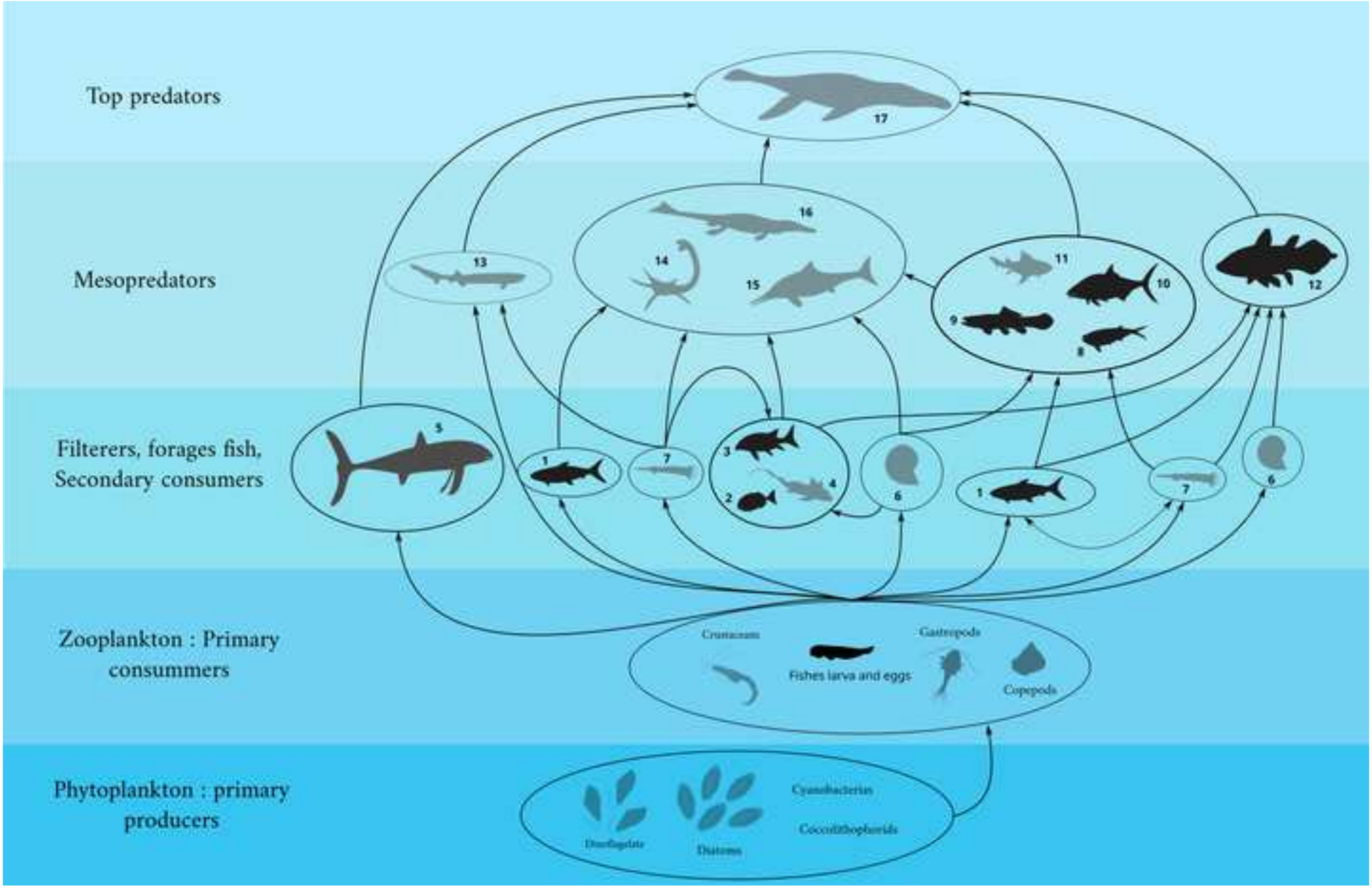
Top predators
Mesopredators
Filterers, forages fish,
Secondary consumers
Zooplankton : Primary
consummers
Phytoplankton : primary
producers
Fishes larva and eggs
Gastropods
Copepods
Cyanobacteria
Coccolithophorids
Diatoms

Figure
Click here to access/download;Figure;FIG32_Ecological_Continuity.tif

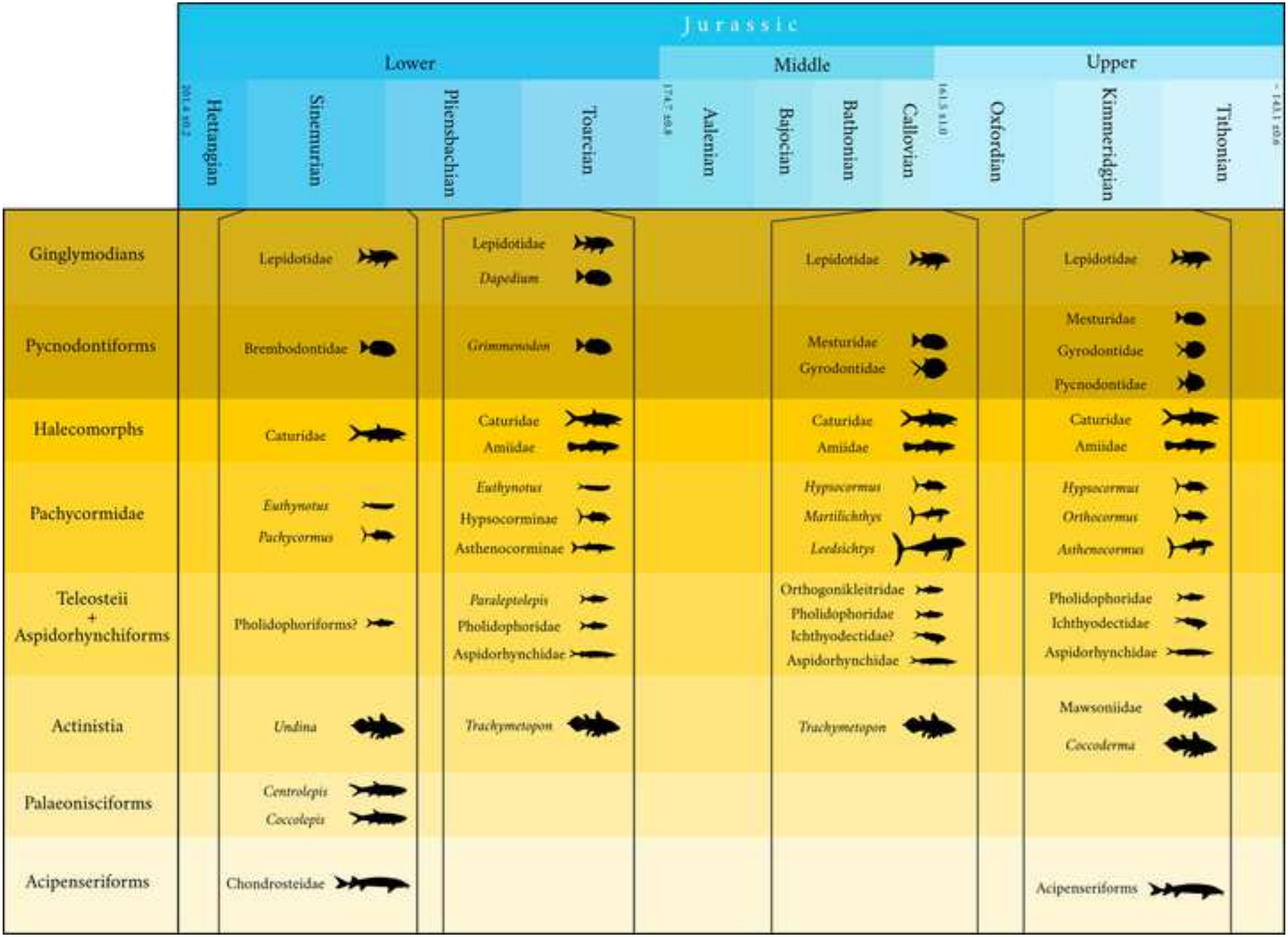

 Click here to access/download;Figure;FIG8_Leedsichthys.tif

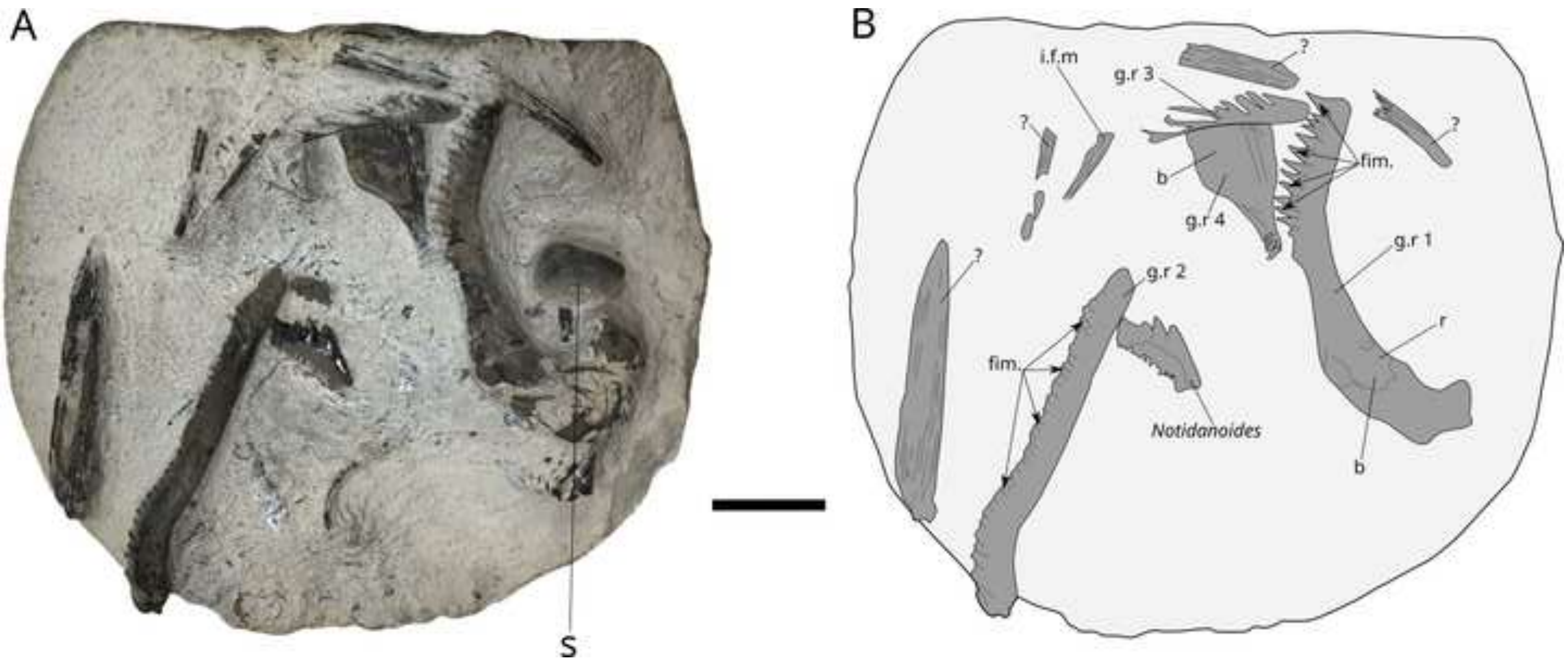

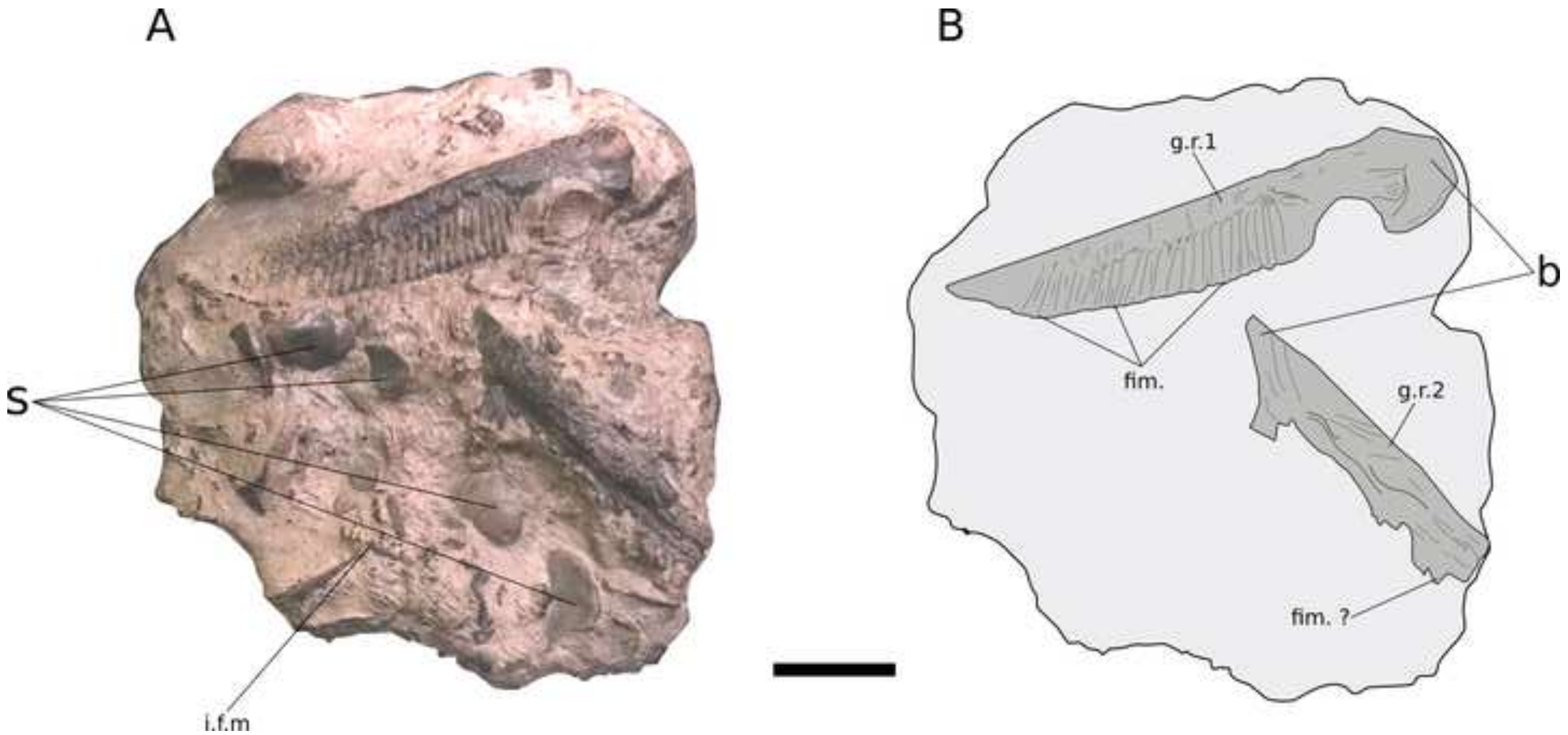
A
B
S
i.f.m
g.r.1
b
fim.
g.r.2
fim. ?

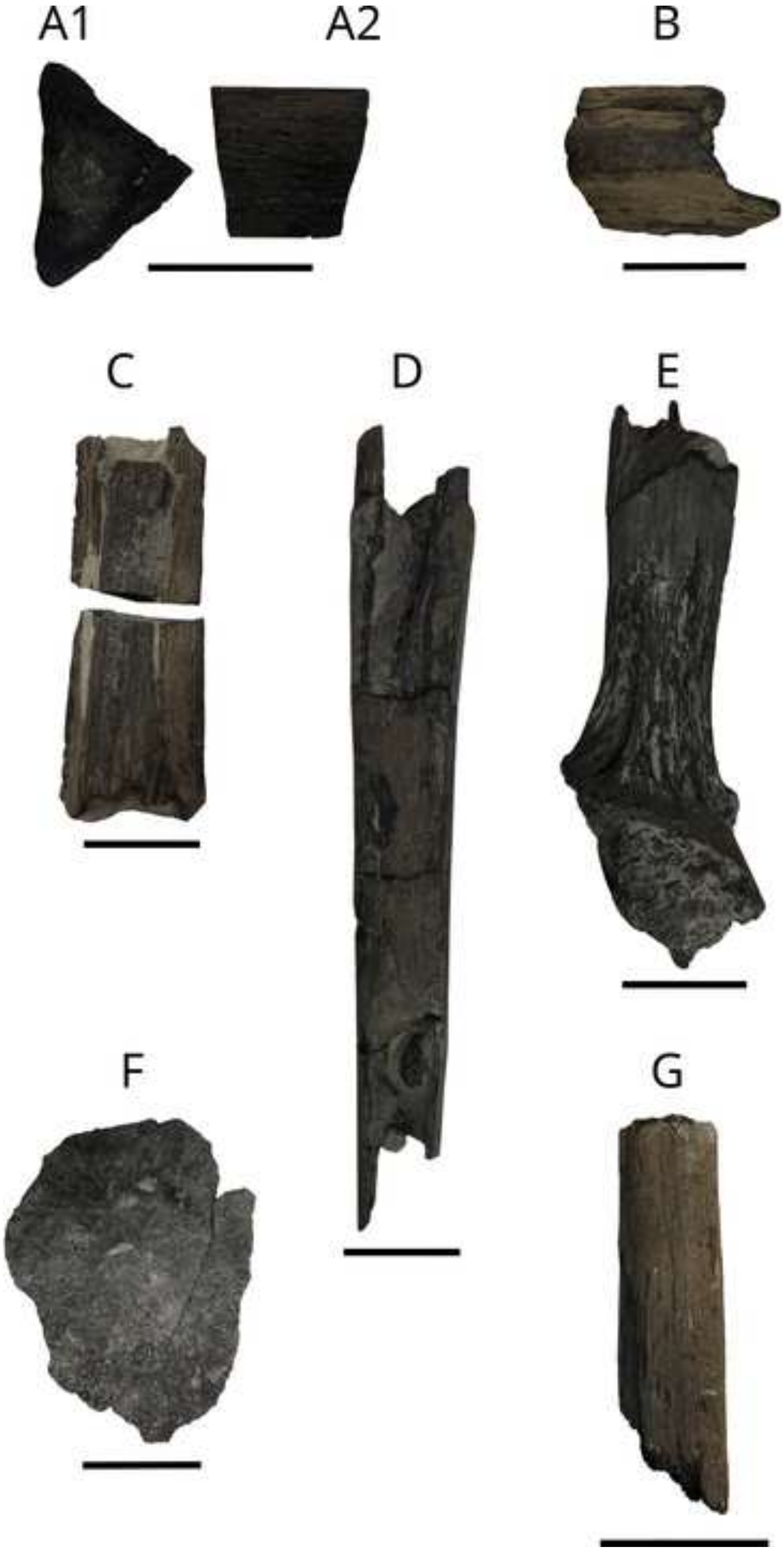
A1
A2
B
C
D
E
F
G

 Click here to access/download;Figure;FIG11_Leedsichthys.tif

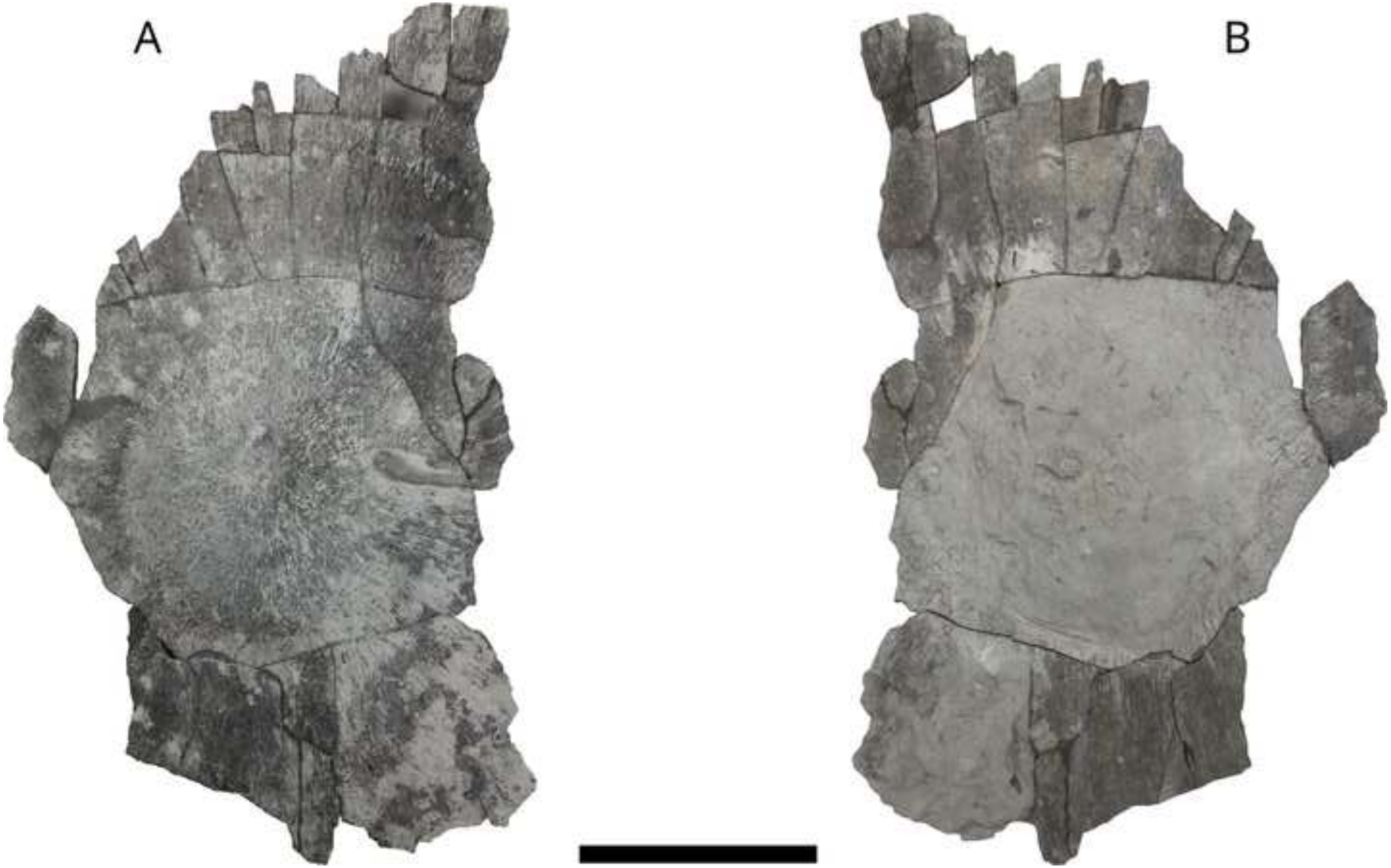

Click here to access/download;Figure;FIG18_Hypsocormus.tif

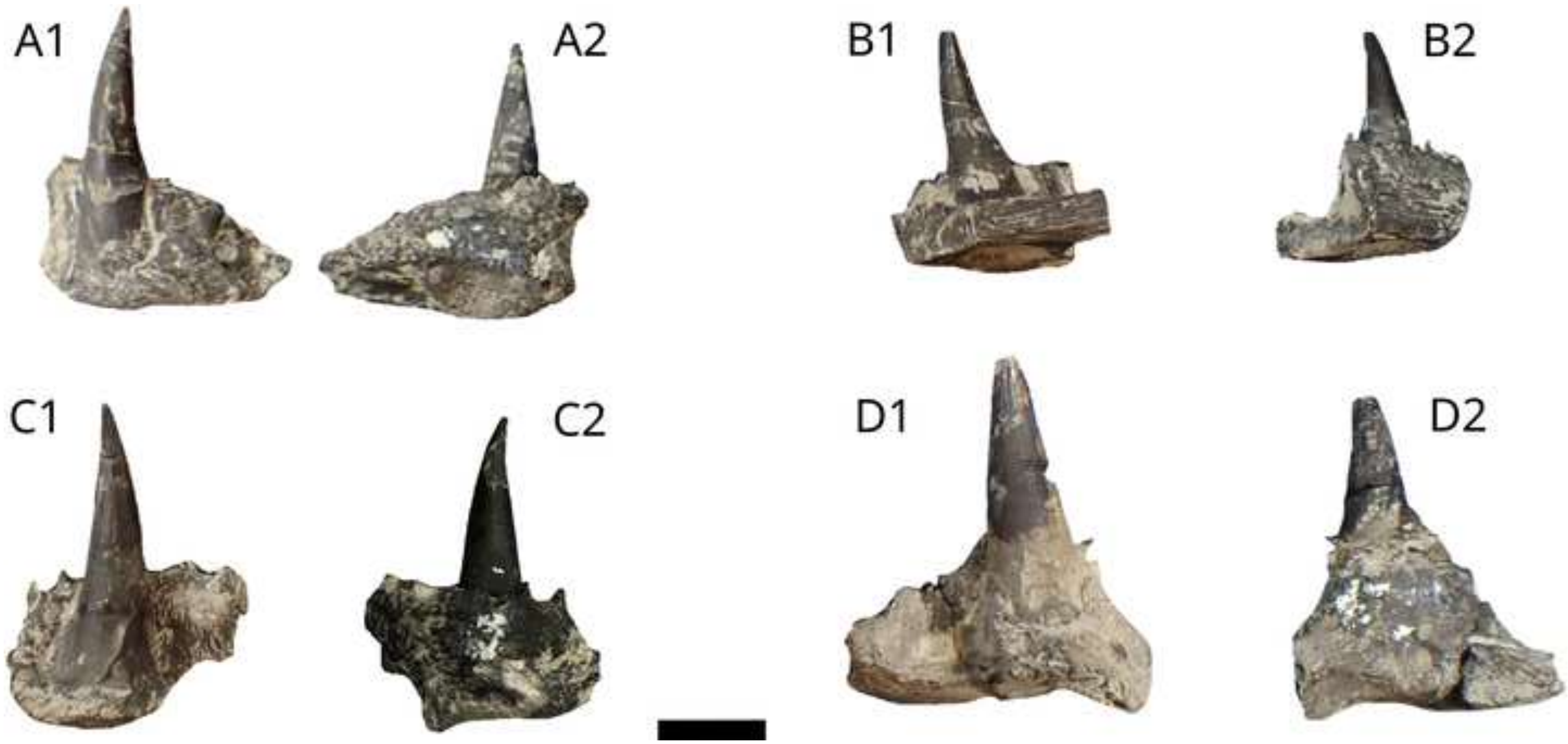

 Click here to access/download;Figure;FIG19_Hypsocormus.tif

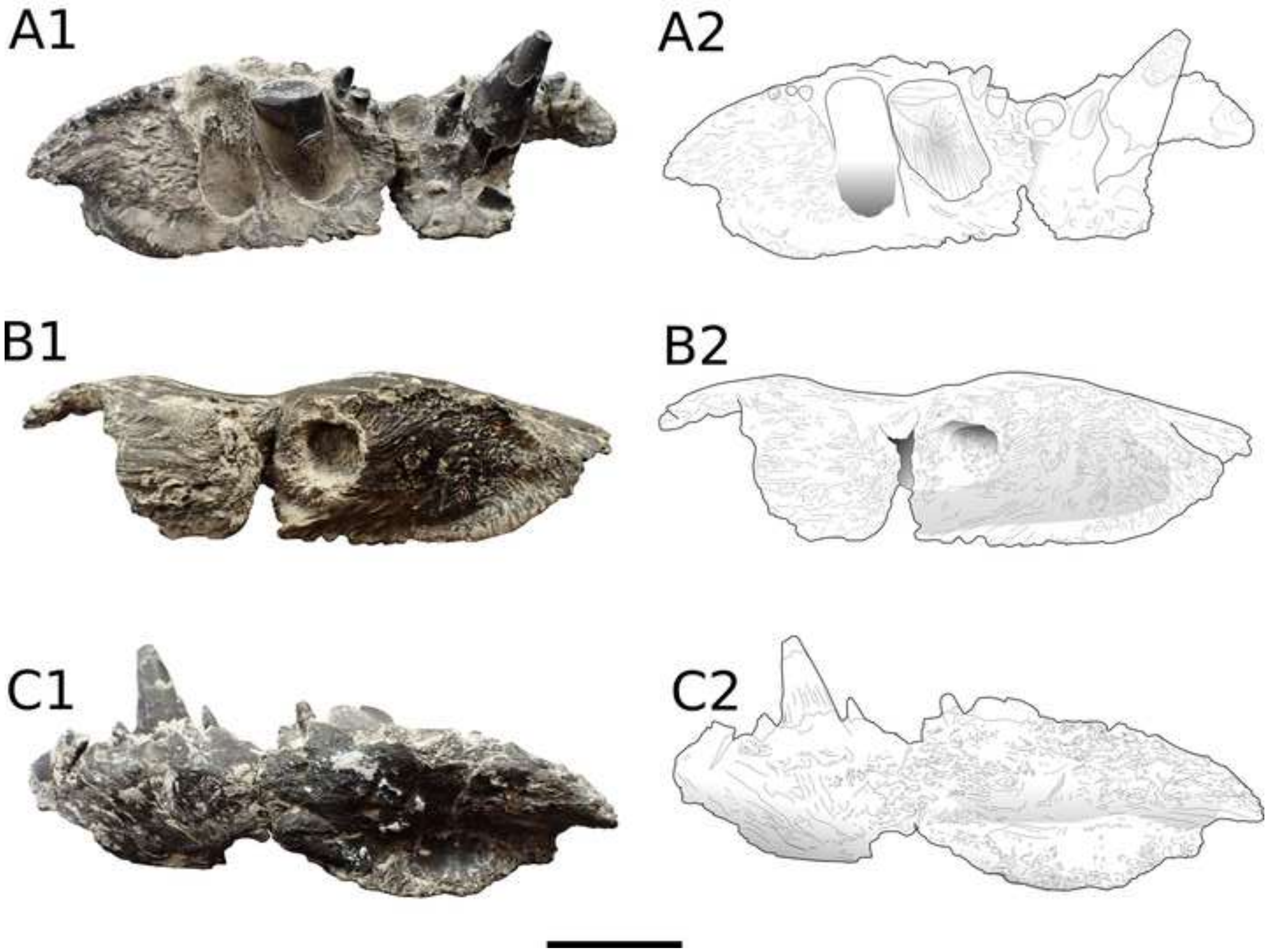

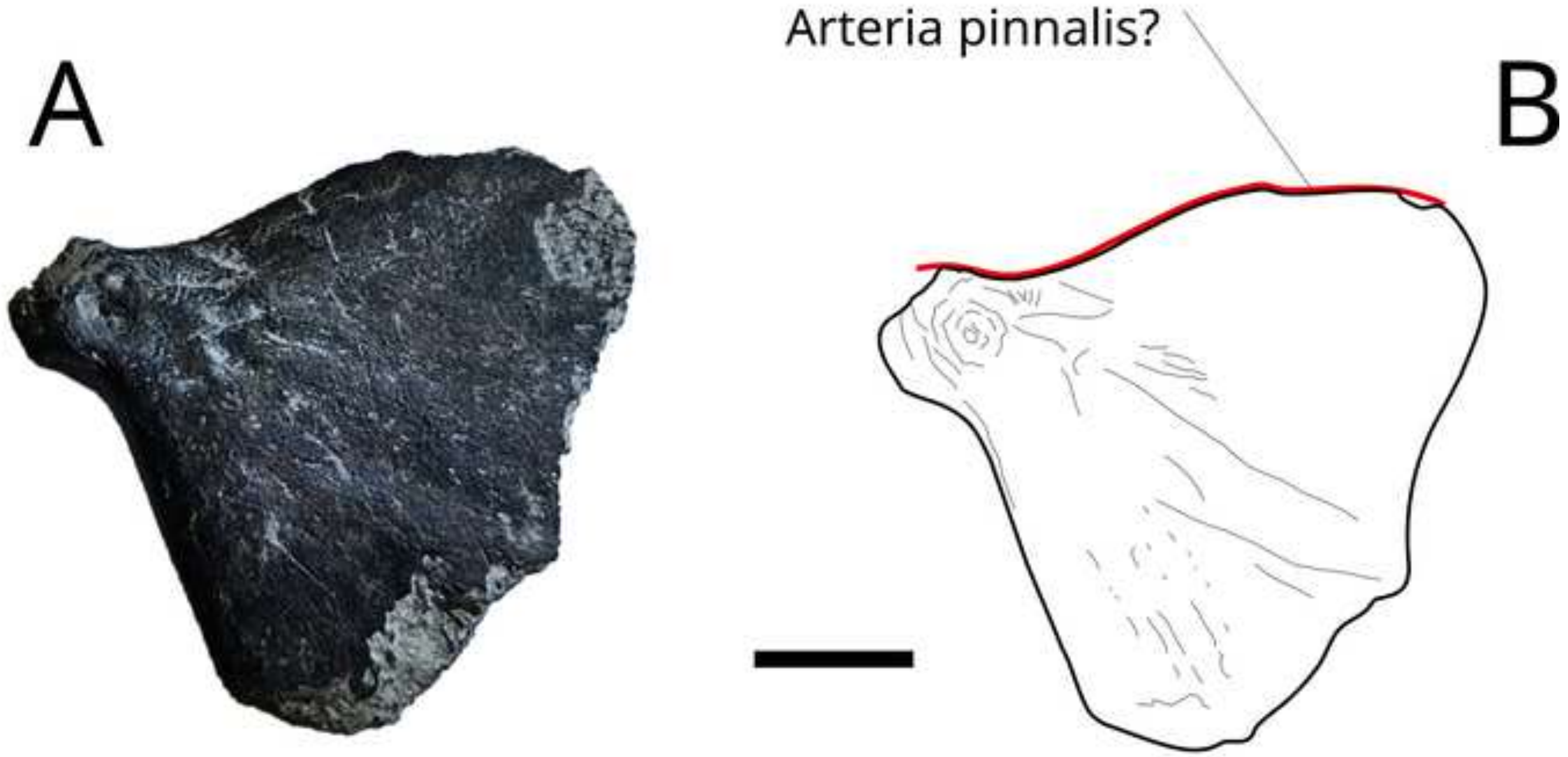
A
Arteria pinnalis?
B

Figure

Click here to access/download;Figure;FIG21_Lepidotidae.tif

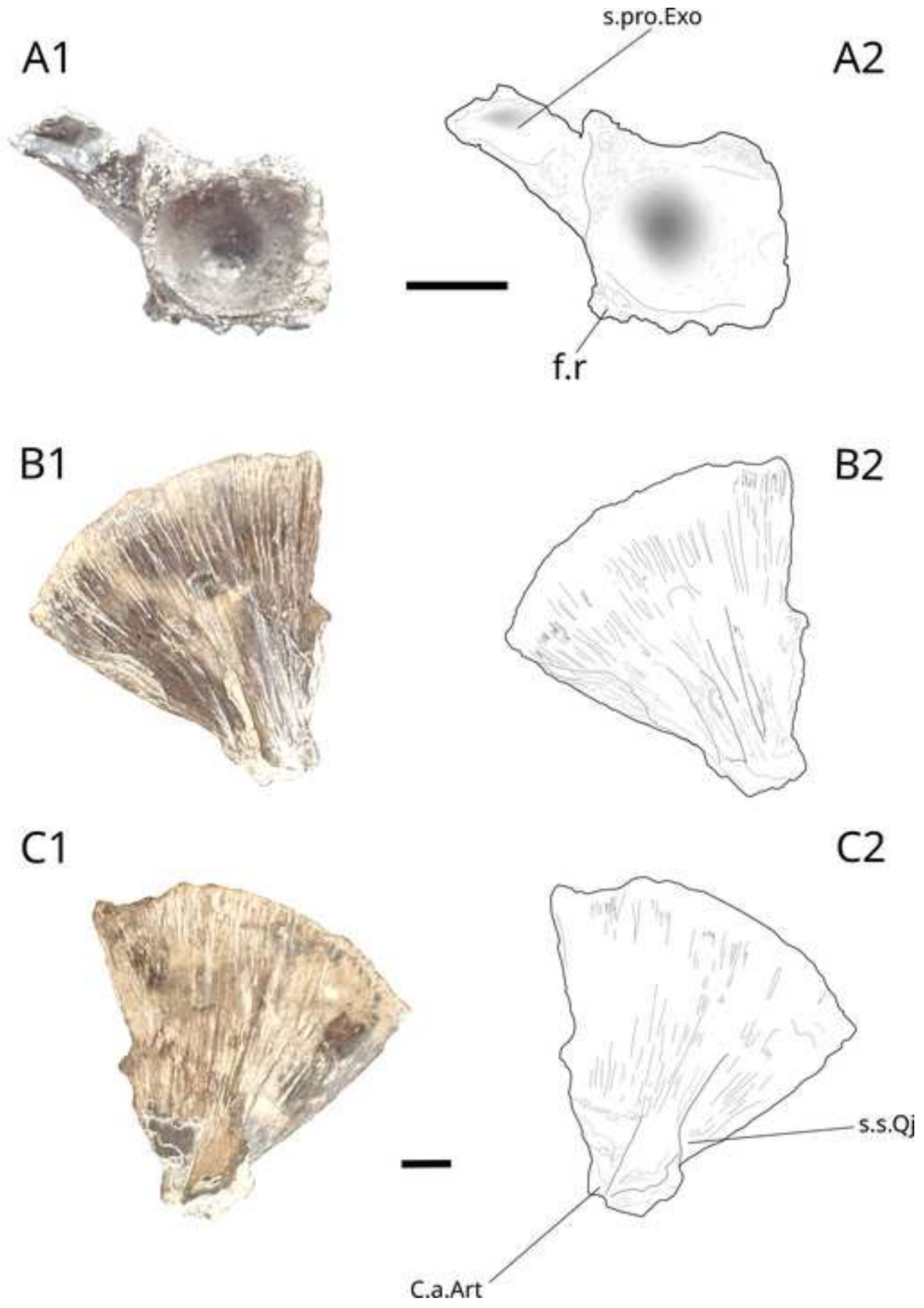

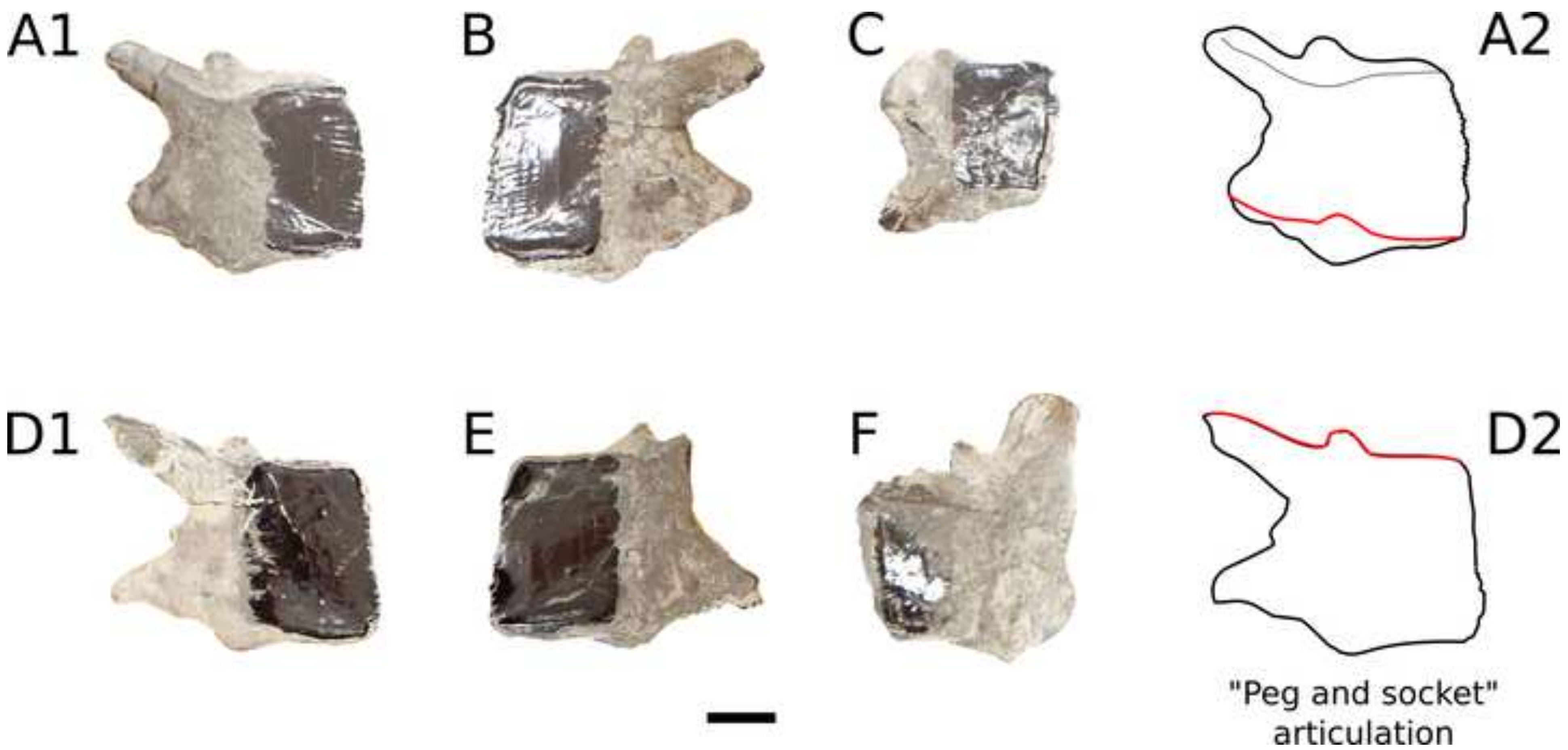
A1
B
C
A2
D1
E
F
D2
"Peg and socket"
articulation

Figure

Click here to access/download;Figure;FIG23_Lepidotidae.tif

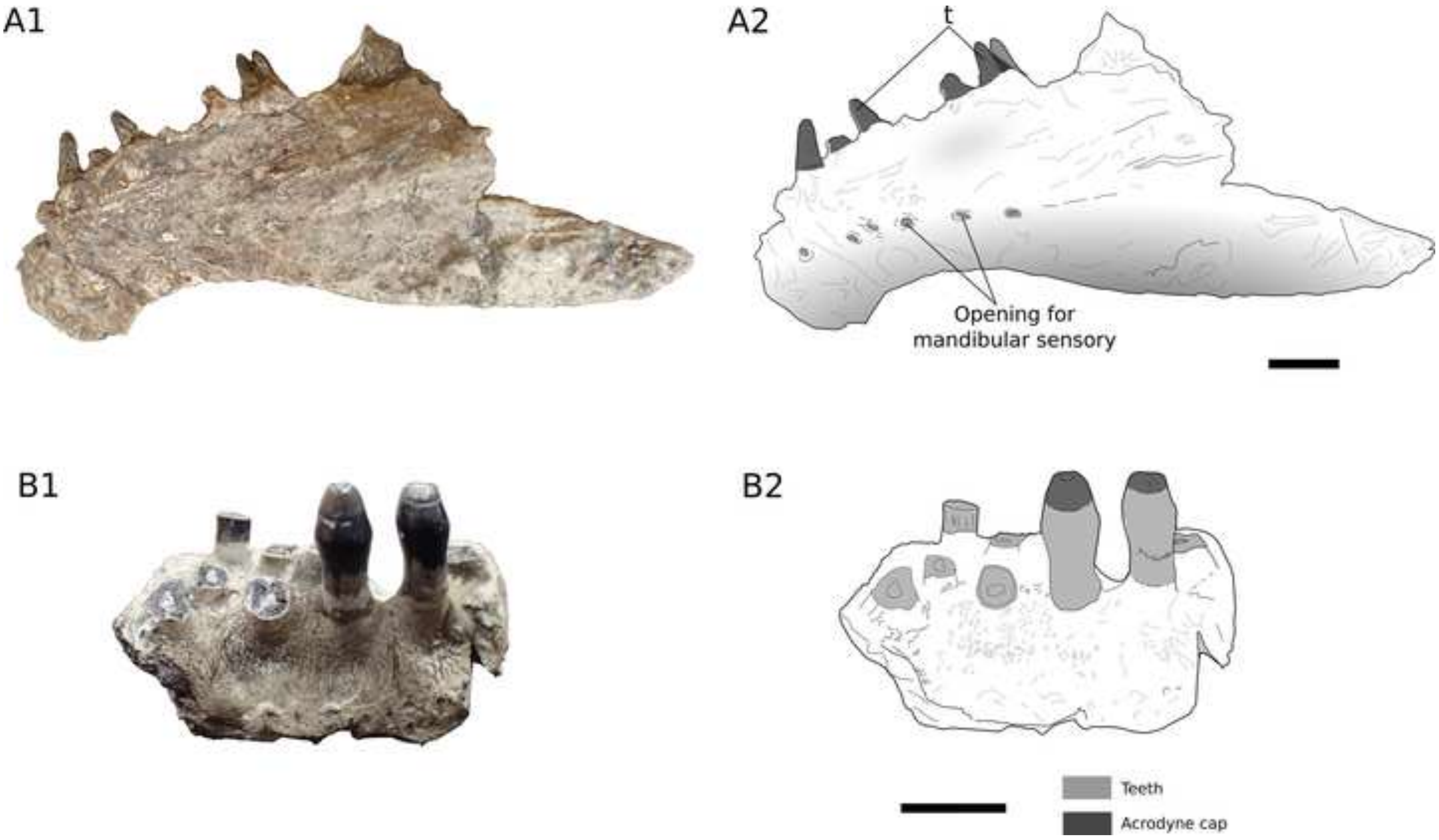

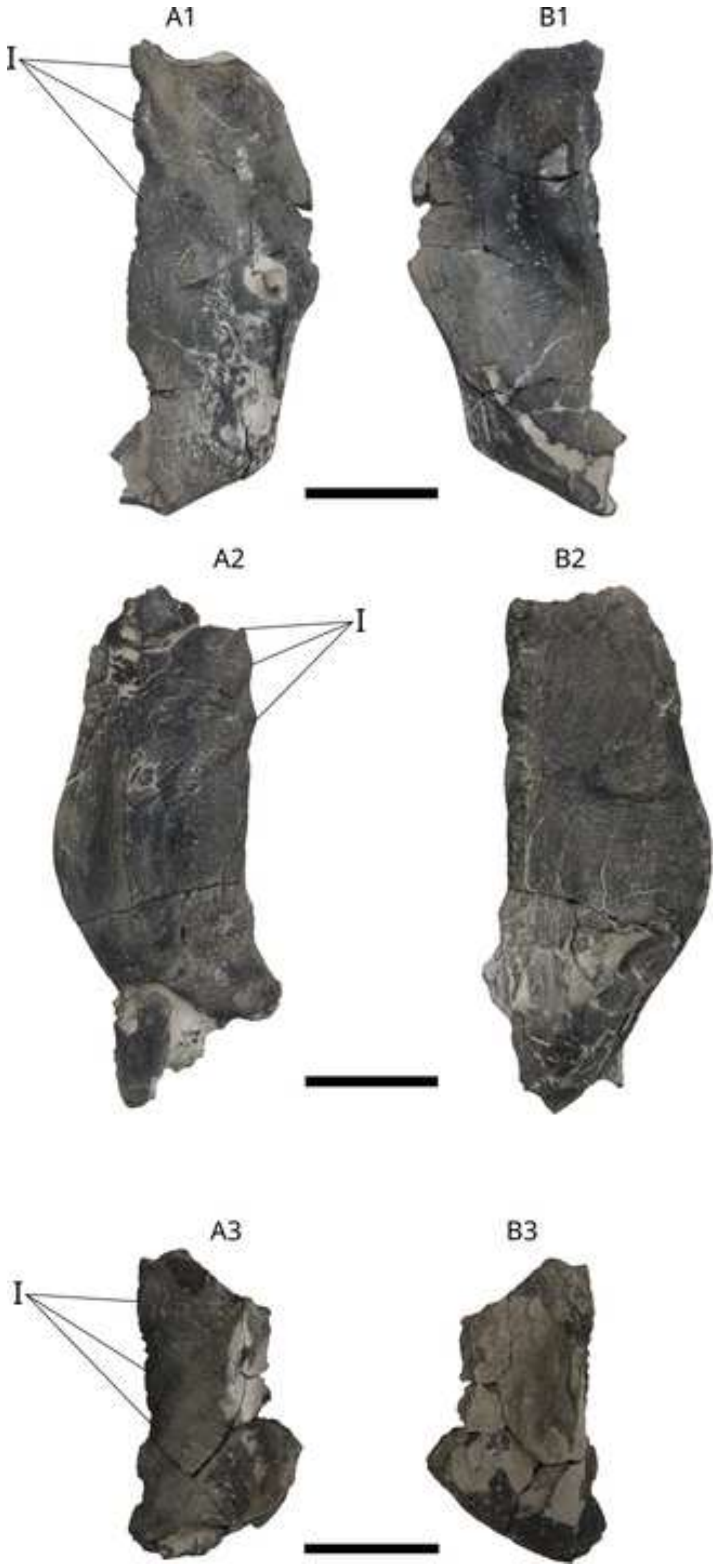
A1
B1
I
A2
B2
I
A3
B3
I

Figure

Click here to access/download;Figure;FIG13_Leedsichthys.tif

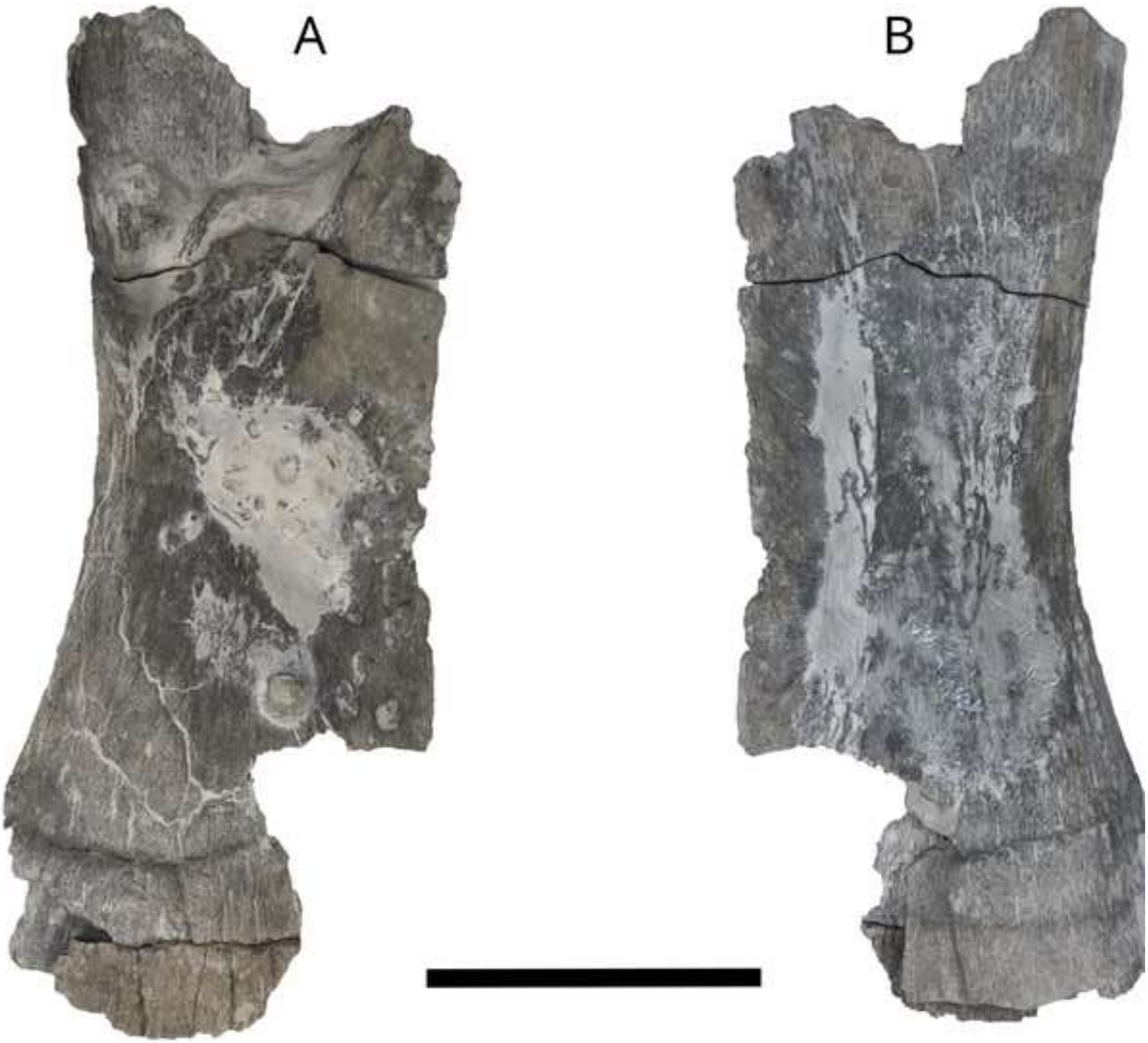

 Click here to access/download;Figure;FIG14_Leedsichthys.tif

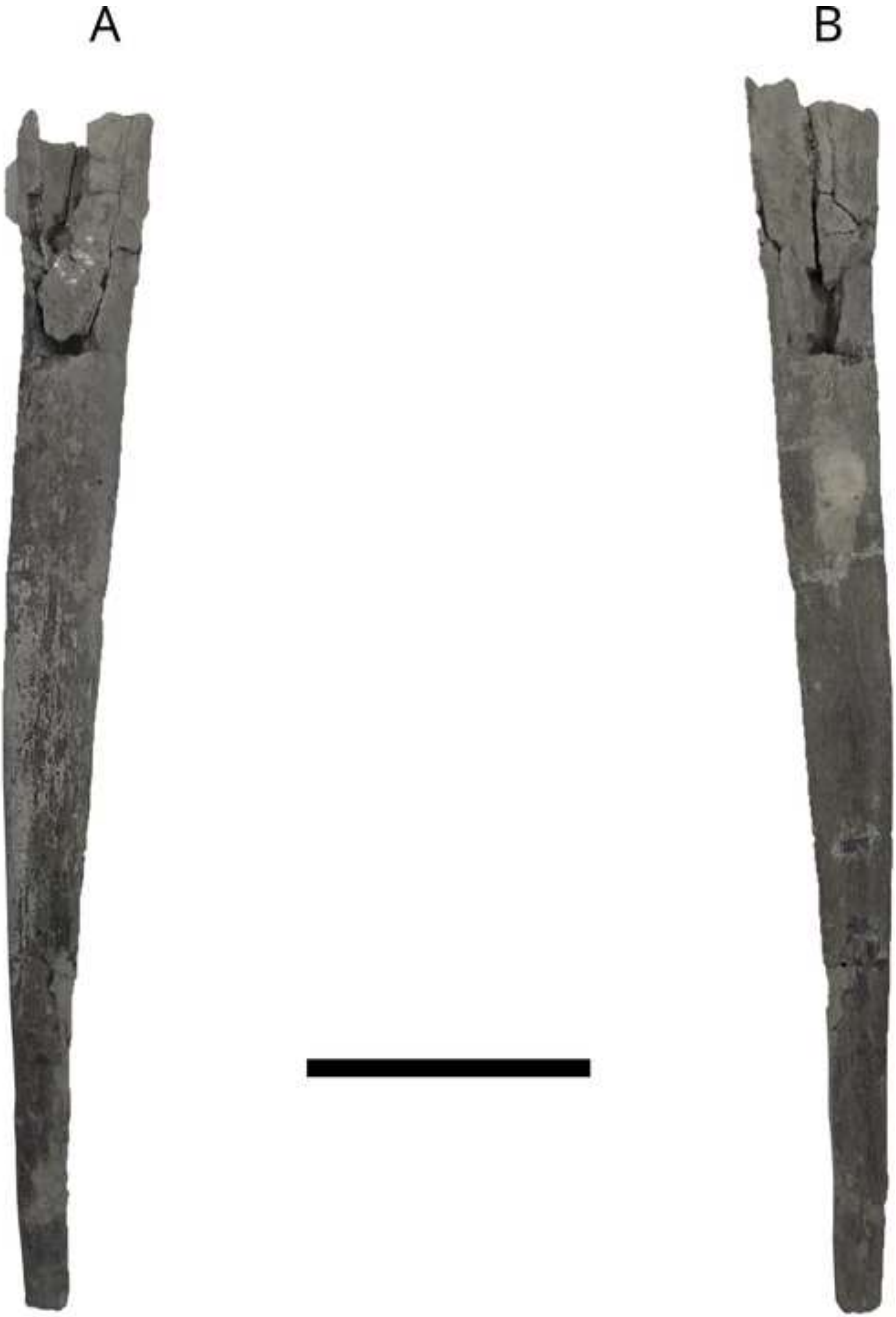

 Click here to access/download;Figure;FIG15_Hypsocormus.tif

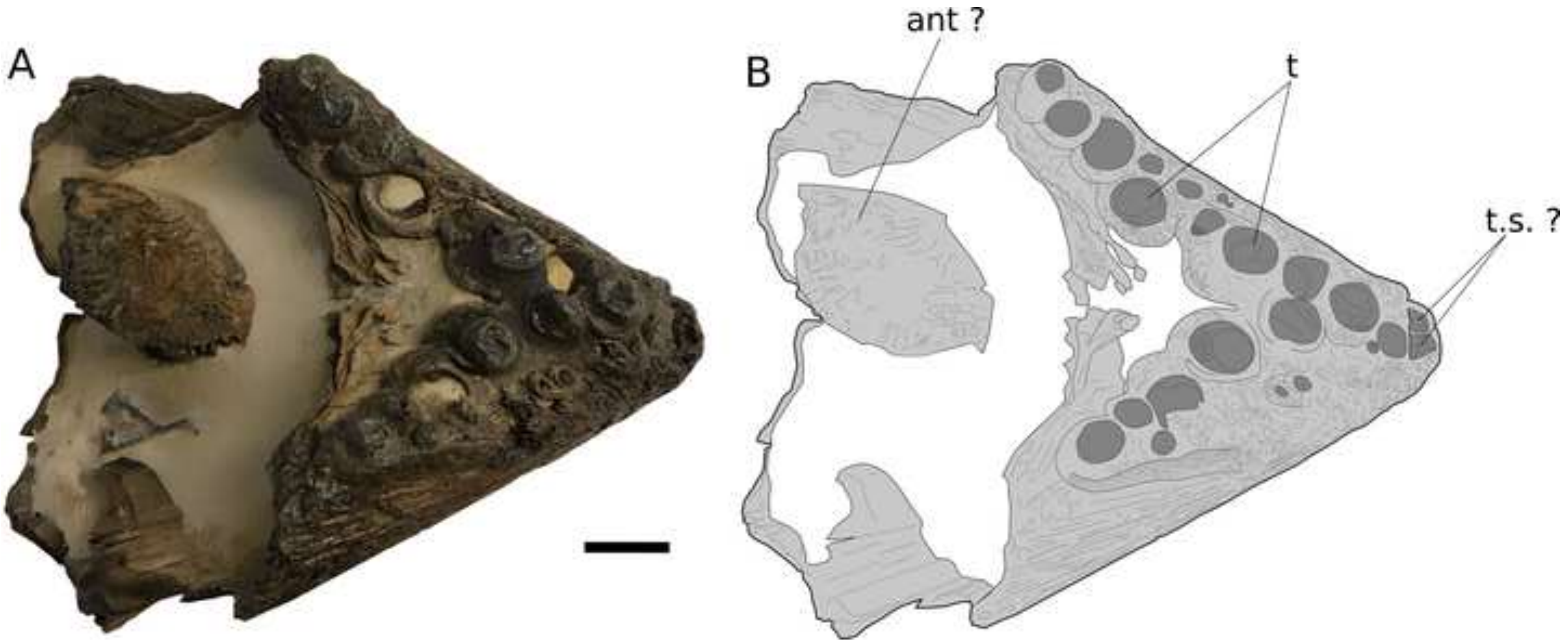

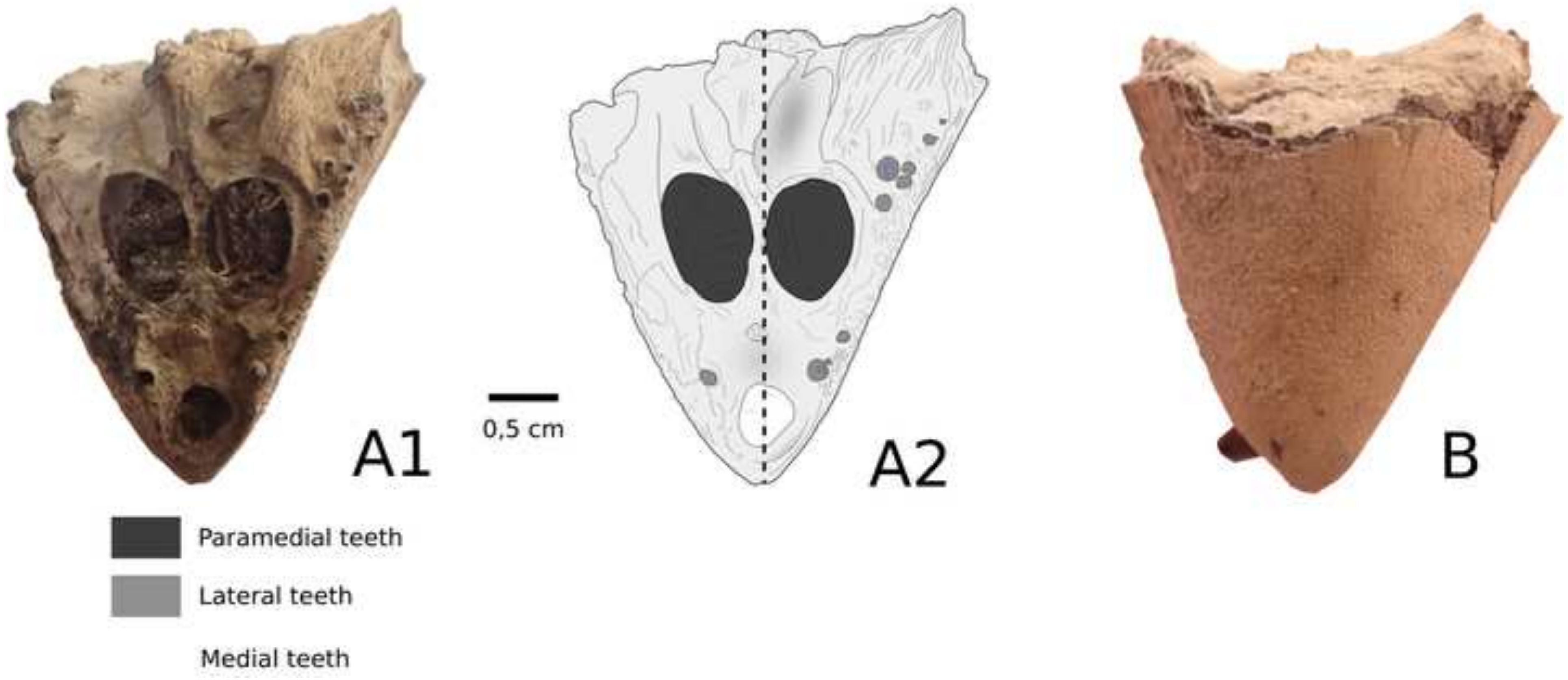
A1
0,5 cm
A2
B
Paramedial teeth
Lateral teeth
Medial teeth

Figure

Click here to access/download;Figure;FIG17_Hypsocormus.tif

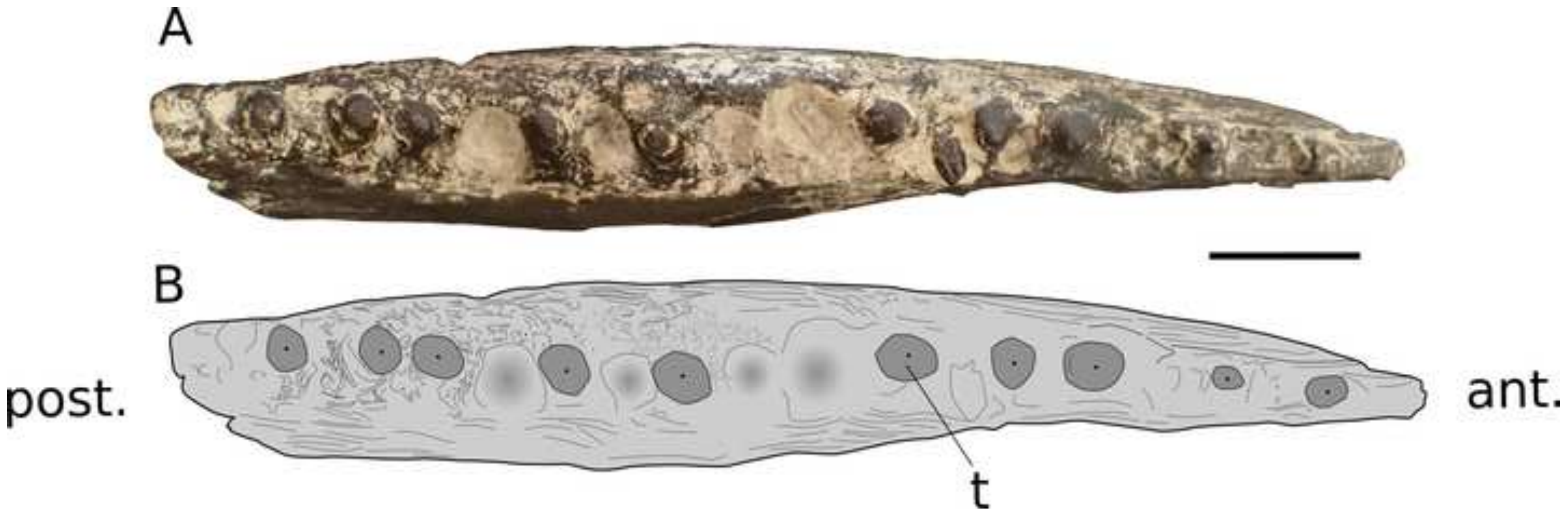

Figure

Click here to access/download;Figure;FIG1_Geol_Map.tif

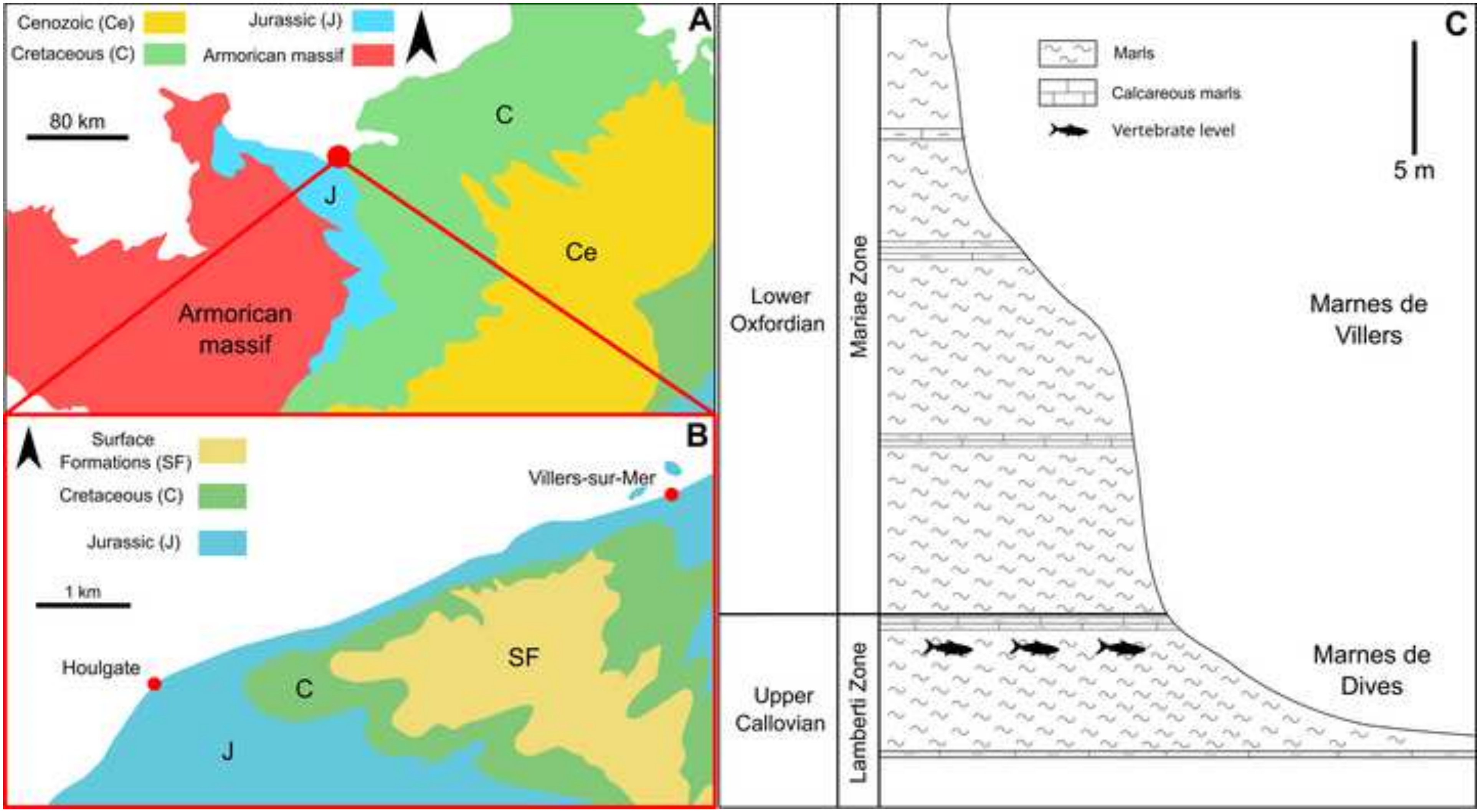

Supplement: Attachment — Submitted filename: PONE-D-25-45591_reviewer.pdf [file pone.0344519.s002.pdf]
