# Supplementary material for: Composition and paleoecology of the mid-Jurassic bony fish assemblage of the Vaches Noires cliffs (Normandy, France)
Source: PLoS One. 2026 May 27;21(5):e0344519. doi: 10.1371/journal.pone.0344519 (PMC13215526; doi:10.1371/journal.pone.0344519)
Supplement: Attachment — Submitted filename: Response to Reviewers.docx [file pone.0344519.s005.docx]

Dear editor, Dear Reviewers

We thank both reviewers for the very complete and constructive reviews.

Reviewer comments are in red, our responses are in black. Supplementary comments in text have been addressed.

The main text has been entirely restructured to fit systematics, with Actinopterygians first and Sarcopterygians at the end.

Response to reviewer 1:

- Coelacanths – It is interesting to see that coelacanth remains are present in the French Callovian, as these are one of the major Jurassic fish groups strangely absent from the English Oxford Clay Formation. Based on my recent work on coelacanth pterygoids, The specimens illustrated by the authors, in my opinion, look to be closer in morphology to Coccoderma (Laugiidae) than they do to Trachymetopon (Mawsoniidae). Please see my comments in the manuscript for further details. If the authors believe these remains to belong to a young mawsoniid, then they should explain their justification for ruling out Latimeriidae and Laugiidae as plausible candidates.

After discussion with Dr Cooper, we agree with him about coelacanths remain, but the very fragmentary nature of the Vaches Noires cliffs coelacanths make an identification to the genus *Coccoderma* very risky. Since *Trachymetopon* has already been found in the Vaches Noires cliffs, we prefer to keep our identification until more remains are found.

- Pycnodonts – this section is fine, but see my comments on the manuscript. Also note that the specimen in Fig 25C is figured in the wrong orientation (anterior and posterior opposed relative to specimens in parts A and B). the wider medial teeth are usually always located at the posterior in pycnodontiforms vomers/prearticulars which have well organized tooth rows.

We modified the pycnodont Fig 25C in accordance with the comments (Prearticular reoriented) and it has been redescribed after discussion between authors.

- Pachycormidae – the authors appear to give the youngest stratigraphic range for this group as ‘Hettangian’, listing Euthynotus and Pachycormus as present in the Sinemurian. However, there are no records of Pachycormidae prior to the Toarcian. Previous records of this family in the Sinemurian derive from Agassiz (1833-1844), although all of these pre-Toarcian occurrences have since been proven as invalid or highly dubious. I am also of the opinion that the authors’ hypsocormine sample is more diverse than is reported. Certainly, the rostrodermethmoid in Fig. 16 can, in my opinion, be referred with confidence to Hypsocormus leedsi Woodward, 1889, and the anterior coronoid in Fig. 19 with two enlarged coronoids tusks be referred to Orthocormus cf. tenuirostris (Woodward, 1889) (Hypsocormus tenuirostris in older literature. The authors also use outdated informal terminologies for the subfamilies (e.g., ‘toothed clade’ and ‘suspension-feeding clade’) please use formal clade names in the manuscript (Hypsocorminae + Asthenocorminae). The authors also make reference to a ‘Leedsichthys head’ in a private collection. This specimen is both intriguing and potentially significant. Is it possible for authors to figure this specimen, perhaps in the supplementary data?

After discussion with Dr Cooper, we agree with him about the Pachycormid issues, and we have changed some of our identification: Hypsocorminae from Sinemurian was a mistake, it has been removed. We refer the rostrodermethmoid to *Hypsocormus* cf. *leedsi*. We refer the coronoids fragments to *Orthocormus* cf. *tenuirostris*. Older terminology has been replaced by Hypsocorminae and Asthenocorminae. The *Leedsichthys* head will be discussed further in private with Dr Cooper, since the head is published, but it is in a private collection and is inaccessible. We removed this sentence from the text, since we haven’t heard from the specimen in many years, and it cannot really be analyzed.

- Lepidotidae – this section is sound, although I believe the specimen figured in Fig 21B-C is not a quadrate (also listed as a ceratohyal by the authors in the materials section), but instead the broken dorsal half of a hyomandibula. Please see my comments in the annotated manuscript for justification.

We reexamined the specimen Fig 21B-C, and we are not convinced by the Hypsocorminae identification. After careful examination, we concluded it corresponds more to a hyomandibula of Lepidotidae. It is reminiscent of *Lepidotes gloriae*. Since this specific fragment is very difficult to identify, we remained cautious in our determination.

- Discussion – this section needs some work and in some areas is flawed. The taxonomic comparisons between the French Callovian and other Jurassic marine localities are interesting and ambitious, but there are many inaccuracies in the faunal assemblages given by the authors. Between the text and the comparative faunal lists provided in Fig. 32, many taxonomic groups are missing from localities where they are known to be present (e.g., Chondrosteidae, Saurichthyidae, Coccolepididae missing from Toarcian) and others misattributed, or attributed without evidence (e.g., Pachycormidae in Sinemurian; aspidorhynchids in Toarcian [perhaps conflated with saurichthyids?]). The authors do not provide a faunal description of the Sinemurian fauna (I assume based on the Lyme Regis + Charmouth Blue Lias Group?) as they do for the other localities included in Fig. 32. I have given some detailed comments on the faunal assemblages of these sites in the manuscript.

The discussion section and Fig 32 have been completely revised. We now include Lyme Regis locality, with an dedicated section in the text. All missing taxonomic identification have been added.

To reviewer 2:

- *Eurypoma is not within Caturoidea (see for example López-Arbarello and Ebert 2023)*

*Eurypoma* phylogeny placement has been corrected

- Why do you think your specimens of Fig. 25 belong to the genus Athrodon? A label or a supposed identification by S. Wenz is not sufficient.

Pycnodont vomers are neither *Gyrodus* nor *Mesturus* due to uncrenulated nature of the teeths. *Eomesodon* and *Proscinetes* have ellipsoid teeth, where our specimens possess rounded teeth strikingly similar to *Athrodon wittei* and to the prearticular founded in the Marnes de Dives, both figured by Kriwet, 2008.

- Do not use the taxon name Lepidotidae (see for example López-Arbarello 2012).

Lepidotidae is a valid taxon since Lopez-Arbarello and Wenckler, 2016 (Neopterygian phylogeny the merger assay)

- Aspidorhynchiformes and Amiidae are not present in the Toarcian.

Aspidorhynchiformes and Amiidae in Toarcian has been removed

- Do you have any evidence for Orthogonikleithridae in the Callovium? I only know this Family from the Upper Jurassic

+ Ichthyodectidae are only present in the Cretaceous (see Cavin et al. 2013)

+ The taxon name Pholidophoridae is no longer in use for the Middle and Upper Jurassic. Grimmenodus is only found in Northern Germany and not in Holzmaden.

+ The name Paraleptolepis Arratia & Thies, 2001 was preoccupied and was changed into Longileptolepis Arratia, 2003.

All concerns in Fig 32 have been addressed (*Paraleptolepis* into *Longileptolepis*, Orthogonikleithridae in Callovian and all the mentioned above).

- Your Sinemurian locality in Fig. 32 is probably Lyme Regis. Here, many taxa are missing (Ophiopsiformes like Furo, Heterolepidotus, Osteorhachis; Leptolepidae…).

The Sinemurian locality has been added (Lyme Regis) with a description in the main text.

- Ophiopsiformes are missing in all localities + For Latimeriidae in Holzmaden see Cooper 2025 + For Coccolepididae in Holzmaden see Cooper et al. 2024 + Dapedium into Dapediidae

The same comments have been made by the first reviewer for Coccolepididae and Latimeriidae in Holzmaden. These taxa have been added, just as Ophiopsiformes in all localities and Halecomorphi genera such as *Heterolepidotus* or *Osteorachis*.

For Both reviewers:

- From reviewer 1: Teleostei – Based on the size and gross morphology of the illustrated chordocentra and given the groups present in the Callovian from neighboring sites, leptolepids are a plausible candidate, more so than pholidophoriforms which do not have these true chordocentra.
- From reviewer 2: Your “Teleostei” vertebra seem too large for Teleostei of the Middle Jurassic. The only teleost in the Middle to Upper Jurassic with completely ossified vertebra centra of this size (~5 mm) is the Ichthyodectiform Thrissops (see for example Nybelin 1964, Ebert 2025). But to me these vertebrae look completely different. To me the vertebrae in Fig 28A looks more like those in the halecomorph Amiopsis or Ionoscopus with the typical two deep depressions (see Grande & Bemis 1998).

Fig. 28B-E: For me, the figured vertebrae look more like chondrichthyan vertebrae. These vertebrae are too large for teleost vertebrae of Jurassic. The larges Thrissops vertebrae from the Upper Jurassic (Kimmeridgian/Tithonian) are approximately 7 mm wide und 8 mm high and they were very rare. Mid-Jurassic Ichthyodectiformes were presumably smaller and all other teleost with completely ossified vertebra centra at that time were not larger than 10 to 20 cm. Thrissops vertebrae have a small outer rim that immediately descends to the centre.

Teleosts are most probably present, but you should look for teleost vertebrae in the sizes <5 mm. These small vertebrae are often overlooked or simply not sampled.

Comments in the text about syntax have been considered and modified. Both comments about ‘Teleostei’ vertebrae are well founded and we thank the reviewers for the constructive review. Since both reviews were diverging on the identification, we asked another opinion from Gilles Cuny, expert in chondrichthyans. It seems that without thin section, it is practically impossible to determine whether the vertebrae are Chondrichthyans or Actinopterygians, but some of them, especially the one in Fig 29 is identified as Actinopterygian. We agree on the *Amiopsis* similarity, comments in text have been made on this point.

We have agreed to identify most vertebrae as undetermined actinopterygians. We think this is not the core information of the article, and some vertebrae are small, indicating small fishes, the latter being eaten by larger fishes the same way if they were teleostean, halecomorphs or any other kind of actinopterygian, which is the main information to retain.

To editor:

- Fig 1 is a map remade entirely by S. Beaufils and is made by using free online and open access resources from BRGM., which have an open-science policy: <https://www.brgm.fr/en/activities/knowledge-dissemination-open-science>
- Following the link <https://infoterre.brgm.fr/page/conditions-dutilisation-donnees> you can download the ETALAB licence ouverte pdf information <https://www.etalab.gouv.fr/wp-content/uploads/2017/04/ETALAB-Licence-Ouverte-v2.0.pdf> regarding BRGM copyright policies, which permit reuse and derivative works with attribution.
- Fig 14 is properly cited
- Reviewers’ recommendations about literature have been followed according to relevance and to our responses
