# Supplementary material for: Composition and paleoecology of the mid-Jurassic bony fish assemblage of the Vaches Noires cliffs (Normandy, France)
Source: PLoS One. 2026 May 27;21(5):e0344519. doi: 10.1371/journal.pone.0344519 (PMC13215526; doi:10.1371/journal.pone.0344519)
Supplement: Attachment — Submitted filename: Response_to_Reviewers_auresp_2.docx [file pone.0344519.s006.docx]

Dear editor, Dear Reviewers

We thank both reviewers for the last comments

Reviewer comments are in red, our responses are in black. Supplementary comments in text have been addressed.

Last comments have all been implemented, from both reviewers.

Comments from Dr Cooper :

819 – I do not know why previous authors have scaled up these Trachymetopon specimens based on Axelrodichthys when they refer them to Trachymetopon. Trachymetopon liassicum, the type and only species is based on a virtually complete and fully articulated specimen from Ohmden, housed in the University of Tubingen.

It’s because we had a well-made reconstruction of the skeleton of A. araripensis at our disposal. Furthermore, all these coelacanths, especially Mawsoniids, have a pretty conservative morphology. We think that reconstructing size either with Trachymetopon or Axelrodichthys will give the same results.

Line 380 – do not need commas ‘,’ before word “in Normandy” as ‘in’ is a bridge word.
Line 381 – Early (spelling).
Line 522 – Orthocormus cf tenuirostris should have (Woodward, 1889) in brackets as he originally described this species as Hypsocormus tenuirostris
Line 589 – lower case ‘a’ in “actinopterygians” – change “who” to ‘that’
chondrichthyians and actinopterygians should be lower case as they are informal taxonomic names, unless at the beginning of a sentence.
594 – Do the authors mean ‘centra’ (pleural)?
594 – change “ thin section: to ‘thin sectioning’ or ‘histology’
595 – change “state” to ‘differentiate’
758 – there are several large pliosaurs in the oxford clay that could have injured the tail fin specimen, perhaps edit slightly to say “following a probable attack by a large pliosaur, like Liopleurodon ferox.”
808 – change to “Halecomorphi-like” or “halecomorph-like”
809 – change to ‘teleosteomorph-like fishes’ or just ‘teleosteomorphs’.
813 – change to either “other genera” or “another genus”.
814 – Kimmeridge Clay Formation
814 – remains (pleural) – consider changing to say “…too incomplete to confidently refer to genus level”

868 – Lyme Regis is the name for one small town along the Jurassic coast, it is not called the Lyme Regis coast. Change to just ‘…area of Lyme Regis (Dorset)’.
868 – change to ‘Holzmaden area’ as this incorporates a lot of the nearby localities where most of these taxa are found – e.g., Ohmden, Bad Boll.
876 – delete comma after ‘ranges’ ‘..represents a shallow…’
879 – Ptycholepis spp. – present are Ptycholepis curta (curtus), P. monolifer, P. minor ect.
881 – specifically by Chondrosteus acipenseroides Egerton ex. Agassiz
882 – Tetragonolepis is (to my knowledge) not found in Lyme Regis – Tetragonolepis is a Toarcian genus named by Bronn (1830) which Agassiz used for describing his species that would later be moved to Dapedium Leach, including those from Lyme Regis.

886 – change “at Toarcian” to “in the Toarcian”
893 – say “The Posidonienschiefer Formation, predominately quarried from the Holzmaden area near Stuttgart, is widely distributed across Baden-Württemberg.
889 – delete ‘basal’
889 – also a true hypsocormine based on an isolated mandible, Sauropsis veruinalis may represent an additional hypsocormine
891 – delete “a” after Ohmdenia (Saurostomus and Germanostomus are also transitional forms, although less derived than Ohmdenia).
904 – ‘has also been described by Cooper (2025)’
909 – Acipenseriformes
948 – consider revising as “The Kimmeridge Clay also contains a large indeterminate acipenseriform (Chondrostei)”
948 – Teleosteomorpha is represented by…
1004 – change “in analysis” to ‘under study’ and delete the following sentence, and change in the following sentence “this taxon” to “Eurypoma’ grande.

All these comments above about grammar have been taken into account and changed in both manuscript and manuscript with track changes

884 – what do you mean by yielding more than 50 type species? 50 type species from Lyme Regis?

Yes. In Dineley and Metcalf, 1999, it is said, I quote “Lyme Regis is the type locality for 50 or more species of fish.”

Comments from Reviewer 2 :

In Fig. 32: Dapedium and Tetragonolepis are not Halecomorphi

Fig 32 has been modified in accordance with this comment. Dapedium and Tetragonolepis have been moved to a line of their own, the Dapediidae. The figure has been downloaded and is replacing the old Fig 32.
